# Michotte’s research on perceptual impressions of causality: a registered replication study

**DOI:** 10.1098/rsos.250244

**Published:** 2025-09-15

**Authors:** Peter White

**Affiliations:** ^1^ Department of Psychology, Cardiff University, Cardiff, UK

**Keywords:** phenomenal causality, launching effect, causal perception, Michotte, causal impression, causal cognition

## Abstract

Michotte (Michotte A, 1946 La perception de la causalité. Louvain: Études de Psychologie. Michotte A, 1954 La perception de la causalité, 2nd éd. Louvain: Études de Psychologie. Michotte A, 1963 The perception of causality. (Transl. by TR Miles, E Miles). London: Methuen: English translation of Michotte) showed that visual impressions of causality can occur in perception of simple animations of moving geometrical objects. In the launching effect, one object is perceived as making another object move by bumping into it. In the entraining effect, the two objects move together after contact and the first moving object is perceived as pushing or carrying the other one. There has been much further research on the launching effect in particular, and citations of Michotte’s pioneering work have increased rapidly in recent decades, underlining its importance in contemporary psychology and neuroscience. However, many of the experiments reported in Michotte’s book, exploring conditions under which launching and entraining do and do not occur, have never been replicated. The methodology, involving mostly a few knowledgeable observers and no statistical analysis, indicates that replication and extension would be desirable, to assess the reliability of the results reported by Michotte and to inspire further research on aspects of these perceptual impressions that have been neglected in more recent research. In this pre-registered replication study, 14 experiments are reported that replicate and, in some cases, extend experiments reported by Michotte (Michotte A, 1946 La perception de la causalité. Louvain: Études de Psychologie. Michotte A, 1954 La perception de la causalité, 2nd éd. Louvain: Études de Psychologie. Michotte A, 1963 The perception of causality. (Transl. by TR Miles, E Miles). London: Methuen: English translation of Michotte). Some findings reported by Michotte were replicated, others only partly so, and in other cases results were different from what Michotte reported. In particular, results on the delay manipulation differed from those reported by Michotte. Results show the great importance of the entraining and pulling impressions, which have hitherto received much less attention than the launching impression. Extensions to Michotte’s experiments revealed numerous new findings and open up prospects for much more innovative research. The results also have significant implications for possible explanations for perceptual impressions of causality.

## 1. Introduction

**Figure 1.**
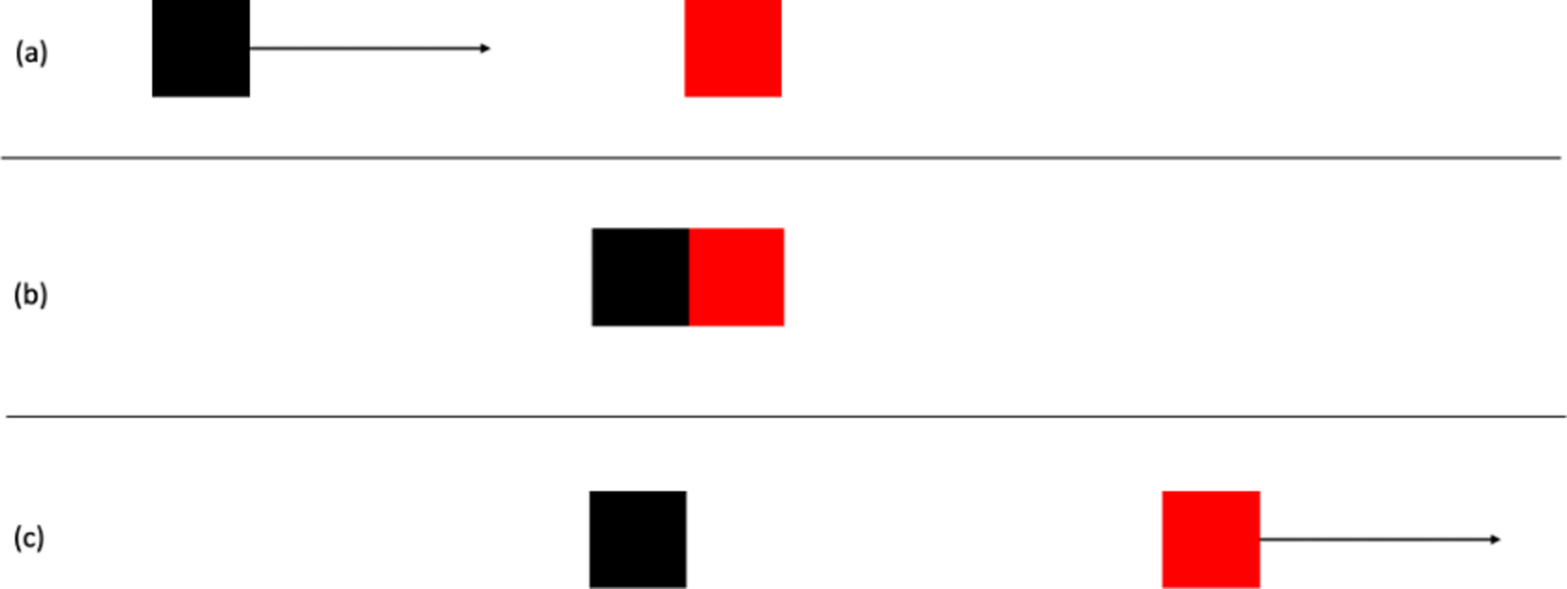
Schematic representation of stimulus for the launching effect used by Michotte [1]: (a) initial locations of objects and motion direction of the black square; (b) contact between the objects, at which point the black square stops moving and the red square moves off as shown in (c).

When observing simple animations of moving geometrical shapes, we sometimes have perceptual impressions of causality, of one object making something happen to another object. This was first demonstrated by Michotte [1–3]. In his stimulus, a black square (object A) and a red square (object B) are visible, as shown in figure 1. Figure 1a shows the initial locations of the objects. The red square is initially stationary. The black square moves horizontally at constant speed until it contacts the red square, whereupon it stops as shown in figure 1b. Without delay the red square moves off at the same speed and in the same direction, as shown in figure 1c. The stimulus is deliberately highly abstracted. The objects are simple two-dimensional geometrical forms and there is no visual context. It might be expected that observers would perceive only the objects and their motions. In fact, in the English translation of Michotte [1], ‘observers see object A bump into object B, and *send it off* (or '*launch*' it), *shove it forward, set it in motion, give it a push*. The impression is clear: it is the blow given by A which *makes B go*, which *produces* B’s movement’ (p. 20) [1–3] called this perceptual impression the launching effect (*l'effet lancement* in the original publication).

In a variation on that stimulus, the black square continues to move after contact with the red square, so that the two objects move together, remaining in contact. The reported impression is that the black square pushes or carries the red square. Michotte called this the entraining effect. Launching and entraining are both causal impressions, but are qualitatively different. The entraining impression shows that there is more to perceptual impressions of causality than just the launching effect, and indeed there may be multiple qualitatively distinct visual causal impressions [1–5].

The aim of the present research was to replicate, with extensions in some cases, several of the experiments on the launching and entraining effects reported in [1–3].

The launching effect is well established and has been confirmed in numerous subsequent studies [4,6–9]. Evidence from neuroscience, perceptual processing and developmental studies converges on the conclusion that the launching effect is a perceptual phenomenon, generated in automatic perceptual processing, not a product of post-perceptual cognition. In neuroscience, it has been found that typical stimuli for the launching effect activate areas in the visual system of the brain, distinctively from non-causal control stimuli [10–12]. The perceptual nature of the launching effect is shown by evidence that it can influence other contemporaneous perceptual processing. Moors *et al*. [13] used a method called continuous flash suppression, in which a dynamic noise stimulus is presented to one eye and a stimulus of interest is presented to the other eye with gradually increasing contrast, until the participant reports detection of any part of the stimulus. Participants did not have to report a causal impression, just any element of the stimulus. Detection occurred sooner for launching stimuli than for non-causal controls, supporting the hypothesis that causality is constructed at an early stage of perceptual interpretation.^1^ Typical stimuli for the launching effect induce retinotopic adaptation, meaning adaptation specific to the retinal location to which the stimuli were presented [14,15], also indicative of the causal impression being a product of perceptual processing. If a stimulus is presented in which the black square stops before reaching the red square and the gap between them is filled with a stationary object, the size of the gap is underestimated, as compared with non-causal control stimuli [16]. That illusory spatial contraction is greater at the end of the stationary object contacted by the black square than at the other end, further indicating involvement of perceived causality in generating the illusion [17]. The perceived trajectory of apparent motion varies depending on whether the objects in question are causal objects in a launching display or not [18], showing that the causal interpretation occurred prior to, and influenced, the construction of apparent motion. Developmental evidence also supports the claim that the launching effect is a perceptual phenomenon: infants aged about six months respond to launching stimuli and non-causal controls as if a causal impression has occurred with the launching stimulus [19–22].

The causal impression does not correspond to what the laws of physics tell us about interactions between inanimate objects. Newton’s Third Law states that objects at contact exert equal and opposite forces on each other. It is as true to say that the red square makes the black square stop as it is to say that the black square makes the red square move. But participants in experiments do not perceive the red square as making the black square stop, and do not mention that possibility in spontaneous verbal reports of their perceptions [1–3,8]. Causality is perceived as going one way, from the black square to the red square [23]. The black square is incorrectly perceived as exerting more force on the red square than the red square exerts on the black square [24,25]. The typical stimulus for the launching effect, in which the red square moves at the same speed as the black square, is not even very realistic. Runeson [26] showed that it lies at one extreme of the range of possibilities allowed by the laws of mechanics, an extreme that would never be encountered in actual collision events. Normally, the object in the role of the red square would move more slowly than the object in the role of the black square, not at the same speed, and the latter would continue to move forward rather than stopping on contact. The typical stimulus for the entraining effect is also unrealistic because the two objects could only continue to move together without change of speed if the red square had zero mass and the black square adhered to it. Whatever the launching and entraining effects may be, they are not direct or accurate apprehension of what goes on in real inanimate contact events.

Michotte’s pioneering research on perceptual impressions of causality has been hugely influential. It has been described as ‘classic’ (e.g. by [13,27,28]) and ‘seminal’ [29], and it continues to influence and inspire research in perception, cognition, developmental psychology, social psychology, cross-cultural psychology, treatment of causality in language, and also in neuroscience [4,7,9,14]. Interest in Michotte’s research on visual causal impressions is rapidly increasing. Michotte’s book reporting the research was first published in French in 1946, with an extended second edition published in French in 1954, and an English translation of the second edition published in 1963; from this point on only the 1963 edition will be cited because it was the source consulted by the present author. Wagemans *et al*. [30] reported that the various editions of the book had, in 2006, been cited 419 times, and they reported data showing a steady increase in citations over the decades. That increase has accelerated since then: consultation of the Web of Science (on 21 April 2023) shows 1389 citations of the book, so the number has more than tripled in just 17 years.

Michotte [1] reported 95 experiments and numerous additional observations not dignified with experiment numbers. Of the numbered experiments, 44 were concerned with the launching effect, nine with the entraining effect and the remainder with various other phenomena such as perception of animal locomotion and qualitative causality (e.g. whether a contact event can be perceived as causing a change in size of an object, without that object moving). Many of the experiments on launching and entraining have never been replicated, and have received little attention in the subsequent research literature. Given the long-standing and ever increasing importance of Michotte’s research in general and that on the launching effect in particular [4,7,30,31], this is an unsatisfactory situation. The reproducibility of many of the results described by Michotte [1] is not known; also, there is potentially a rich treasure trove of research there, and re-examination of it holds the promise of expanding the scope of research on perceptual impressions of causality.

It is not feasible to replicate all of the experiments on launching and entraining. It was decided to focus on experiments most directly concerned with the causal impressions themselves. Experiments on matters peripheral to the causal impression, such as those on the radius of action (the span of movement on either side of the contact event that seemed to observers to have something to do with the contact event) were not selected. Fourteen experiments were designed, eight on the launching effect and six on the entraining effect. Most of these were concerned with experiments by Michotte that have never been replicated or extended. Two of them concern variables that have been further investigated but with results that have varied considerably between studies. These are delay between the black square contacting the red square and the red square starting to move, and spatial gap between the red square and the location at which the black square stops. Research on those variables is summarized in the introductions to the respective experiments.

### 1.1. Pre-registration and open science

I confirm that the study was registered prior to conducting the research and the pre-registration adheres to the disclosure requirements of the institutional registry. The link to the OSF project for this research is: https://osf.io/5dygp.

This project received Peer Community in Registered Report Stage 1 in-principle acceptance, after which the Stage 1 manuscript was uploaded to OSF: https://osf.io/kynjw.

All measures and manipulations for this project are reported in the accepted Stage 1 manuscript and the studies were carried out as specified there. All pre-registered analyses are included in this manuscript and there are no analyses that were not pre-registered. Data collection was completed before any data were viewed or analysed by the author. Stimuli and software for stimulus generation have been uploaded to the OSF project for this research and can be accessed at the link to the project above. Raw data have also been uploaded to the OSF project and can be accessed in the same way.

## 2. General features of method

The experiments reported in Michotte’s book were not conducted in accordance with present-day understanding of methodological rigour. In many experiments, the only participants were Michotte alone or Michotte and two experienced and knowledgeable colleagues. In a few, a sample of naive observers took part, but the reports are short on information about the participants, the instructions given to them, and data recording. There is no statistical analysis. In some experiments (such as the delay experiment), there are reports of percentages of observations falling into one category or another, but that is all. Michotte’s preferred approach was experimental phenomenology: the aim was to capture the qualitative features of perception and, in some experiments, how those features varied with stimulus conditions, the ultimate goal being to construct a theoretical account of the perceptual structure of phenomenal causality. Using an experienced observer was considered a more fruitful means of achieving that goal. Without meaning to denigrate experimental phenomenology, replication with a large sample of naive participants would be desirable.

Most of the stimuli were created using an ingenious mechanical apparatus involving paper discs mounted on a rotating spindle. The ‘objects’ were thick lines painted on the discs, and they appeared as rectangles to the observer because a screen was interposed in front of the discs. A narrow slit in the screen revealed to the observer just a short segment of each line, creating the appearance of small rectangular objects. When the disc rotated, the objects appeared to move or stay still depending on how the line was painted on the disc. The slit formed a visible track along which the objects appeared to move. In other experiments, a cinematic projection method was used. The present research used computer technology instead of Michotte’s apparatus. Most studies since Michotte have used computer presentation and the launching effect clearly does occur with that technology. It is possible that technological differences could affect the results; this issue is addressed in the general discussion in light of the results.

In visual appearance, the stimuli and manipulations were as similar as possible to those used by Michotte. The object that moved first in the stimulus for the launching effect was a black square and the other object was a red square and those features were retained, except where object shape was manipulated. The standard size of object used by Michotte (with the rotating disc method) was 5 mm square. A larger size of 12.4 mm (40 pixels) was used in the present research, except where object size itself was a manipulated variable. There was no visible slit or track: the objects moved in an otherwise plain white frame on the computer screen. The viewing distance reported for the basic launching effect experiment was 1.5 m and that was retained. In keeping with Michotte’s method, movement of the heads of observers was not restricted.

Instead of spontaneous reports of perceptual impressions, the present research used rating scales. Rating scale methods have been used in many studies on perceptual impressions of causality [4] and are an accepted method of collecting data on perceptual impressions under many circumstances. For purposes of replication, the rating scales should capture the forms of words used by Michotte when describing the perceptual impressions. There is inevitably a risk that verbal statements may be interpreted by participants in ways that are different from what they meant to Michotte. However, construct validity requires wording of rating scales to be as similar to Michotte’s descriptors (in English translation) as possible. The participants cannot be trained in Michotte’s method of experimental phenomenology, and in any case it is important that they should be naive to the research and not influenced by possible bias on the part of the researchers. Asking participants to give free verbal reports of what they perceive (as in [8]) essentially transfers the problem of interpretation from the participant to the researcher. For any kind of statistical analysis to be done, the participants' reports would need to be subjected to content analysis. Defining the content categories in advance so as to ensure validity in categorization of statements is problematic. And participants cannot be guaranteed to focus on the features of the stimulus that are of interest to the researcher: for example, they might not report a causal impression even if one occurred, but might ignore it and report just the motions of the objects instead. So rating scales were used that take the form of verbal statements based on Michotte’s descriptors, and participants rated their degree of agreement or disagreement with each statement. Different statements were used in different experiments, so further details are given in the method sections of the respective experiments.

Michotte reported that the launching and entraining effects are not always reported by naive observers at first. He claimed that, after a few trials, the causal impressions did start to occur, and that the initial problem was due to the participants not being used to the artificial conditions of the laboratory, probably including the mechanical apparatus used to present the stimuli. Two subsequent studies with naive participants and the same apparatus reported low rates of reporting the launching effect [32,33]. Effects of experience with the stimuli have also been found [8,34–36]. As Scholl & Tremoulet [9] argued, those findings can be interpreted as response biases, in other words as effects on how people make overt responses about what they perceive, rather than effects on the perceptual impressions themselves. There may also be effects of fatigue and attention [37]. Participants may be reluctant to endorse extremes of the rating scale until they have seen a representative sample of the stimuli, to get an idea of the range of variation in them. On the other hand, Bechlivanidis *et al*. [38] found that gap and delay stimuli shown before participants have observed a typical launching effect stimulus tended to be given high ratings of causality, and those ratings fell significantly after exposure to a typical launching stimulus. More will be said about that study in the introduction to Experiment 4 below. It is, however, important to the replication study that participants should, as far as possible, report what they see, their visual impressions, and not what they think following deliberation. Preliminary experience with the stimuli, and carefully worded instructions, are both important to achieving that end. The plan, therefore, was to start by presenting each participant with a sample of six stimuli chosen to illustrate the variety of stimuli that would be encountered. Participants just viewed each stimulus, presented in random order and no response was elicited from them. Two of the six were the typical stimuli for the launching and entraining effects.

In experiment 38, Michotte [1] manipulated the speed of the objects, with both moving at the same speed, from 4 to 1100 mm s^-1^. He reported: ‘The most perfect impression of launching is given with speeds between 20 and 40 cm. per sec. [200 to 400 mm s^-1^] and even a little higher’ (p. 107). At speeds of approximately 100–150 mm s^-1^ he reported that ‘the impact is slight and lacking in vigour’ (p. 107), though the launching effect still occurred. With Michotte’s apparatus the apparent motion was macroscopically continuous. With computer-generated stimuli that is not the case. The stimulus is a series of static images replaced at the refresh rate (60 Hz in the present study), and at high speeds one image is displaced by several pixels from the one in the previous frame. The very high speeds that supposedly gave rise to the strongest impressions of launching are not practical with computer presentation because the large jumps from one frame to the next can give rise to noticeable blur or jerky motion. That could disrupt not only motion processing but also perception of contact between the objects. A compromise must therefore be found between the desideratum of high speed and the need for smooth motion and absence of blur to be perceived. With the technology to be used for the experiments, that compromise appears optimal at approximately 124 mm s^-1^. That was therefore adopted as the standard speed for the objects and was used except where indicated otherwise.

Stimulus variables either investigated or mentioned in Michotte’s reports of the experiments were manipulated, mostly resulting in parametric designs that could be analysed with analysis of variance (ANOVA). A large sample of naive observers took part, and the experiments were run by experimenters naive to the research topic, as well as to the specific aims and hypotheses being tested.

To conclude this section with a typographical convention, the experiments in the present paper are identified with upper case ‘E’ and Michotte’s experiments are identified with lower case ‘e’ (except at the start of a sentence).

### 2.1. Participants

It was not feasible to have different participants for each experiment because of resource limitations. The experiments were divided into two groups, each with a separate set of participants, as follows: group 1 included experiments 1, 5, 8, 10, 12, 13 and 15; group 2 included experiments 2, 3, 4, 6, 7, 9 and 11. This was partly to reduce the burden on individual participants and partly to enable comparisons between experiments where it was desirable for participants in one experiment not to know what was presented in another. Experiments 11 and 12 are an example; that and others are discussed in the individual methods and results sections. Order of presentation of the experiments was randomized for each participant. There were 50 participants in each group, making a total of 100. The participants were volunteer first-year undergraduate students of psychology at Cardiff University with normal or corrected to normal vision, participating in return for course credit. Michotte’s research is not on the undergraduate curriculum so it is likely that all were naive to the research topic. Of the participants, 83 identified as female, 12 as male and 5 did not disclose gender. Age and nationality were not recorded but, in the cohort from which participants were recruited, most were in the age range 18–21 years, and most had British nationality. Informed consent was obtained from all participants and participants were given a written debrief at the end of the experiment, as well as having the opportunity to ask questions about the research. Ethical approval was granted by the Ethics Committee of the Cardiff University School of Psychology.

## 3. Minimum effect size and sample size determination

This is a replication study and the research being replicated was not subject to any kind of statistical analysis. In view of that, the main concern is to establish statistical significance. The minimum effect size of interest is of less concern than finding statistically significant support for the effects claimed by Michotte. Avoiding both Type I and Type II errors is important. These considerations indicate that it is desirable to have a relatively large sample and a conservative alpha level of 0.01.

In principle any statistically significant effect would be meaningful no matter how small the effect size, but small effect sizes can only be detected by studies with large samples of data. Therefore it is reasonable to consider what sort of effect size can be expected and to determine the sample size in accordance with that. The minimum effect size of interest cannot be defined *a priori* but effect sizes in previous research can provide a reasonable empirical guide [39]. For this purpose, the published experimental research on phenomenal causality was scrutinized and studies were selected that met the following criteria: (i) effect sizes were reported (not many studies have done this); (ii) the measure used must be a causal judgement measure of the sort used in the proposed research, so, for example, studies of judged speed [40] and judged naturalness [41] were ruled out; (iii) ANOVA must be used and, since only main effects are predicted in the proposed studies, only effect sizes for main effects were sampled; and (iv) only effect sizes for effects where a significant effect was predicted were selected. Effect sizes meeting these criteria were found in the following studies: Mitsumatsu [42]; Ryu & Oh [43]; Vicovaro [44]; Mayrhofer & Waldmann [45]; Hubbard & Ruppel [46]; and I included my own most recent publication that met the selection criteria [47]. This generated a sample of 25 effect sizes with an overall mean of 0.40 and a range from 0.04 [42] to 0.73 [46]. Only three were less than 0.20 (all from [42]), and two more were less than 0.25, so 80% of the effect sizes were greater than 0.25. There is a possibility that the mean is inflated by publication bias [39] but, if small effect sizes were common, the distribution of effect sizes in published research should be skewed towards the smaller end of the range and there is no evidence of that in the effect sizes sampled here. It is likely, therefore, that true effect sizes for the phenomena studied in this research are often greater than 0.25.

With that in mind, G*Power was used to determine desired sample sizes for the designs of each of the proposed experiments (except for Experiments 8 and 10 where the chi-square test would be used). For these calculations, alpha was set at 0.01, power at 0.90, correlation among measures at 0.1, and non-sphericity correction at 1. With these values and an effect size of 0.20, the desired sample varied from 36 (for Experiments 7 and 9) to 66 (for Experiment 3). With an effect size of 0.25, the desired sample varied from 24 (for Experiments 7, 9, 11 and 12) to 42 (for Experiment 3). A sample of 66 was not possible because of resource limitations but a sample of 50 was feasible. With power at 0.20, only two experiments (2 and 3) have desired samples in excess of that and, with power at 0.25, none of them do. A sample of 50 for each experiment was therefore deemed adequate to give a reasonable chance of finding any effects that are there to be found.

A sample of studies using launching stimuli and published since 2000 revealed considerable variation in sample size. Several studies reported between 8 and 20 participants [18,19,27,40–43,48–50]. A few had more than 20 but had different dependent measures as a between-subject variable, with numbers varying from 14 to 16 for each dependent variable [51–53]. Of the remainder, in ascending order of numbers, Umemura [54] had 27; Vicovaro [44] had 40; Young *et al*. [55] had 44; Wang *et al*. [56] had 57, with 32 on a causal judgement measure and 25 on a force judgement measure; Young & Falmier [57] had 58; Falmier & Young [58] had 67 in a four-way mixed ANOVA design; Schlottmann *et al*. [8] had 72 in a study where the measure was free verbal reports; Mayrhofer & Waldmann [45] had 934 in an online study with 233 or 234 participants allocated to each of four between-subject conditions. Two points can be made about this. One is that it seems not to be difficult to obtain statistically significant results with small samples, as used in most of the studies cited above. The other is that the sample size of 50 chosen for the present research is towards the higher end of the range. Reliability is a major issue in a replication study and there are indications of substantial inter-individual variability in responses (e.g. [8,59]), so a large sample is desirable for those reasons as well.

Data from all participants was included in the analyses.

## 4. Apparatus and stimuli

Stimuli were generated on screen using PsychoPy (version 3 [60]) from instruction files written in Excel. Stimuli were presented on an iMac desktop computer with a screen resolution of 3.226 pixels per mm, at a frame rate of 60 Hz. The overall size of the screen was 590 width × 330 mm height. The viewing distance was that used by Michotte, 1.5 m. Observers in his studies were free to move so that feature of the method is retained in the present study, and for that reason spatial measurements are given in millimetres rather than degrees of arc.

General features of stimulus presentations are listed in table 1. Variations from the standard features above are detailed in the method sections of the corresponding experiments.

**Table 1.** Summary of general features of stimulus presentations.

stimuli are presented within a frame with a white ground, 1600 width × 800 pixels height, 496 × 248 mm.
Experiments 1–8 are based on the typical stimulus for launching as illustrated in figure 1; Experiments 9–14 are based on the typical stimulus for entraining.
objects are squares except in Experiment 1 where object width is manipulated and in Experiment 8 which follows Michotte’s experiment 33 in using circular discs.
objects are 12.4 mm on each side except in Experiment 1 where object width is manipulated, Experiment 8 where circular discs with 9.3 mm radius are used and Experiments 3, 11 and 12, where object size is manipulated.
objects move horizontally from left to right except in Experiment 2 where some objects in some stimuli move from right to left.
the object that moves first is black and the object that moves second is red, except in Experiment 1 where both objects are black.
speed of motion is 124 mm s^-1^ except for some stimuli in Experiments 1, 7, 9, 10, 11 and 12 where object speed or speed ratio is manipulated.
object motion continues until the red square exits the frame except for two stimuli in Experiment 2 where objects stop within the frame.
distance moved by each object varies between stimuli and between experiments; the minimum distance used is 124 mm.

It was noted above that, with computer presentations, apparently moving objects actually jump by some number of pixels from one frame to the next. In all cases, stimuli were designed so that exact contact between the two objects occurred; that is, the static frame in which contact occurred showed no gap between and no overlap of the objects.

Table 2 lists the main concern of each experiment and the experiment(s) by Michotte on which each was based. More detailed information is given in the method sections of the individual experiments.

**Table 2.** Summary of replications.

experiment	replication
**launching experiments**	
1	effect of reduced object width (Michotte experiment 10)
2	effect of contextual object motions (Michotte experiments 20, 21, 24–26)
3	effect of object size ([1], p. 82)
4	effect of delay when black square contacts red square (Michotte experiment 29)
5	effect of pause in motion of single object (Michotte experiment 30)
6	effect of non-contact between the two objects (Michotte experiment 31)
7	effect of red square being in motion away from black square before contact (Michotte experiment 17)
8	effect of vertical displacement of black square motion path (Michotte experiment 33)
**entraining experiments**	
9	effect of red square being in motion away from black square before contact (Michotte experiments 48, 49, and 55)
10	effect of relative speed of objects (Michotte experiment 54)
11 and 12	effect of spatial relations between small object and large screen (Michotte experiment 52)
13	effect of delay when black square contacts red square (tested by Michotte for launching but not for entraining)
14	effect of non-contact between the two objects (tested by Michotte for launching but not for entraining)

## 5. Design

Specific experimental designs are described under the individual experiment headings and summarized in table 3. The design plan is in table 4. The 0.01 criterion for statistical significance was used. This was further modified within each experiment by use of the Bonferroni correction based on the number of dependent variables in that experiment. Where appropriate, post hoc paired comparisons were carried out using the Tukey test with the significance level set at 0.05. Effect sizes were calculated using the partial eta squared measure. Significant interactions are not predicted for these studies.

**Table 3.** Experimental designs for all experiments.

experiment	design and analysis
**experiments 1–8: launching stimuli**	
1	I.V. 1. Object width (10 widths in equal increments from 0.62 mm to 6.2 mm).
	I.V. 2. Speed of both objects (62 mm s^-1^ versus 124 mm^-1^ s).
	each statement analysed with two-way ANOVA (within-subjects).
2	five different visual camouflage stimuli. Each analysed separately twice:
	each statement analysed with one-way ANOVA comparison with standard launching stimulus (within-subjects, no fixation condition only).
	each statement analysed with one-way ANOVA for presence versus absence of fixation point (between-subjects).
3	I.V. 1. Size of black square (2.48 mm versus 12.4 mm versus 93 mm).
	I.V. 2. Size of red square (2.48 mm versus 12.4 mm versus 93 mm).
	each statement analysed with two-way ANOVA (within-subjects).
4	I.V. Delay between black square contacting red square and red square moving (13 delays in equal increments from 0 ms to 200 ms).
	each statement analysed with one-way ANOVA (within-subjects).
5	I.V. Pause in motion of single object (13 pause durations in equal increments from 0 ms to 200 ms).
	each statement analysed with one-way ANOVA (within-subjects).
4 and 5	data analysed with one-way ANOVA to assess differences in effects of pause and delay.
6	I.V. 1. Gap size (3.1 mm versus 6.2 mm versus 12.4 mm versus 24.8 mm versus 46.5 mm versus 68.2 mm versus 89.9 mm).
	I.V. 2. Object speed (74.3 mm s^-1^ versus 124.0 mm s^-1^ versus 186.0 mm s^-1^).
	each statement analysed with two-way ANOVA (within-subjects).
7	I.V. 1. Speed ratio of black square before contact to red square after contact (2:1 versus 3:1 versus 4:1 versus 6:1)
	I.V. 2. Speed of red square after contact (18.6 mm s^-1^ versus 37.2 mm s^-1^ versus 74.4 mm s^-1^)
	I.V. 3. Presence v. absence of fixation point (between-subjects).
	each statement analysed with three-way mixed design ANOVA.
8	I.V. Stopping location of black disc with five locations.
	each statement for each stimulus analysed with chi-square test.
**experiments 9–14: entraining stimuli**	
9	I.V. 1. Speed ratio of black square before contact to red square after contact (2:1 versus 3:1 versus 4:1 versus 6:1).
	I.V. 2. Speed of both objects after contact (18.6 mm s^-1^ versus 37.2 mm s^-1^ versus 74.4 mm s^-1^).
	I.V. 3. Presence versus absence of fixation point (between-subjects).
	each statement analysed with three-way mixed design ANOVA.
10	I.V. 1. Speed of black square before contact (62 mm s^-1^ versus 124 mm s^-1^ versus 186 mm s^-1^).
	I.V. 2. Speed of both objects after contact (62 mm s^-1^ versus 124 mm s^-1^ versus 186 mm s^-1^).
	each statement for each stimulus analysed with chi-square test.
11	I.V. 1. Speed of small (red) object (62 mm s^-1^ versus 124 mm s^-1^ versus 186 mm s^-1^).
	I.V. 2. Spatial relations of objects (see table 23 for details).
	each statement analysed with two-way ANOVA (within-subjects).
12	I.V. 1. Speed of large (red) object (62 mm s^-1^ versus 124 mm s^-1^ versus 186 mm s^-1^).
	I.V. 2. Spatial relations of objects (see table 30 for details).
	each statement analysed with two-way ANOVA (within-subjects).
13	I.V. Delay between black square contacting red square and both objects moving (13 delays in equal increments from 0 ms to 200 ms).
	each statement analysed with one-way ANOVA (within-subjects).
14	I.V. 1. Gap size (3.1 mm versus 6.2 mm versus 1 2.4 mm versus 24.8 mm versus 46.5 mm versus 68.2 mm versus 89.9 mm).
	I.V. 2. Object speed (74.3 mm s^-1^ versus 124.0 mm s^-1^ versus 186.0 mm s^-1^).
	each statement analysed with two-way ANOVA (within-subjects).

Note: All experiments have multiple dependent measures (see method sections of individual experiments). Each is analysed separately.

**Table 4.** Design plan.

expt	question	hypothesis	sampling plan	analysis plan	rationale for deciding the sensitivity of the test for confirming or disconfirming the hypothesis	interpretation given different outcomes	theory that could be shown wrong by the outcomes	outcome
1	wil Michotte’s result be replicated?	H1. Passing impression will occur with narrow objects, transition to launching effect with wide objects. Manipulation of speed isexploratory, justified by findings that the launching effect varies with speed.	for *n* = 50, *α* = 0.01, significant *F* ratio for main effect of object width. Post hoc paired comparisons tested with Tukey test with *α* = 0.05. Direct comparisons between statements tested with related means *t-*test. Linear trends on both measures tested with Pearson linear correlation coefficient.	two-way within-subs ANOVA, object width (10 values) × speed of both objects (2 values)	assuming effect size of 0.20 or more, with Power = 0.90and correlation among measures = 0.1, *n* = 39 is adequate. With effect size of .025 or more, *n* = 26 is adequate.	transition from high passing ratings at low width to high launching ratings at high width would be successful replication.^a^ If *F* ratio is significant at 0.01 this will be tested with post hoc paired comparisons (Tukey test). All other results would be failure to replicate. If all launching means were below scale mid-point that would be disconfirmatory for launching effect.	Michotte’s perceptual structure theory.	supported.
2	will Michotte’s results be replicated?	H2. Camouflage manipulations will reduce or eliminate launching effect. This will be qualified by fixation manipulation— see main text for details.	for *n* = 50, *α* = 0.01, significant *F* ratio for main effect of camouflage versus standard. Ditto for main effect of fixation manipulation.	for each stimulus: (a) one-way within subs ANOVA versus standard launching stimulus, no-fixation condition only; (b) one-way between-subs ANOVA for presence absence of fixation point. ANOVA is used for consistency with the other experiments.	assuming effect size of 0.20 or more, with Power = 0.90 and correlation among measures = 0.1, *n* = 62 is adequate. With effect size of 0.25 or more, *n* = 40 is adequate.	significantly higher launching ratings for standard than for camouflage stimuli would be successful replication. All other results would be failure to replicate. Reported effect of fixation were not interpreted by Michotte; unexpected interactions will not be interpreted but will be discussed in the discussion.	module hypothesis.	partly supported.
3	will manipulating object size affect ratings of launching?	H3. The launching effect will not be affected by object size manipulation (as claimed by Michotte).	for *n* = 50, *α* = 0.01, significant *F* ratio for main effect of delay. Null hypothesis tested with Bayesian linear mixed model.	two-way within-subs, size of black square (3 values) × size of red square (3 values).	assuming effect size of 0.20 or more, with Power = 0.90 and correlation among measures = 0.1, *n* = 66 is adequate. With effect size of 0.25 or more, *n* = 42 is adequate.	significant effect of size of either object on launching ratings would disconfirm Michotte’s claim. Non-significant effect would be consistent with it.	significant effect is not predicted by Michotte’s theory but would not be disconfirmatory for it.	partly supported.
4	will Michotte’s result be replicated?	H4. Launching ratings will decline as delay increases.	for *n* = 50, *α* = 0.01, significant *F* ratio for main effect of delay. Post hoc single contrast testing for linear trend with *α* = 0.05.	one-way within-subs ANOVA, delay duration, (13 values).	assuming effect size of .20 or more, with Power = 0.90 and correlation among measures = 0.1, *n* = 51 is adequate. With effect size of 0.25 or more, *n* = 33 is adequate.	statistically significant decline of launching ratings with increasing delay would be successful replication. All other results would be failureto replicate. If *F* ratio is significant at .01 this will be tested with post hoc paired comparisons (Tukey).	Michotte’s perceptual structure theory and module hypothesis.	partly supported.
5	will Michotte’s result be replicated?	H5. Ratings of continuous motion will decline as pause duration increases. H6. There will be a high positive correlation with launching ratings in Experiment 4.	for *n* = 50, *α* = 0.01, significant *F* ratio for main effect of pause duration. Post hoc single contrast testing for linear trend with *α* = 0.05.	one-way within-subs ANOVA, pause duration, (13 values).	assuming effect size of .20 or more, with Power = 0.90 and correlation among measures = 0.1, *n* = 51 is adequate. With effect size of 0.25 or more, *n* = 33 is adequate.	statistically significant decline of continuous motion ratings with increasing pause duration would be successful replication, as would significant and high positive correlation with launching ratings in Experiment 4; low or negative correlation would be failure to replicate. If *F* ratio is significant at 0.01 this will be tested with post hoc paired comparisons (Tukey).	Michotte’s perceptual structure theory.	H5 partly supported. H6 not supported.
6	will Michotte’s result be replicated?	H7. Ratings of launching will decline as gap size increases. H8. Ratings of launching will increase as speed increases.	for *n* = 50, *α* = 0.01, significant *F* ratio for main effect of gap size. Post hoc single contrast testing for linear trend with *α* = 0.05. Main effect of speed, post hoc testing with Tukey test.	two-way within-subs ANOVA, gap size (7 values) × object speed (3 values).	assuming effect size of 0.20 or more, with Power = 0.90 and correlation among measures = 0.1, *n* = 38 is adequate. With effect size of 0.25 or more, *n* = 26 is adequate.	decline of launching ratings with increasing gap size would be successful replication. All other results for gap size would be failure to replicate. Increase of launching ratings with increasing object speed would be successful replication. All other results for object speed would be failure to replicate.	Michotte’s perceptual structure theory and module hypothesis.	H7 supported. H8 supported.
7	will Michotte’s result be replicated?	H9. Ratings of launching will be above scale mid-point for all stimuli. H10. That will be qualified by fixation manipulation. See main text for details.	for *n* = 50, *α* = 0.01, significant *t-*tests for comparison between ratings and scale mid-point.	*t*-tests comparing ratings for each stimulus with scale mid-point.	assuming effect size of .20 or more, with Power = 0.90 and correlation among measures = 0.1, *n* = 36 is adequate. With effect size of 0.25 or more, *n* = 24 is adequate.	launching ratings above scale mid-point for all stimuli would be successful replication. Significant effects would not be disconfirmatory unless one or more means was below scale mid-point. Reported effects of fixation were not interpreted by Michotte; interpretation here will depend on results.	Michotte’s perceptual structure theory.	H9 not supported. H10 not supported.
8	will Michotte’s result be replicated?	H11. Launching effect will be weak or absent for all stimuli.	non-significant chi-square test on comparisons between statements for each stimulus. or significant similarity.	comparisons between statements for each stimulus using chi- square test.	nonparametric analysis that allows possibility of testing significant similarity will be used.	non-significant result with means below scale mid-point on launching measure would be successful replication. Significant result with at least one mean above scale mid-point would be unpredicted; interpretation would depend on details of result.	Michotte’s perceptual structure theory.	supported.
9	will Michotte’s result be replicated?	H12. If black square is fixated, entraining effect will occur for all stimuli. If red square is fixated, entraining effect will not occur.	For *n* = 50, *α* = 0.01, significant *t*-tests for comparison between ratings and scale mid-point.	*t*-tests comparing ratings for each stimulus with scale mid-point.	assuming effect size of 0.20 or more, with Power = 0.90 and correlation among measures = 0.1, *n* = 36 is adequate. With effect size of 0.25 or more, *n* = 24 is adequate.	with fixation on black square, entraining ratings above scale mid-point for all stimuli would be successful replication. Significant effects would not be disconfirmatory unless one or more means was below scale mid-point. Reported effects of fixation were not interpreted by Michotte; interpretation here will depend on results.	Michotte’s perceptual structure theory.	not supported.
10	will Michotte’s result be replicated?	H13. Entraining effect will occur for all stimuli.	non-significant chi-square test on comparisons between statements for each stimulus. or significant similarity.	comparisons between statements for each stimulus using chi-square test.	nonparametric analysis that allows possibility of testing significant similarity will be used. .	entraining ratings above scale mid-point for all stimuli would be successful replication. Significant effects would not be disconfirmatory unless one or more means was below scale mid-point.	Michotte’s perceptual structure theory.	not supported.
11	will qualitative impression change depending on spatial relations between stimuli?	H14. When both objects have the same speed, there will be qualitative differences in reported impressions with launching favoured for some stimuli, entraining for others, and pulling for others. When the objects have different speeds, differences will be weak or absent.^b^	For *n* = 50, *α* = 0.01, significant *F* ratio for main effects of spatial relation and speed of small object. Post hoc paired comparisons tested with Tukey test with *α* = 0.05.	two-way within-subs ANOVA, spatial relation between objects when both are in motion (7 values) × speed of small object (3 values). Comparisons between statements analysed with one-way ANOVA with repeated measures.	assuming effect size of 0.20 or more, with Power = 0.90 and correlation among measures = 0.1, *n* = 38 is adequate. With effect size of 0.25 or more, *n* = 24 is adequate.	significant differences between measures can be interpreted as qualitative differences in perceptual impression. For example, if pulling ratings are significantly higher than launching, entraining, and independent motion ratings for a particular stimulus, that would support interpretation that a pulling impression occurs. See main text for more details.	Michotte’s perceptual structure theory.	supported.
12	will qualitative impression change depending on spatial relations between stimuli?	H15. When both objects have the same speed, there will be qualitative differences in reported impressions with launching favoured for some stimuli, entraining for others, and pulling for others. When the objects have different speeds, differences will be weak or absent.^b^	For *n* = 50, *α* = 0.01, significant *F* ratio for main effects of spatial relation and speed of large object. Post hoc paired comparisons tested with Tukey test with *α* = 0.05	two-way within-subs ANOVA, spatial relation between objects when both are in motion (7 values) × speed of small object (3 values). Comparisons between statements analysed with one-way ANOVA with repeated measures.	assuming effect size of .20 or more, with Power = 0.90 and correlation among measures = 0.1, *n* = 38 is adequate. With effect size of 0.25 or more, *n* = 24 is adequate.	significant differences between measures can be interpreted as qualitative differences in perceptual impression. For example, if pulling ratings are significantly higher than launching, entraining, and independent motion ratings for a particular stimulus, that would support interpretation that a pulling impression occurs. See main text for more details.	Michotte’s perceptual structure theory.	supported.
13	will effect of delay for entraining be similar to that for launching?	H16. Entraining effect will decline as delay increases.	For *n* = 50, *α* = 0.01, significant *F* ratio for main effect of delay. Post hoc single contrast testing for linear trend with *α* = 0.05.	one-way within-subs ANOVA, delay duration (13 values).	assuming effect size of 0.20 or more, with Power = 0.90 and correlation among measures = 0.1, *n* = 51 is adequate. With effect size of 0.25 or more, *n* = 33 is adequate.	statistically significant decline of entraining ratings with increasing delay would support hypothesis. All other results would fail to support hypothesis. If *F* ratio is significant at 0.01 this will be tested with post hoc paired comparisons (Tukey test with *α* = 0.05.	Michotte’s perceptual structure theory.	partly supported.
14	will effect of gap size for entraining be similar to that for launching?	H17. Entraining effect will decline as gap size increases. H18. Entraining effect will increase as speed increases.	For *n* = 50, *α* = 0.01, significant *F* ratio for main effect of gap size. Post hoc single contrast testing for linear trend with *α* = 0.05. Main effect of speed tested with Tukey test.	two-way within-subs ANOVA, gap size (7 values) × object speed (3 values).	assuming effect size of .20 or more, with Power = 0.90 and correlation among measures = 0.1, *n* = 38 is adequate. With effect size of 0.25 or more, *n* = 26 is adequate.	statistically significant decline of entraining ratings with increasing gap size would support hypothesis. All other results would be failure to support hypothesis. If *F* ratio is significant at 0.01 this will be tested with post hoc paired comparisons (Tukey test).	Michotte’s perceptual structure theory.	H17 partly supported. H18 not supported.

^a^ Available evidence ([1], experiment 10) indicates only that the transition from passing to launching should occur somewhere between 1 and 5 mm object width.

^b^ Michotte [1] tested only a single stimulus with both objects moving at the same speed (experiment 52) and found entraining. Experiments 11 and 12 extend this by manipulating spatial relations between the objects when both are in motion, so occurrence of qualitative differences between them in causal impressions is a novel prediction.

Michotte did not test hypotheses, nor did he carry out statistical analysis. His reports of results were all consistent with the perceptual structure hypothesis that was developed as the research went on. So the overall plan is to test the general hypothesis that the results and observations reported by Michotte are reliable. Failures to replicate, which would include non-significant results and significant results in the direction opposite to that reported by Michotte, would have disconfirmatory value for his perceptual structure theory. More detail about that, the experimental designs, and the rationale for the designs can be found in the main text under the individual experiment headings. None of the hypotheses predicts a significant interaction, so these are not mentioned in the design plan. Desired sample sizes were calculated using G*Power 3.1.

## 6. Procedure

The experiments were run in a small windowless laboratory with fluorescent lighting giving a moderate ambient light level. Each experiment had its own written instructions, including the dependent measures for the respective experiments (see https://osf.io/5dygp for details), and the experimenter checked that the participant understood the instructions each time. When the participant indicated that they understood the instructions, the experimenter presented the stimuli one at a time and the participant responded to each one by filling out the rating scales provided. Order of experiments was randomized independently for each participant and order of stimuli within experiments was similarly randomized. In each experiment, each stimulus was presented once to each participant. Given the large total number of stimuli, participants were permitted to take short breaks between experiments.

Initially, a series of six stimuli chosen from the experiments and including typical stimuli for the launching and entraining effects were presented in random order. Before these were presented, participants were instructed that the experiments were concerned with their impressions of what they see, not with any thoughts they might have about the stimuli, and that the series of stimuli was to give them an idea of the kinds of stimuli that would be encountered in the experiments. They were instructed to observe the stimuli and that no response was required, and they were invited to ask questions if they had any. No participants asked any questions. There were four experimenters, two for each group of experiments, and each ran 25 participants. The experimenters were naive to the aims and hypotheses.

## 7. Experiment 1: object width

Experiment 1 is based on experiment 10 in [1, p. 49]. A single stimulus was presented in which the width of the objects was 1 mm (compared with 5 mm in the standard stimulus). Michotte reported that the launching effect did not occur. Instead there was an impression that he termed the Tunnel Effect, which is an impression of one object passing over or behind another. Impressions of one object passing over another object have been reported in several experiments by Scholl & colleagues [29,37,48,61]. In those experiments, the object that moved first stopped at a point where it partly or completely occluded the other object, and various manipulated factors influenced whether the first object was perceived as launching the other object or as passing over it. Michotte’s experiment 10 was different in that the passing impression was reported when there was no overlap of the objects, and it has not previously been replicated.

Effects of object speed on the launching effect have often been reported, as was discussed earlier, so it is possible that the point of transition from passing to launching might vary depending on speed. For that reason, object speed was also manipulated.

H1. There should be linear trends for ratings of non-causal passing to decrease and for launching ratings to increase with increasing width. Non-causal passing ratings should be significantly higher than launching ratings at the narrowest width, and launching ratings should be higher than passing ratings at the greatest width. There is no basis for predicting exactly where the transition from passing to launching will occur except that it should be at less than 5 mm. No significant interaction with object speed is predicted.

### 7.1. Method

Michotte did not report any variations on the stimulus in experiment 10. Experiment 1 is therefore an extended replication. Stimuli were based on the launching effect stimulus depicted in figure 1. Object width (of both objects) was varied from 0.62 to 6.2 mm in increments of 0.62 mm (2 pixels), resulting in 10 different widths. The height of the objects was 12.4 mm in all stimuli. Speed was manipulated with two values, 124 and 62 mm s^-1^, with both objects moving at the same speed in any given stimulus. Both objects were the same colour (black) so that colour difference could not be used as a cue to interpret what happened.

Written instructions to participants began as follows: ‘In this experiment you will see a series of short movies, about one or two seconds in duration, each involving two objects, both black rectangles. Each movie will begin with one rectangle moving towards the other. We are interested in what you see when the moving rectangle reaches the other one, the visual impression you have of the movies, not any thoughts you might have about what you are seeing. For each movie you will be asked to rate the extent to which you agree or disagree with each of three statements as descriptions of your visual impression of what happened. The three statements are as follows:

The initially moving rectangle made the other rectangle move by bumping into it.

The initially moving rectangle passed across the other rectangle, which moved little or not at all.

The initially stationary rectangle moved off when the moving one reached it, but it moved independently and its motion was not caused by the other rectangle.’

The statement for passing is based on Michotte’s description of the Tunnel Effect. The statement for independent motion is also based on Michotte’s preferred form of expression—the term ‘independent(ly)’ was used frequently in Michotte [1]—in described impressions of stimuli in which the launching effect did not occur.

### 7.2. Results

For each measure, data were initially analysed with a 2 (speed; 62 versus 124 mm s^-1^) × 10 (object width, 0.62 versus 1.24 versus 1.86 versus 2.48 versus 3.10 versus 3.72 versus 4.34 versus 4.96 versus 5.58 versus 6.20 mm) within-subjects analysis of variance (ANOVA).

#### 7.2.1. Launching measure

**Figure 2.**
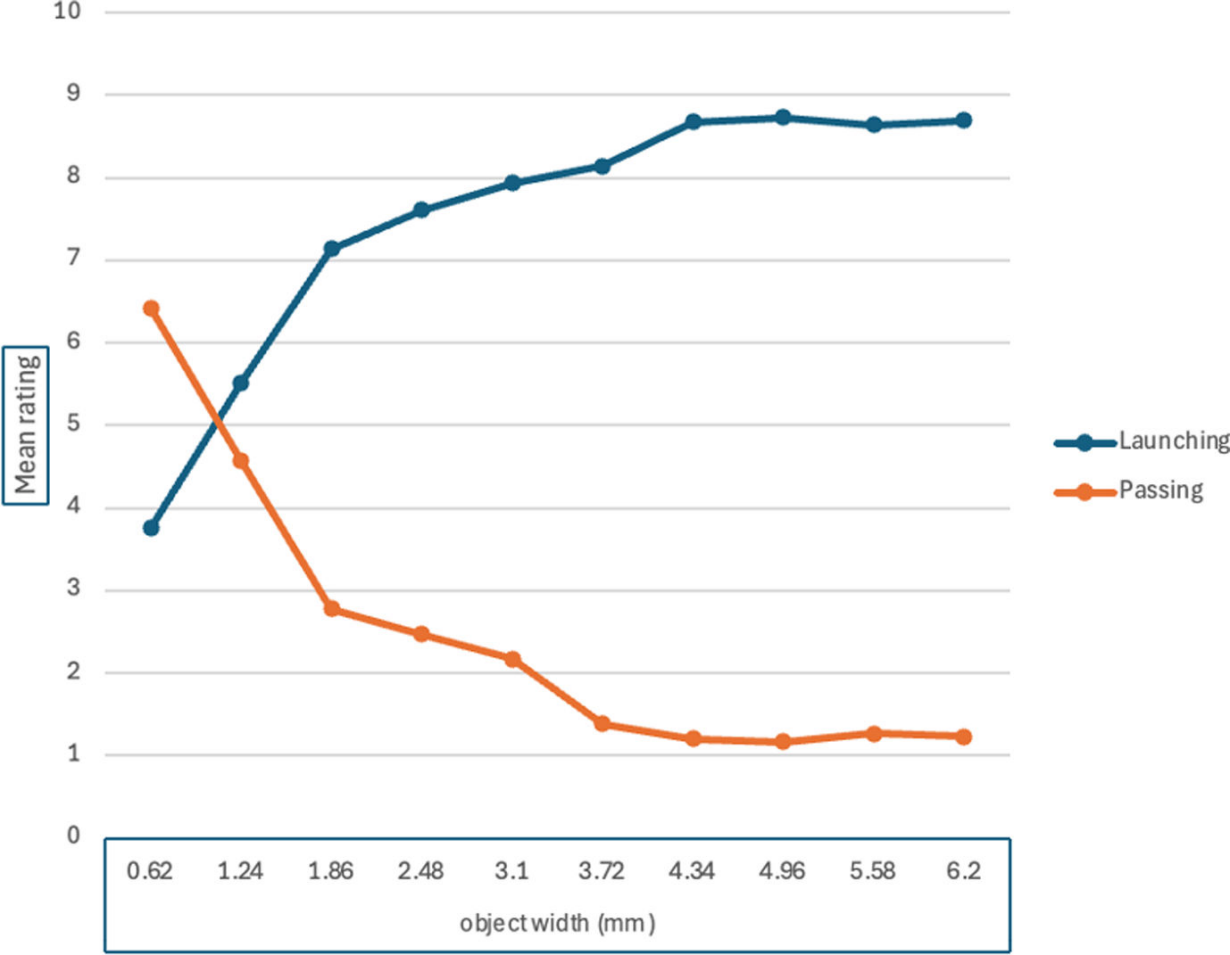
Mean ratings on launching and passing measures with varying object width, Experiment 1.

There was a significant effect of object width, *F* (9, 441) = 38.74, m.s.e. = 6.94, *p* < 0.001, η_p_^2^ = .44. Means are reported in table 5 and illustrated in figure 2. Post hoc paired comparisons with the Tukey test revealed that the mean for 0.62 mm was significantly lower than all others; the mean for 1.24 mm was significantly lower than all the remainder; and the mean for 1.86 mm was significantly lower than the means for the four largest widths. As table 5 shows, there was a rapid initial increase in ratings with increasing width, reaching a plateau around 3.10 mm. The main effect of speed was not significant, *F* (1, 49) = 0.09, m.s.e. = 7.28, *p* = 0.76, η_p_^2^ = 0.002. The interaction between speed and object width was not significant, *F* (9, 441) = 1.09, m.s.e. = 4.82, *p* = 0.36, η_p_^2^ = 0.02.

**Table 5.** Mean ratings, Experiment 1.

	measure		
object width (mm)	launching	passing	independent
0.62	3.75	6.42	1.71
1.24	5.52	4.57	1.67
1.86	7.14	2.77	2
2.48	7.61	2.47	2.21
3.1	7.93	2.17	1.84
3.72	8.14	1.38	2.07
4.34	8.67	1.2	2.02
4.96	8.73	1.16	1.73
5.58	8.64	1.26	1.79
6.2	8.69	1.23	1.91

#### 7.2.2. Passing measure

There was a significant effect of object width, *F* (9, 441) = 39.97, m.s.e. = 7.70, *p* < 0.001, η_p_^2^ = 0.44. Means are reported in table 5 and illustrated in figure 2. Post hoc paired comparisons with the Tukey test revealed that the mean for 0.62 mm was significantly higher than all others; the mean for 1.24 mm was significantly higher than all the remainder; and the mean for 1.86 mm was significantly higher than the means for the four largest widths. As table 5 shows, there was a rapid initial decline in ratings with increasing width, reaching a plateau around 3.10 mm. This is a close mirror image of the results on the launching measure. The main effect of speed was not significant, *F* (1, 49) = 0.25, m.s.e. = 9.54, *p* = 0.62, η_p_^2^ = 0.005. The interaction between speed and object width was not significant, *F* (9, 441) = 2.04, m.s.e. = 5.32, *p* = 0.03, η_p_^2^ = 0.04.

#### 7.2.3. Independent motion measure

There were no significant effects and, as table 5 shows, means were uniformly close to the lower end of the scale. For speed, *F* (1, 49) = 1.10, m.s.e. = 5.39, *p* = 0.30, η_p_^2^ = 0.02. For object width, *F* (9, 441) = 0.85, m.s.e. = 3.69, *p* = 0.57, η_p_^2^ = 0.02. For the interaction, *F* (9, 441) = 0.70, m.s.e. = 2.90, *p* = 0.70, η_p_^2^ = 0.01.

#### 7.2.4. Paired comparisons between measures

For each movie, one-way ANOVA was carried out comparing ratings on the three measures. Results are reported in table 6. The table shows that the passing measure received significantly higher ratings than both other measures only at 0.62 mm object width (at both speeds—videos 1 and 11). For almost all movies, launching was the dominant impression.

**Table 6.** Paired comparisons between measures, Experiment 1.

movie no.	object width (mm)	*F*	m.s.e.	*p*	*η*_p_^2^	differences
1	0.62	13.37	18.25	<0.001	0.54	P > L & I
2	1.24	11.41	18.74	<0.001	0.32	L & P > I
3	1.86	26.12	12.44	<0.001	0.52	L & P > I
4	2.48	22.79	13.82	<0.001	0.48	L > P & I
5	3.1	48.25	11.72	<0.001	0.66	L > P & I
6	3.72	118.72	6.47	<0.001	0.82	L > P & I
7	4.34	144.58	6.39	<0.001	0.86	L > P & I
8	4.96	166.84	5.5	<0.001	0.87	L > P & I
9	5.58	154.62	5.4	<0.001	0.86	L > P & I
10	6.2	168.78	5.24	<0.001	0.87	L > P & I
11	0.62	17.51	18.06	<0.001	0.42	P > L & I
12	1.24	11.08	17.91	<0.001	0.45	L & P > I
13	1.86	40.35	11.52	<0.001	0.62	L > P & I
14	2.48	67.37	9.11	<0.001	0.73	L > P & I
15	3.1	61.89	9.86	<0.001	0.72	L > P & I
16	3.72	59.73	10.38	<0.001	0.71	L > P & I
17	4.34	112.69	6.74	<0.001	0.82	L > P & I
18	4.96	141.21	6.09	<0.001	0.85	L > P & I
19	5.58	138.34	6.21	<0.001	0.85	L > P & I
20	6.2	115.93	7.06	<0.001	0.83	L > P & I

Note. L = Launching measure; P = Passing measure; I = Independent motion measure. Movies 1–10 were at speed 124 mm s^-1^; Movies 11–20 were at speed 62 mm s^-1^. d.f. = 2, 98.

### 7.3. Summary of results and discussion

Michotte [1] reported that the launching effect did not occur if the objects were 1 mm wide. The results of the present study are consistent with that: ratings were significantly higher on the passing measure than on the launching measure at the narrowest width of 0.62 mm. There was no significant difference between launching and passing at 1.24 and 1.86 mm; at all greater widths, launching was rated significantly higher than passing. Ratings on the independent motion measure were consistently low, never higher than 2.07. Object speed had no significant effect. Results were, therefore, consistent with H1, with a decreasing trend on the passing measure and an increasing trend on the launching measure.

One possible explanation for the results concerns the technology used. The stimuli are frames presented at 60 Hz. The spatial location of the moving object jumps abruptly from one frame to the next. The movies were designed so that there was actual contact (adjacency without overlap) between the objects in one frame, but the jump in location from one frame to the next is greater than the width of the narrowest object used. The impression of motion is constructed by some form of integration over successive frames of the stimulus. Therefore the passing impression could occur because the integration mechanism is not sensitive to the very tiny offset between the two objects at contact and therefore does not detect that the initially stationary object is now jumping across the screen. This possibility cannot be ruled out and is worthy of further investigation. Michotte’s stimuli presented genuinely continuous (if equally illusory) motion and that might make discontinuities in motion more easily detectable but, if that were the case, the passing impression should not have occurred with Michotte’s stimuli. One problem for the technology-based hypothesis is that the gap between successive locations of the moving object is twice as great at the higher speed as what it is at the lower speed. Despite that, object speed had no significant effect on any of the three measures. That would suggest that issues to do with integrating over spatially discontinuous presentations of the moving object do not suffice to explain the occurrence of the passing impression.

A second possible explanation concerns visual acuity. This is a complex topic and there is space only for a brief glance at it here. With moving object stimuli the kind of acuity that is relevant is dynamic visual acuity (DVA), visual acuity for moving targets [62]. A key feature for present purposes is that speeds used were quite slow compared with those used in much research on DVA: for example Ludvigh & Miller [63] used target velocities up to 180° s^−1^, whereas stimulus presentations here would have covered only a few degrees of arc, depending on the participant’s distance from the screen, and the motion continued for more than 1000 ms even at the higher speed. Under those conditions research has shown that DVA even for briefly presented targets is scarcely worse than that for stationary targets, which is about 1 min of arc [64–67]. Given that, the two objects should be easily discriminable even at the minimum width of 0.62 mm, so it is likely that any effect of limited DVA is minimal with these stimuli. Object width of 1 mm, therefore, appears to be a genuine limit on conditions for occurrence of the launching effect.

## 8. Experiment 2: camouflage

Experiments 20–26 were called camouflage experiments by Michotte [1]. The basic principle was to present a typical stimulus for launching but in a context of other movements, of one or both of the two objects themselves or of additional objects. In experiments 22 and 23 one of the objects changed shape without otherwise moving. Experiment 2 is a replication of the other five experiments (20, 21, 24–26).

**Figure 3.**
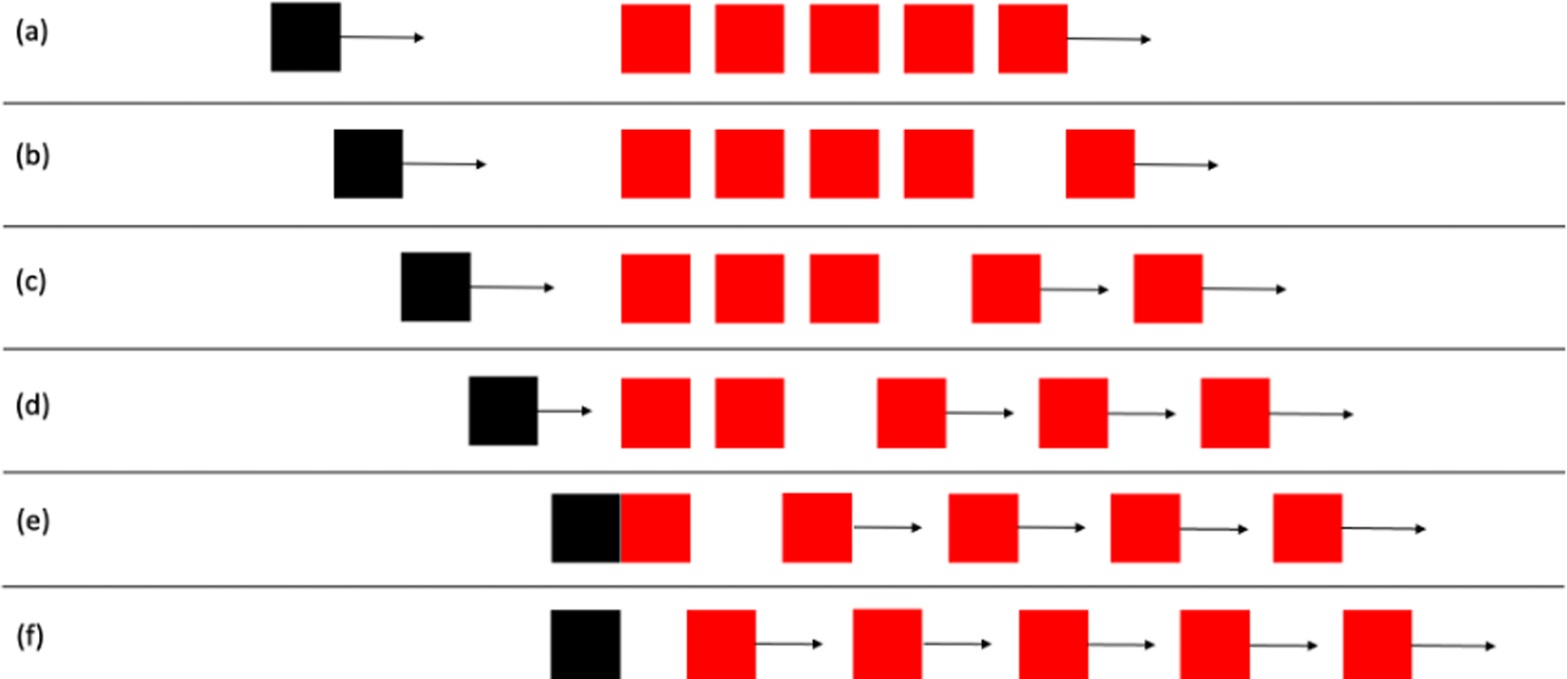
Schematic representation of camouflage stimulus in Experiment 2, based on [1], experiment 20. (a) Shows the first frame of the stimulus: the black square starts to move and the rightmost red square also starts to move with the same velocity. (b) Shows these object motions continuing. In (c) the next red square has also started to move with the same velocity. (d) Shows the next red square moving in the same way. (e) Shows the frame in which the black square contacts the leftmost red square. At that point, the fourth red square has also started to move, and the black square stops. (f) Then shows the leftmost red square moving off as in the standard stimulus for the launching effect (figure 1). Equal amounts of time elapse between successive onsets of motion in the red squares.

In experiment 20, the red square was the leftmost of a series of five red squares with gaps of 1.5 mm between them. Figure 3 depicts the sequence of events in this stimulus. When the black square begins to move, the rightmost of the red squares starts moving to the right. Each one in turn starts moving with the same velocity at regular intervals, timed so that the leftmost one starts to move when the black square contacts it. The red squares continue to move until they have exited the frame. Thus, it is a standard launching stimulus, but with a visible context of other moving objects. Michotte [1] reported that the launching effect did not occur with this stimulus, unless the point of contact between the black square and the leftmost red square was fixated.

In experiment 21, when the black square started moving, the red square moved to the right then back to its starting position and repeated this, with the motion timed so that it reached its starting position just as the black square arrived there. Apart from that the stimulus was a standard launching stimulus. Michotte reported that the launching effect did not occur ‘when observers look at the situation as a whole’ [1, p. 74] but that it did occur when the contact point was fixated.

**Figure 4.**
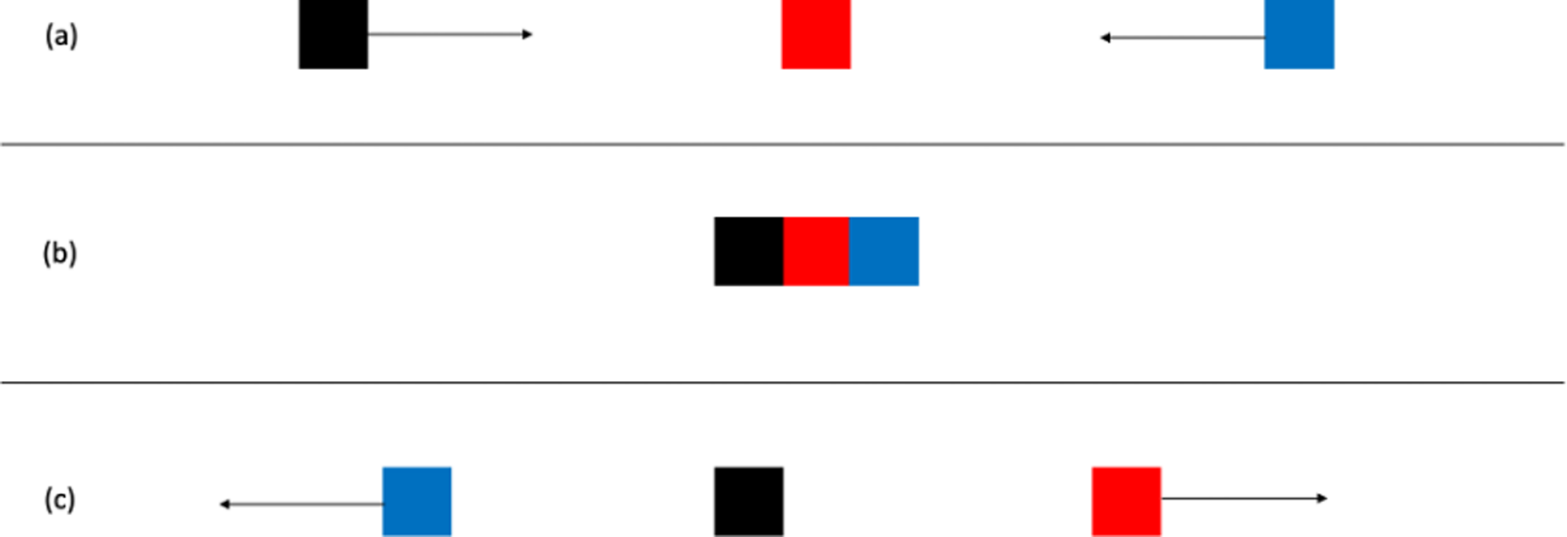
Schematic representation of camouflage stimulus in Experiment 2, based on [1], experiment 24. (a) Shows the first frame of the stimulus with motion directions indicated for the black square and the blue square. (b) Shows the frame in which the black square and the blue square contact the red square. At that point, the black square stops and the red square moves off as in the standard stimulus for the launching effect. The blue square continues to move to the left, passing behind the black and red squares so that the black and red squares were not occluded. (c) Shows the continuing motion of the red and blue squares.

In experiment 24, a third object was added. In the present experiment, this object is coloured blue to distinguish it from the other two objects. This object started to the right of the red square and moved towards it, timed so that contact with the red square coincided with contact of the black square with the red square. The third object then continued to move to the left. The motion sequence is schematically depicted in figure 4.

Experiment 25 was similar to the typical stimulus for launching except that, on contacting the red square, the black square returned to its starting point at the same speed. Michotte reported that the launching effect did not occur.

In experiment 26, the red square was initially located further to the right than usual. Both objects started moving towards each other simultaneously. When they came into contact, the black square stopped and the red square moved to the right as in the typical launching stimulus. Michotte reported a strong launching effect with this stimulus.

These experiments are potentially important to any theoretical account of perceptual impressions of causality because the typical stimulus for launching is there in all of them, but, with the exception of experiment 26, the launching effect was reported not to occur. It is important to understand why the launching effect is eliminated by the presence and movement of other objects, if the replication confirms that result.

H2. Camouflage manipulations, with the exception of the stimulus based on experiment 26, will reduce or eliminate the launching effect. This will be qualified by effects of fixation similar to those reported by Michotte [1].

### 8.1. Method

Stimuli matching the descriptions of those used by Michotte and summarized above were constructed. In experiments 20 and 21, Michotte [1] commented that the launching effect did occur if the point of contact between the black square and the red square was fixated. For this reason, for all of the stimuli a fixation point, a small black cross, was located adjacent to the point of contact, and presence versus absence of fixation was manipulated between subjects with 25 participants in each condition.

It is not easy to prepare instructions for participants in the no-fixation condition that do not carry an implicit demand for them to fixate on the contact point: they are, after all, reporting on their perception of what happens at contact. The instructions for the condition without the fixation point therefore drew on the language used by Michotte, as quoted above, and asked participants to look at the movie and the objects in it as a whole. They were also told that, at some point during the movie, a black square would contact a red square and the red square would move away. The two statements with which participants rated agreement or disagreement were as follows:

The black square made the red square move by bumping into it.

The red square moved when the black square reached it, but it moved independently and its motion was not caused by the black square.

To test for camouflage effects, data for each stimulus were compared with data from a standard launching stimulus (the 12.4 × 12.4 mm size condition from Experiment 3) to assess whether the launching effect is significantly reduced by the camouflage manipulation.

### 8.2. Results

For each stimulus, data on each measure were analysed with a 2 between (fixation versus no fixation) × 2 within (camouflage stimulus versus standard launching stimulus) mixed design ANOVA.

#### 8.2.1. Stimulus 1

The basic movie for this is the one depicted in figure 3 and based on Michotte’s experiment 30. There was a significant effect of stimulus, *F* (1, 48) = 111.88, m.s.e. = 7.36, *p* < 0.001, η_p_^2^ = 0.70, with a higher mean for the standard launching stimulus. Means are shown in table 7. There was no significant effect of fixation, *F* (1, 48) = 3.23, m.s.e. = 5.72, ns, η_p_^2^ = 0.06. The interaction was not significant, *F* (1, 48) = 0.60, m.s.e. = 7.36, ns, η_p_^2^ = 0.01.

**Table 7.** Mean ratings, Experiment 2.

	measure	
stimulus	launching	independent
Standard	8.62	1.6
1 (experiment 20)	2.88	7.26
2 (experiment 21)	3.84	6.3
3 (experiment 24)	4.2	6.02
4 (experiment 25)	7.9	2.78
5 (experiment 26)	8.14	2.44

On the independent motion measure there was a significant effect of stimulus, *F* (1, 48) = 104.63, m.s.e. = 7.67, *p* < 0.001, η_p_^2^ = 0.69. As the means in table 7 show, there was a high mean for the camouflage stimulus and a low one for the standard launching stimulus. The effect of fixation was not significant, *F* (1, 48) = 4.26, m.s.e. = 6.17, *p* = 0.04, η_p_^2^ = 0.08. The interaction was not significant, *F* (1, 48) = 1.85, m.s.e. = 7.67, *p* = 0.18, η_p_^2^ = 0.04.

#### 8.2.2. Stimulus 2

The camouflage movie here is the one based on Michotte’s experiment 21 with repeated back-and-forth motion of the red square. On the launching measure there was a significant effect of stimulus, *F* (1, 48) = 91.69, m.s.e. = 6.23, *p* < 0.001, η_p_^2^ = 0.66, with a higher mean for the standard launching stimulus. Means are shown in table 7. There was no significant effect of fixation, *F* (1, 48) = 0.01, m.s.e. = 8.06, *p* = 0.92, η_p_^2^ = 0.00. The interaction was not significant, *F* (1, 48) = 1.00, m.s.e. = 6.23, *p* = 0.32, η_p_^2^ = 0.02.

On the independent motion measure there was a significant effect of stimulus, *F* (1, 48) = 81.65, m.s.e. = 6.76, *p* < 0.001, η_p_^2^ = 0.63. Here too, table 7 shows a high mean for the camouflage stimulus and a low one for the standard launching stimulus. The effect of fixation was not significant, *F* (1, 48) = 0.01, m.s.e. = 8.34, *p* = 0.92, η_p_^2^ = 0.00. The interaction was not significant, *F* (1, 48) = 0.53, m.s.e. = 6.76, *p* = 0.47, η_p_^2^ = 0.01.

#### 8.2.3. Stimulus 3

This was based on Michotte’s experiment 24 in which a third object, a blue square moving from right to left, was added to the standard launching stimulus, as shown in figure 4. There was a significant effect of stimulus, *F* (1, 48) = 74.57, m.s.e. = 6.55, *p* < 0.001, η_p_^2^ = 0.61, with a higher mean for the standard launching stimulus. Means are shown in table 7. There was no significant effect of fixation, *F* (1, 48) = 0.27, m.s.e. = 8.32, *p* = 0.61, η_p_^2^ = 0.01. The interaction was not significant, *F* (1, 48) = 2.09, m.s.e. = 6.55, *p* = 0.15, η_p_^2^ = 0.04.

On the independent motion measure there was a significant effect of stimulus, *F* (1, 48) = 59.99, m.s.e. = 8.14, *p* < 0.001, η_p_^2^ = 0.56. Here too, table 7 shows a high mean for the camouflage stimulus and a low one for the standard launching stimulus. The effect of fixation was not significant, *F* (1, 48) = 0.17, m.s.e. = 7.20, *p* = 0.68, η_p_^2^ = 0.00. The interaction was not significant, *F* (1, 48) = 0.89, m.s.e. = 8.14, *p* = 0.35, η_p_^2^ = 0.02.

#### 8.2.4. Stimulus 4

This was based on Michotte’s experiment 25 in which the black square returned to its starting point after contacting the red square. On the launching measure, there were no significant effects. For fixation, *F* (1, 48) = 0.54, m.s.e. = 6.04, *p* = 0.47, η_p_^2^ = 0.01. For stimulus, *F* (1, 48) = 2.30, m.s.e. = 5.64, *p* = 0.14, η_p_^2^ = 0.05. For the interaction, *F* (1, 48) = 0.03, m.s.e. = 5.64, *p* = 0.87, η_p_^2^ = 0.001. Means are shown in table 7. The manipulation of the black square’s motion after contact therefore had no significant effect on reports of the launching effect, contrary to what Michotte [1] reported.

There were no significant effects on the independent motion measure. For fixation, *F* (1, 48) = 0.25, m.s.e. = 6.80, *p* = 0.62, η_p_^2^ = 0.005. For stimulus, *F* (1, 48) = 5.24, m.s.e. = 6.64, *p* = 0.03, η_p_^2^ = 0.10. For the interaction, *F* (1, 48) = 0.01, m.s.e. = 6.64, *p* = 0.91, η_p_^2^ = 0.00.

#### 8.2.5. Stimulus 5

This was based on Michotte’s experiment 26 in which the two squares initially moved towards each other. On the launching measure there were no significant effects. For fixation, *F* (1, 48) = 0.00, m.s.e. = 5.76, *p* = 1.00, η_p_^2^ = 0.00. For stimulus, *F* (1, 48) = 1.64, m.s.e. = 3.51, *p* = 0.21, η_p_^2^ = 0.03. For the interaction, *F* (1, 48) = 1.38, m.s.e. = 3.51, *p* = 0.25, η_p_^2^ = 0.03. Means are shown in table 7. This appears to be consistent with what Michotte [1] reported, although there is no evidence that the launching effect was any stronger with this stimulus than with the standard launching stimulus.

There were no significant effects on the independent motion measure. For fixation, *F* (1, 48) = 0.00, m.s.e. = 7.29, *p* = 1.00, η_p_^2^ = 0.00. For stimulus, *F* (1, 48) = 3.72, m.s.e. = 4.75, *p* = 0.06, η_p_^2^ = 0.07. For the interaction, *F* (1, 48) = 0.54, m.s.e. = 4.75, *p* = 0.47, η_p_^2^ = 0.01.

### 8.3. Discussion

Results for stimuli 1, 2 and 3 confirmed Michotte’s observation that the launching effect is minimal or absent when the standard stimulus is presented with additional movements: making the red square one of a group of objects exhibiting successive and similar motion, making the red square move back and forth before the black square contacts it, and having a third object, a blue square, crossing from right to left. For stimulus 4, in which the black square moved back to its starting point after contacting the red square, there was no significant diminution of the launching effect, contrary to what Michotte [1] reported. Finally, having the red square move right to left before contact did not significantly diminish the launching effect, consistent with what Michotte [1] reported.

There was no significant effect of or interaction with fixation for any stimulus, contrary to Michotte’s [1] observations, so in this respect H2 was not supported. There are several possible explanations for this. One possibility is that participants in the no-fixation condition might spontaneously fixate the stimulus in the same way as those in the fixation condition were instructed to do. This seems unlikely because it is natural to track the moving object with a smooth pursuit eye movement; on the other hand, the camouflage manipulations introduce additional motions, meaning that a decision has to be made about which object to track. Manipulating instructions for fixation would be necessary to test this possibility. A second possibility is that participants in the fixation condition did not maintain gaze as they were instructed to do. The experimenter monitored the participants during stimulus presentation and reported that they appeared to be maintaining fixation, but it is impossible to be certain of that without using an eye tracker.

## 9. Experiment 3: object size

Michotte [1, pp. 82–83] discussed variations in object features and reported that variation in colour, size and shape did not affect the occurrence of the launching effect. In relation to object size he did not number any experiments but reported that ‘various’ experiments were run, using the projection method, in which the objects were circles ranging from 2 to 28 cm in diameter. He commented, ‘In the normal conditions for these experiments – in particular when the point of impact is fixated throughout – the Launching Effect is produced consistently. Sometimes, admittedly, there are differences of degree in this impression, and there are also individual variations between subjects’ (p. 82). But, he concluded, ‘no difference in size, within the limits used... is found to be absolutely incompatible with the Launching Effect’ (p. 82). This rather inexact account leaves open the possibility that the launching effect might vary depending on object size, so Experiment 3 was designed to test this. The reference to a fixation point also suggests that fixation might make a difference to the perceptual impression so the experiment was designed to test that as well.

This experiment is not an exact replication because Michotte did not report sufficient details of stimuli and method to make that possible. To maximize the likelihood of finding an effect if there is one there to be found, a wide range of object sizes was used.

H3. The launching effect will not be affected by manipulations of object size.

### 9.1. Method

Three sizes were used, squares of 2.48, 12.4 and 93 mm, manipulated independently for each object. As in Experiment 2, presence versus absence of a fixation point was manipulated between subjects with 25 participants in each condition.

Instructions to participants in the no-fixation condition were similar to those for Experiment 1 but with two differences. The statement that both rectangles were black was replaced with a statement describing the objects as a black square and a red square and the black and red square terminology was used throughout the instructions. The two statements in Experiment 2, the launching and independent motion statements, were used. Instructions to participants in the fixation condition were similar except that the instructions for fixation from Experiment 2 were added. As in Experiment 2, the experimenter verbally reminded participants of the need to fixate the cross.

### 9.2. Results

Data on the launching measure were analysed with a 2 between (presence versus absence of fixation point) × 3 within (size of black square) × 3 within (size of red square) design. There were no significant results. The output of the analysis is shown in table 8. The range of means was from 7.60 to 9.12, indicating strong launching impressions for all stimuli.

**Table 8.** ANOVA results for Experiment 3, launching measure.

source	SOS	d.f.	MS	*F*	*p*	*η*_p_^2^
fixation (F)	2.57	1	2.57	0.12	0.73	0
error	989.42	48	20.61			
black size (SB)	18.42	2	9.21	2.59	0.08	0.05
F × SB	1.4	2	0.7	0.2	0.2	0
error	341.51	96	3.56			
red size (SR)	18.79	2	9.4	3.76	0.03	0.07
F × SR	4.82	2	2.41	0.96	0.38	0.02
error	239.72	96	2.5			
SB × SR	18.76	4	4.69	1.74	0.14	0.04
F × SB × SR	10.09	4	2.52	0.94	0.44	0.02
error	515.82	192	2.69			

Note: ‘black size’ = size of black square; ‘red size’ = size of red square.

Data on the independent motion measure were analysed with the same design. The output of the analysis is shown in table 9. There was one significant result, the main effect of red size. Post hoc paired comparisons with the Tukey test revealed that the mean for the biggest size was significantly higher than the other two. Means ranged from 1.08 to 2.92, indicating little tendency to see independent motion in any stimulus.

**Table 9.** ANOVA results for Experiment 3, independent motion measure.

source	SOS	d.f.	MS	*F*	*p*	*η*_p_^2^
fixation (F)	3.38	1	3.38	0.14	0.71	0
error	1155.38	48	24.07			
black size (SB)	12.22	2	6.11	1.65	0.2	0.03
F × SB	3.21	2	1.61	0.43	0.65	0.01
error	355.9	96	3.71			
red size (SR)	31.74	2	15.87	5.81	<0.01	0.11
F × SR	8.17	2	4.09	1.5	0.23	0.03
error	262.09	96	2.73			
SB × SR	23.88	4	5.97	1.92	0.11	0.04
F × SB × SR	13.89	4	3.47	1.11	0.35	0.02
error	515.82	192	2.69			

Note: ‘black size’ = size of black square; ‘red size’ = size of red square.

### 9.3. Discussion

There was one significant result, a main effect of size of red object on the independent motion measure: the mean for the biggest object was significantly higher than the means for the other two sizes. Means were all at the low end of the scale, however (less than 2.93). The main effect of red square size on the launching measure was not significant by the criterion chosen here, but *p* < 0.05 so the possibility of an effect of red square size on the launching impression cannot be ruled out. Apart from that, the results were consistent with H3. The results do not, however, establish that object size has no effect on the launching impression, only that any such effect is likely to be weak.

## 10. Experiment 4: delay

Experiment 4 is a replication of experiment 29, in which delay was introduced between the black square contacting the red square and the red square starting to move. Michotte used 13 delays in increments of 14 ms from 14 to 182 ms. This cannot be exactly replicated with the present technology because the time span of a single frame is 16.7 ms, so 13 delays in increments of 16.7 ms were used, from 0 to 200.0 ms.

Michotte [1] reported that, even with a delay of 70 ms, reporting of the launching effect was reduced, and, with a delay of 154 ms, it did not occur. He reported that, at intermediate delays, the launching effect occurred but with some time lag: ‘Object B [the red square] “sticks” to object A [the black square]; its departure takes place only after some delay’ (p. 92). This ‘delayed launching’ impression was the predominant response with delays around 98 ms. After that it declined and perception of independent motion increased. Replication therefore requires inclusion of a statement based on Michotte’s description of this delayed launching impression.

Several subsequent studies have manipulated delay. Three studies presenting incremental delays similar to those used by Michotte [1] found similar rapid declines in reported perceptual causality as delay increased beyond 50 ms to about 200 ms [36,53,68]. Results of other studies suggest that sensitivity to delay might not be as acute as Michotte [1] reported. Meding *et al*. [69] had a delay manipulation with several delays from 0 to 400 ms and found a decline in ratings as delay increased, but even with zero delay the mean rating was a little above the mid-point of their scale. Guski & Troje [27] found a steeper decline from a higher mean at zero delay. Schlottmann *et al*. [8] presented a launching stimulus with a delay of 1250 ms and found that 8% of 72 participants gave spontaneous descriptions suggestive of physical causality. Considering only those who saw the delay stimulus before any of the others, 50% (6/12) gave physical causality responses. Bechlivanidis *et al*. [38] used a stimulus with 250 ms delay. If the delay stimulus was the first one presented, mean ratings were above 60 on a 101-point scale. If the delay stimulus was then presented again after a typical launching stimulus with zero delay, mean ratings were significantly lower, and below the scale mid-point. This change in ratings suggests that at least some participants, were, initially, reporting a post-perceptual judgement rather than a perceptual impression: a perceptual impression would not change significantly after only three stimulus presentations. The likelihood of post-perceptual judgement being involved was increased by the wording of the question for the rating task, which was that used by Schlottmann *et al*. [8], except for a change in the colour of the second object: ‘Do you have the impression that red somehow made blue move?’ [38, p. 789]. The word ‘somehow’ invites speculation which is perhaps undesirable in a study of perception and ‘having an impression’ can refer to non-perceptual cognitive processes in common parlance—e.g. ‘I had the impression that he didn't like me’. So it is not certain that participants were reporting visual impressions of causality.

Overall, therefore, results for delay manipulations have been variable. It seems likely that wording of the statement or question to be rated is of some importance and merits further investigation. As a first step forward, this study was designed to replicate as closely as possible the stimuli that Michotte used, and with a form of wording in the instructions that emphasized the need to report a visual impression. Comparison of such a form of words with those used in the other studies cited here should be a priority for future research.

H4. The launching effect will weaken as delay increases. At intermediate delays the delayed launching impression will dominate and at longer delays independent motion will be perceived.

### 10.1. Method

There was a single variable, delay at contact, with 13 delays ranging from 0 to 200.0 ms in increments of 16.7 ms. Instructions to participants were as in Experiment 3 (no-fixation condition) except that three statements were presented for rating, as follows:

The black square made the red square move by bumping into it.

The black square made the red square move by bumping into it, but the red square seemed to ‘stick’ to the black square briefly before moving off.

The red square moved independently and its motion was not caused by the black square.

The second of these was designed to capture Michotte’s description of the delayed launching impression.

### 10.2. Results

**Figure 5.**
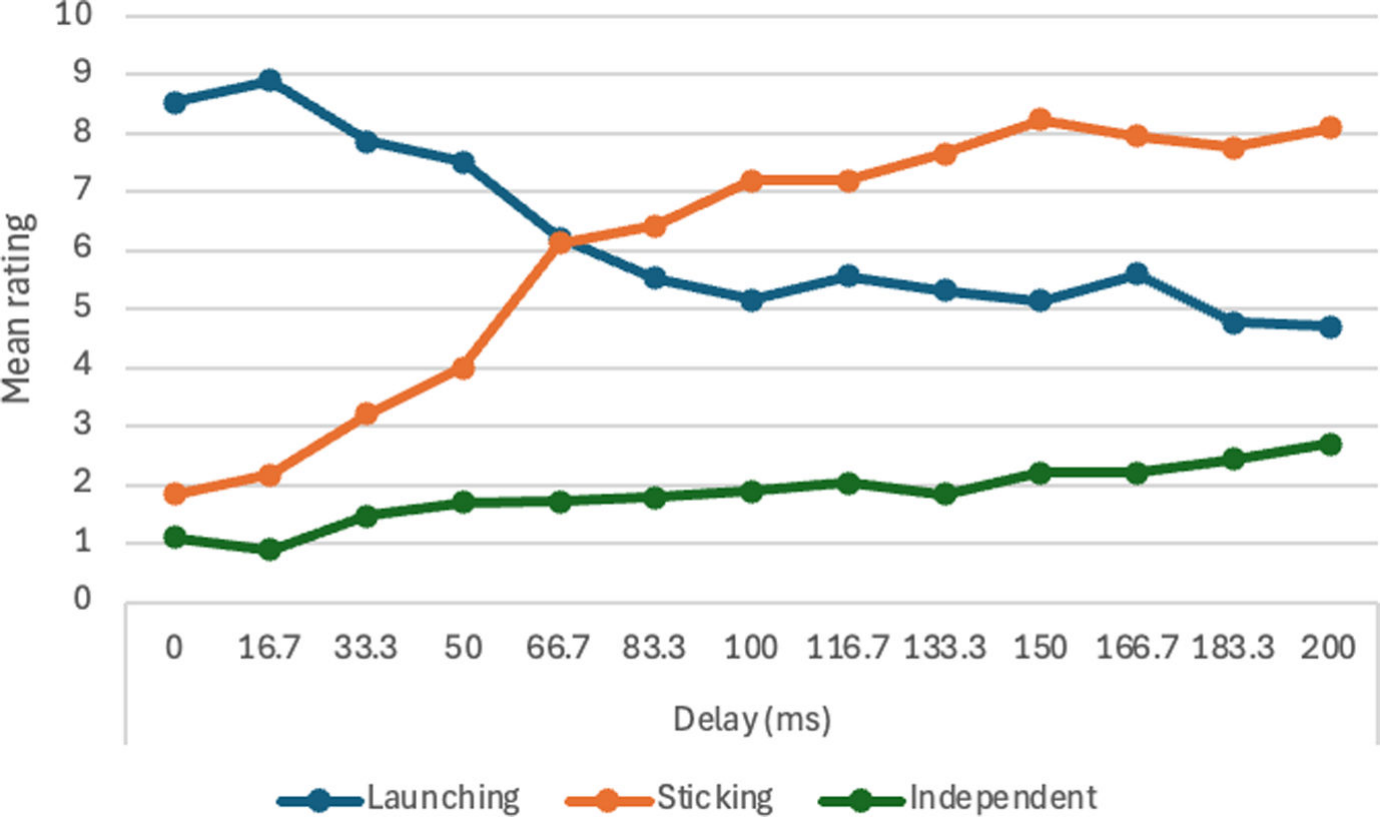
Mean ratings on launching, sticking and independent measures with increasing delay, Experiment 4.

**Figure 6.**
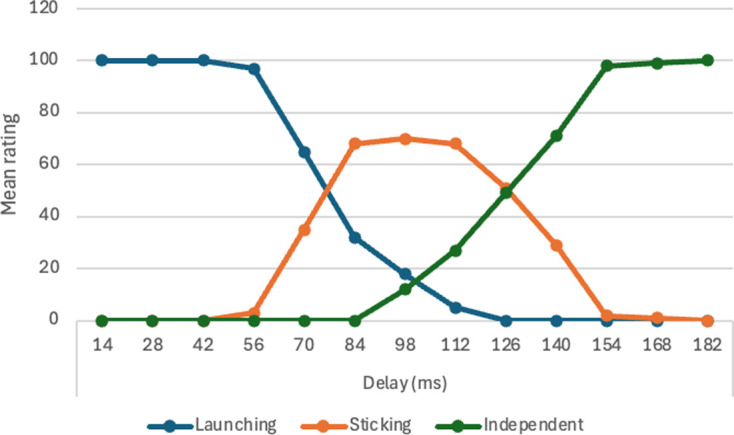
Results reported by Michotte [1] for the delay manipulation.

Each measure was analysed separately with one-way ANOVA. For the launching measure, there was a significant effect, *F* (12, 588) = 19.22, m.s.e. = 5.57, *p* < 0.001, η_p_^2^ = 0.28. For the sticking measure there was a significant effect, *F* (12, 588) = 41.60, m.s.e. = 6.59, *p* < 0.001, η_p_^2^ = 0.46. For the independent motion measure there was a significant effect, *F* (12, 588) = 4.17, m.s.e. = 3.02, *p* < 0.001, η_p_^2^ = 0.08. Means and results of post hoc paired comparisons with the Tukey test are reported in table 10. Means are depicted in figure 5. Table 11 reports results of one-way ANOVAs on individual stimuli. Figure 6 depicts the results reported by Michotte [1].

**Table 10.** Means on all measures, Experiment 4.

delay (ms)	launching	sticking	independent
0	8.54^a^	1.84^a^	1.10^ab^
16.7	8.90^a^	2.18^a^	0.90^a^
33.3	7.86^b^	3.22^b^	1.48^abc^
50	7.52^b^	4.00^b^	1.70^abcd^
66.7	6.22^c^	6.12^c^	1.72^abcd^
83.3	5.54^c^	6.42^cd^	1.80^abcd^
100	5.16^c^	7.20^cde^	1.90^abcd^
116.7	5.56^c^	7.20^cde^	2.04^abcd^
133.3	5.32^c^	7.66^cde^	1.84^abcd^
150	5.14^c^	8.24^e^	2.22^bcd^
166.7	5.60^c^	7.96^de^	2.22^bcd^
183.3	4.78^c^	7.76^cde^	2.44^cd^
200	4.70^c^	8.10^de^	2.70^d^

Means within columns not sharing the same superscript differ by *p* < 0.05 (Tukey).

**Table 11.** Comparisons between measures, Experiment 4.

delay (ms)	*F*	m.s.e.	*p*	*η*_p_^2^	differences
0	144.32	5.9	<0.001	0.75	L > S & I
16.7	177.19	5.15	<0.001	0.78	L < S > I
33.3	54.98	10.01	<0.001	0.53	L > S & I
50	40.92	10.41	<0.001	0.46	L > S > I
66.7	21.76	13.7	<0.001	0.31	L & S > I
83.3	20.55	13.08	<0.001	0.3	L & S > I
100	21.23	15.04	<0.001	0.3	S > L > I
116.7	18.17	14.39	<0.001	0.27	S & L > I
133.3	29.25	12.66	<0.001	0.3	S > L > I
150	34.39	12.14	<0.001	0.41	S > L > I
166.7	20.53	15.07	<0.001	0.3	S & L > I
183.3	20.91	13.92	<0.001	0.3	S>L > I
200	29.15	13.04	<0.001	0.37	S > L > I

Note: L = Launching measure; S = Sticking measure; I = Independent motion measure. d.f. = 2, 98.

### 10.3. Discussion

According to Michotte [1], with a delay of 70 ms, reporting of the launching effect was reduced compared with no delay. Here there was even finer sensitivity, with a delay of 33.3 ms being rated significantly lower on the launching measure, and significantly higher on the sticking measure, than 0 and 16.7 ms delay. This might just reflect greater sensitivity of rating scale measures over the all-or-nothing reports in Michotte’s research, but the fact remains that the launching effect is acutely sensitive to delay at contact. In Michotte’s study, ratings of delayed launching peaked at 98 ms delay. The present results closely resembled that ratings on the sticking measure rose steadily up to about 100.0 ms.

At delays beyond 83.3 ms, however, the present results diverged from those reported by Michotte [1], as visual comparison between figures 5 and 6 shows. Ratings of launching declined as far as a delay of 66.7 ms but then dropped no further and remained around the middle of the scale even at the longest delay used here, 200.0 ms. This contrasts with Michotte’s report that reports of launching continued to decline and reached zero at and beyond 154 ms. With delays longer than 100 ms, delayed launching reports declined in Michotte’s study whereas they remained high in the present study through to 200.0 ms. In Michotte’s study, reports of independent motion increased after 98 ms until they constituted 100% of responses. In the present study independent motion was rated lower than both launching and sticking at all delays and indeed the highest mean rating of independent motion was only 2.70, for 200.0 ms delay.

The lack of further decline in ratings of launching at longer delays is consistent with results reported by Meding *et al*. [69] and Bechlivanidis *et al*. [38]. There is some evidence suggesting that ratings in those studies might have reflected post-perceptual judgements, as if the launching effect did not occur but observers still thought that the first object must have made the second one move. That possibility could apply here too. Participants were instructed to base their ratings on their visual experience, but it is impossible to know whether all of them actually did so. There is still uncertainty, therefore, over what is perceived at delays longer than 100 ms.

In summary, there is support for the first two components of H4 but not for the third component, because the evidence is consistent with the possibility that independent motion was not perceived at any delay.

## 11. Experiment 5: pausing of a single object in motion

This was a replication of experiment 30. In that experiment, there was just a single object that moved for a distance equal to that of the combined motions of the black and red squares in experiment 29. A pause in the movement occurred halfway through. Pause durations were manipulated in the same way as delay durations in experiment 29. Michotte [1] reported that short pauses were not perceived; that is, motion was perceived as continuous. At pauses of moderate duration, a percept of discontinuity was reported ‘which is still compatible with the unity of the whole, i.e. the “movement in two stages”’ (p. 96). That impression peaked with a pause duration of 70–87 ms. With longer pause durations there was an impression ‘of a halt, or definite pause, and together with this the impression of two separate movements’ (p. 96).

The importance of experiment 30 is that the effect of the pause was closely correlated with the effect of delay in experiment 29. The launching effect was reported for delay durations that matched pause durations where motion was reported as continuous. At pause durations where motion was perceived as discontinuous (in experiment 30), the percept of delayed launching tended to occur (in experiment 29); and, at durations where motion was perceived as having two components with a halt between them (in experiment 30), the percept of independent motion tended to dominate (in experiment 29). This suggests that the perceptual impression of causality might depend critically on perception of continuity of motion across the two objects, which could have significant theoretical implications. Experiment 5 was therefore designed with a single object in motion and with incremental pause durations matching those used in Experiment 4. It was also planned to calculate correlations on data from the two experiments.

H5. The impression of continuous motion will decline as pause duration increases. At intermediate pause durations the percept of discontinuous motion will dominate and at longer delays two motions with a halt between them will be perceived.

H6. There will be high positive correlations between launching ratings (Experiment 4) and continuous motion ratings, between delayed launching ratings (Experiment 4) and discontinuous motion ratings, and between independent motion ratings (Experiment 4) and ratings of two motions with a halt between them.

None of the participants in this experiment were participants in Experiment 4.

### 11.1. Method

The experiment involved stimuli in which a black square moved across the screen on the same motion path as the combined motions of the black and red squares in the corresponding animations in Experiment 4. Halfway through this motion (equivalent to the point of contact between the objects in the Experiment 4 stimuli) a pause was introduced with 13 durations increasing in increments of 16.7 ms from 0 to 200.0 ms. Thus, the pause durations in this experiment matched the delay durations in Experiment 4. Three statements were created for the rating task designed to reflect Michotte’s descriptions of the impressions that occurred, as follows:

The motion of the black square seems continuous without any break or pause.

The motion of the black square seems like a single movement but in two stages with a brief discontinuity or pause in the middle.

There is an impression of two separate movements with a halt or definite pause in the middle.

### 11.2. Results

**Figure 7.**
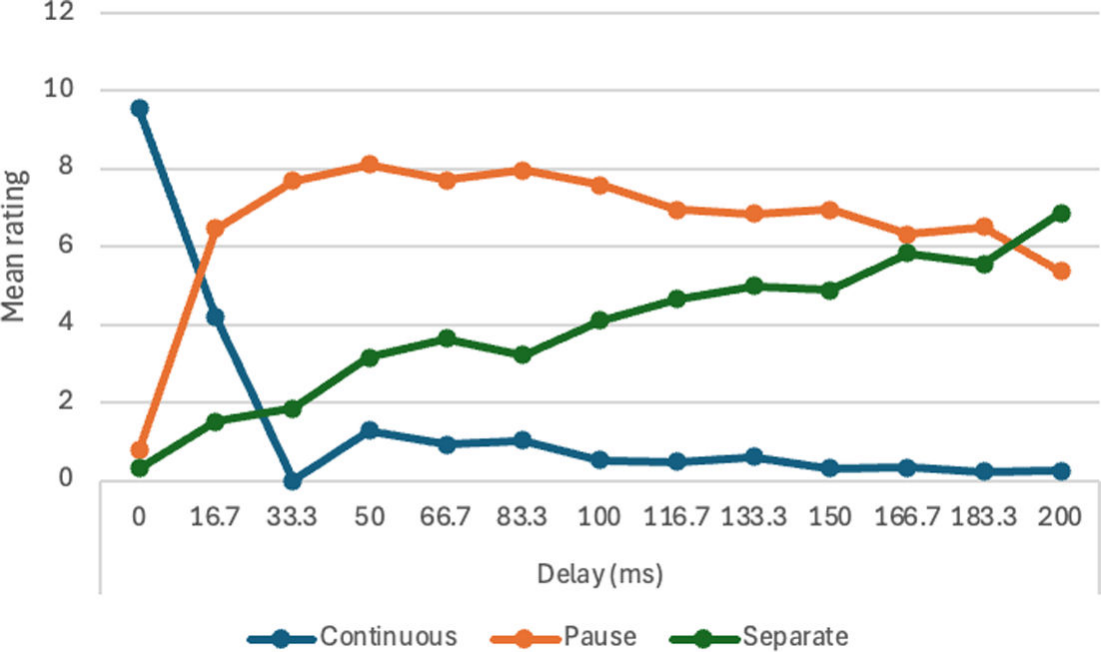
Mean ratings on continuous, pause and separate measures with increasing delay, Experiment 5.

**Figure 8.**
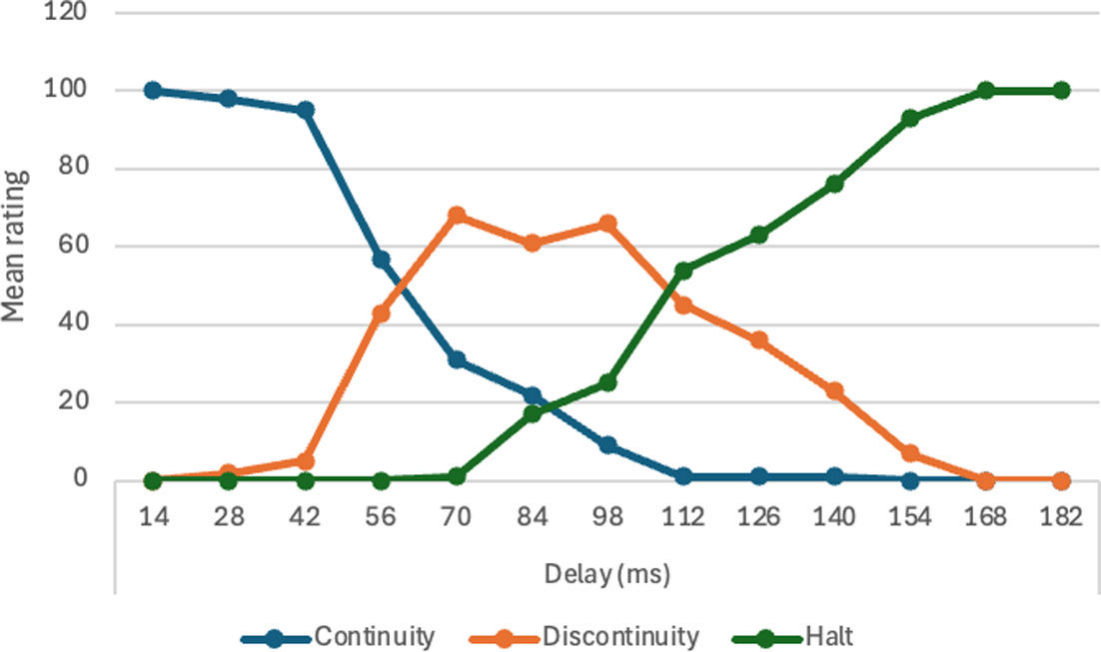
Results reported by Michotte for the pause manipulation.

Each measure was analysed separately with one-way ANOVA. For the continuous measure there was a significant effect, *F* (12, 588) = 96.45, m.s.e. = 3.55, *p* < 0.001, η_p_^2^ = 0.66. For the brief pause measure there was a significant effect, *F* (12, 588) = 24.86, m.s.e. = 7.26, *p* < 0.001, η_p_^2^ = 0.34. For the separate motions measure, there was a significant effect, *F* (12, 588) = 25.31, m.s.e. = 6.88, *p* < 0.001, η_p_^2^ = 0.34. Means and results of post hoc paired comparisons with the Tukey test are reported in table 12. Means are depicted in figure 7. Table 13 reports results of one-way ANOVAs on individual stimuli. Figure 8 depicts the results reported by Michotte [1].

**Table 12.** Means on all measures, Experiment 5.

delay (ms)	continuous	pause	separate
0	9.54^a^	0.78^a^	0.32
16.7	4.20^b^	6.46^bc^	1.52
33.3	2.80^c^	7.70^c^	1.86
50	1.28^d^	8.12^c^	3.16
66.7	0.92^d^	7.72^c^	3.64
83.3	1.04^d^	7.96^c^	3.22
100	0.52^d^	7.58^c^	4.1
116.7	0.48^d^	6.96^bc^	4.65
133.3	0.62^d^	6.84^bc^	5
150	0.32^d^	6.96^bc^	4.88
166.7	0.34^d^	6.32^bc^	5.84
183.3	0.24^d^	6.52^bc^	5.56
200	0.26^d^	5.38^b^	6.86

Note. Means within columns not sharing the same superscript differ by *p* < 0.05 (Tukey).

**Table 13.** Paired comparisons between measures, Experiment 5.

delay (ms)	*F*	m.s.e.	*p*	*η*_p_^2^	differences
0	468.96	2.88	<0.001	0.91	C > P & S
16.7	17.75	17.22	<0.001	0.27	P > C > S
33.3	35.22	13.06	<0.001	0.42	P > C & S
50	58.98	10.58	<0.001	0.55	P > S > C
66.7	73.66	7.95	<0.001	0.6	P > S > C
83.3	79.09	7.91	<0.001	0.62	P > S > C
100	73.76	8.45	<0.001	0.6	P > S > C
116.7	56.77	9.5	<0.001	0.54	P > S > C
133.3	42.16	12.09	<0.001	0.46	P > S > C
150	53.6	10.76	<0.001	0.52	P > S > C
166.7	47.59	11.56	<0.001	0.49	P & S > C
183.3	52.99	10.8	<0.001	0.52	P & S >C
200	57.1	10.5	<0.001	0.54	P & S > C

C = Continuous measure; *p* = Pause measure; S = Separate movements measure. d.f. = 2, 98.

### 11.3. Discussion

The main feature of the results was a very rapid decline in ratings on the continuous measure with increasing pause duration, from a mean of 9.54 at zero pause to 4.20 at 16.7 ms pause, further declining to 1.28 at 50.0 ms pause. Even though motion is not truly continuous on the screen, but comprises a series of jumps in object position, the results show that a temporal discontinuity in that sequence of events of only 16.7 ms could be detected. Ratings on the pause measure showed a correspondingly rapid increase from a mean of less than 1 at 0 ms pause to 6.46 at 16.7 ms pause. Ratings peaked at 50.0 ms but only showed statistically significant decline at the longest pause of 200.0 ms. Ratings of separate motion rose steadily with increasing pause duration but at no pause duration was separate motion rated significantly higher than both of the other ratings.

Comparison between figures 7 and 8 illustrates how the present results differ from those reported by Michotte [1]. He found no appreciable decline in reports of continuous motion at delays shorter than 56 ms. Reports of pause or discontinuity peaked with a delay of 70 ms, close to what was found here, but then declined rapidly and reached zero by 168 ms pause, which was not found here. Reports of a halt dominated from a delay of 126 ms on; that was not found here.

It is not clear what would account for these differences. They could be due to differences in the technology. However it must again be pointed out that the stimuli presented by Michotte were genuinely continuous and it seems likely that that would make it easier to detect brief discontinuities in motion than it was with the objectively discontinuous stimuli in the present experiment, not harder. Differences in word meaning or interpretation of the instructions could be a factor, but the wording here was deliberately based on that used by Michotte, so it seems unlikely that any minor differences in wording would have such a large effect on the results. The participants in Michotte’s study, both the delay manipulation in experiment 29 and the pause manipulation in experiment 30, were three experienced observers, including Michotte himself, whereas those in Experiments 4 and 5 here were two different samples each of 50 naive participants. Whether this might account for the difference in results is not clear, mainly because it is not clear how the experience and attitudes of the observers in Michotte’s study, as well as the interactions between them, might affect their reports. The present experiment merely scratches the surface: perception of motion discontinuity could be affected by many factors, so further investigation could be illuminating.

In summary, H5 is partly supported in that the impression of continuous motion did decline as pause duration increased. In other respects, however, the results differed from those reported by Michotte and do not fit well with H5.

## 12. Comparisons between Experiment 4 and Experiment 5

Comparisons between data from Experiments 4 and 5 were analysed to test whether the similarities found by Michotte and described above would hold here. H6 was expressed in correlational terms, but it is better tested by *t*-test or one-way ANOVA, to clarify the differences found. Thus, at each value of delay, launching ratings (Experiment 4) were compared with continuous ratings (Experiment 5), sticking ratings (Experiment 4) with pause ratings (Experiment 5), and independent motion ratings (Experiment 4) with separate motion ratings (Experiment 5).

### 12.1. Results

Results of analyses are reported in tables 14 (launching versus continuous), 15 (sticking versus pause) and 16 (independent versus separate).

**Table 14.** Comparisons between launching ratings (Experiment 4) and continuous ratings (Experiment 5).

delay (ms)	*F*	m.s.e.	*p*	*η*_p_^2^	differences
0	8.7	3.39	<0.001	0.08	C > L
16.7	53.3	9.67	<0.001	0.35	L > C
33.3	61.21	10.37	<0.001	0.38	L > C
50	139.45	7.03	<0.001	0.59	L > C
66.7	44.81	7.43	<0.001	0.31	L > C
83.3	57.25	9.16	<0.001	0.22	L > C
100	63.5	8.55	<0.001	0.39	L > C
116.7	77.55	8.25	<0.001	0.44	L > C
133.3	62.24	8.95	<0.001	0.32	L > C
150	74.8	7.76	<0.001	0.43	L > C
166.7	94.65	7.53	<0.001	0.49	L > C
183.3	71.53	7.46	<0.001	0.42	L > C
200	68.28	7.55	<0.001	0.41	L > C

Note. L = launching; C = continuous.

**Table 15.** Comparisons between sticking ratings (Experiment 4) and pause ratings (Experiment 5).

delay (ms)	*F*	m.s.e.	*p*	*η*_p_^2^	differences
0	5.51	5.9	<0.001	0.05	S > P
16.7	37.34	12.26	<0.001	0.28	P > S
33.3	36.61	11.03	<0.001	0.28	P > S
50	44.95	9.35	<0.001	0.31	P > S
66.7	6.44	9.93	<0.05	0.06	P > S
83.3	8.65	7.96	<0.01	0.08	P > S
100	0.51	8.64	0.38	0	
116.7	0.09	8.96	0.13	0	
133.3	1.27	9.07	0.26	0.02	
150	4.13	8.13	<0.05	0.04	S > P
166.7	6.29	10.17	<0.05	0.06	S > P
183.3	4.52	8.77	0.04	0.04	
200	15.4	10.64	<0.001	0.14	S > P

S = sticking; P = pause.

**Table 16.** Comparisons between independent motion ratings (Experiment 4) and separate motion ratings (Experiment 5).

delay (ms)	*F*	m.s.e.	*p*	*η*_p_^2^	differences
0	5.83	2.49	<0.05	0.06	I > S
16.7	2.78	4.25	0.1	0.02	
33.3	0.71	5.09	0.33	0.01	
50	6.33	8.42	<0.05	0.06	S > I
66.7	11.86	7.77	<0.001	0.11	S > I
83.3	6.51	7.74	<0.05	0.06	S > I
100	13.04	9.28	<0.001	0.12	S > I
116.7	19.85	8.64	<0.001	0.17	S > I
133.3	25.87	9.53	<0.001	0.21	S > I
150	16.77	10.55	<0.001	0.15	S > I
166.7	25.76	10.83	<0.001	0.21	S > I
183.3	21.13	11.52	<0.001	0.18	S > I
200	45.55	10.43	<0.001	0.32	S > I

Note. I = independent motion; S = separate motion.

### 12.2. Discussion

On comparisons between launching (Experiment 4) and continuous (Experiment 5) ratings, at zero delay there was a significantly higher mean on the continuous measure than on the launching measure. On all other stimuli launching ratings were significantly higher than continuous ratings. On comparisons between sticking (Experiment 4) and pause (Experiment 5) ratings, at zero delay there was a significantly higher rating on the sticking measure than on the pause measure. This is a little odd, since there was no discontinuity in motion with the zero delay stimulus, but both means were close to zero. At delays from 16.7 to 83.3 ms there were significantly higher ratings on the pause measure than on the sticking measure. At delays of 150.0, 166.7 and 200.0 ms, the opposite was the case. No significant difference was found on the remaining four delays. On comparisons between independent (Experiment 4) and separate (Experiment 5) motion, at zero delay there was a significantly higher mean on the independent motion measure than on the separate motion measure. On all delays from 50.0 to 200.0 ms, there were significantly higher means on the separate motion measure than on the independent motion measure. At 16.7 and 33.3 ms there was no significant difference.

The results show that there is no parallel to be drawn between launching and continuous motion percepts, between sticking (with launching stimuli) and pausing (with single object stimuli), or between independent motion (with launching stimuli) and separate motion (with single object stimuli). They are just different phenomena. Whatever determines the transition from launching to sticking or delayed launching, and from sticking to independent motion, it is not the mere perception of motion discontinuity. The results do not resemble what Michotte reported. H6 can be rejected.

## 13. Experiment 6: gap

This experiment was based on experiment 31 in which the projection method was used and the stimuli were projected discs of light. The first moving object (a disc of light 35 mm in diameter) stopped before reaching the initially stationary object (a similar disc of light). Michotte reported that the launching effect could occur despite the presence of a gap between them. The reporting of results is anecdotal but it is clear that speed was a critical factor, and that the launching effect could occur despite the presence of a substantial gap if the speed was sufficiently great: Michotte reported that even a gap of 500 mm ‘did not necessarily make the causal impression disappear’ [1, p. 100]. Yela [70] ran a study with 250 naive participants and found that the numbers reporting the launching effect fell from 100% with zero gap to 28% with a 90 mm gap. A further study Yela [70] included a delay manipulation and found that the effect of delay on the launching effect was similar for all gap sizes, up to a maximum of 50 mm. Some studies since then have reported very low causal ratings with even quite small gaps [8,12,53,71]. Perhaps the most extreme result was that reported by Sanborn *et al*. [53]: with speeds ranging from 60 to 150 mm s^-1^, ratings in their causal judgement task were low with gaps as small as 2 mm. There is a striking contrast between these recent results and those reported by Michotte [1] and Yela [70].

This brief review indicates that there is some uncertainty about the effect of gaps on the causal impression, and particularly about the role of object speed. Some studies have used gap stimuli as non-causal controls for launching effect stimuli [12,57,72–74]; the results reported by Michotte [1] and Yela [70] suggest that this might be inadvisable unless the gap is large.

Exact replication of experiment 31 is not possible, partly because of technological differences and partly because of the inexactness in the reporting of manipulations and results [1]. Also, the largest gaps used by Michotte [1] are greater than the size of the screen to be used for the present experiment. It was decided to sample a range of gaps up to the maximum used by Yela [70], 90 mm. Given the likely importance of object speed, as reported by Michotte [1], speed (of both objects) was also manipulated.

H7. The launching effect will decline as gap size increases.

H8. For all gap sizes, the launching effect will increase as object speed increases.

### 13.1. Method

There were two independent variables. Gap size was manipulated with seven values, 3.1, 6.2, 12.4, 24.8, 46.5, 68.2 and 89.9 mm. Three speeds were used, 74.3, 124.0 and 186.0 mm s^-1^, with both objects having the same speed in any given stimulus. This makes a 7 within (gap size) × 3 within (speed) ANOVA design.

The instructions needed to be modified to take account of the fact that the black square does not come into contact with the red square. The first paragraph of the instructions therefore read as follows: ‘In this experiment you will see a series of short movies, about one or two seconds in duration, each involving two objects, a black square and a red square. Each movie will begin with the black square moving towards the red square. We are interested in what you see when the black square stops moving and the red square starts moving, the visual impression you have of the movies, not any thoughts you might have about what you are seeing. It may still be possible to have a visual impression that the black square made the red square move, even when they do not come into contact. For each movie you will be asked to rate the extent to which you agree or disagree with each of two statements as descriptions of your visual impression of what happened. You should rate your agreement or disagreement with each of the statements based just on your visual impression, not on what you think is possible.’ The two statements were as follows:

‘The black square made the red square move.

The red square moved independently and its motion was not caused by the black square.’

### 13.2. Results

#### 13.2.1. Launching measure

There was a significant effect of speed, *F* (2, 98) = 9.87, m.s.e. = 3.12, *p* < 0.001, η_p_^2^ = 0.17. Post hoc paired comparisons with the Tukey test revealed a significantly higher mean at 186.0 mm s^-1^ than at the other two speeds, which did not differ significantly. There was a significant effect of gap size, *F* (6, 294) = 44.86, m.s.e. = 6.28, *p* < 0.001, η_p_^2^ = 0.48. Significant differences revealed by post hoc paired comparisons are shown in table 17. The interaction was not significant, *F* (12, 588) = 1.30, m.s.e. = 2.89, *p* = 0.21, η_p_^2^ = 0.03. Means are shown in table 17.

**Table 17.** Mean ratings, launching measure, Experiment 6.

	speed (mm s^-1^)			
gap size (mm)	74.3	124	186	All
3.1	6.04	6.08	6.84	6.32^a^
6.2	4.8	5.34	5.74	5.29^b^
12.4	3.54	3.96	4.6	4.03^c^
24.8	3.84	3.64	4.1	3.86^cd^
46.5	3.14	3.22	3	3.12^de^
68.2	2.18	2.74	3.2	2.71^ef^
89.9	2.5	2.84	2.7	2.68^ef^
All	3.72^a^	3.97^a^	4.31^b^	

Note. Means not sharing the same superscript differ by *p* < 0.05 (Tukey).

#### 13.2.2. Independent motion measure

There was a significant effect of speed, *F* (2, 98) = 7.52, m.s.e. = 2.69, *p* < 0.001, η_p_^2^ = 0.13. Post hoc paired comparisons with the Tukey test revealed a significantly higher mean at 74.3 mm s^-1^ than at 186.0 mm s^-1^, with the mean at 124.0 mm s^-1^ not differing significantly from either of those. There was a significant effect of gap size, *F* (6, 294) = 44.80, m.s.e. = 5.30, *p* < 0.001, η_p_^2^ = 0.48. Significant differences revealed by post hoc paired comparisons are shown in table 18. The interaction was not significant, *F* (12, 588) = 1.38, m.s.e. = 3.07, *p* = 0.17, η_p_^2^ = 0.03. Means are shown in table 18.

**Table 18.** Mean ratings, independent motion measure, Experiment 6.

	Speed (mm s^-1^)			
gap size (mm)	74.3	124	186	All
3.1	4.02	4.24	3.38	3.88^a^
4.2	5.46	4.96	4.76	5.06^b^
12.4	6.7	6.26	5.56	6.17^c^
24.8	6.42	6.28	5.98	6.23^c^
46.5	6.74	6.74	7.1	6.86^d^
68.2	7.8	7.2	7.04	7.35^d^
89.9	7.28	7.26	7.24	7.26^d^
All	6.35^a^	6.13^ab^	5.87^b^	

Note. Means not sharing the same superscript differ by *p* < 0.05 (Tukey).

#### 13.2.3. Analyses of individual stimuli

Ratings of each stimulus were analysed with one-way repeated measures ANOVA and results are shown in table 19.

**Table 19.** Analyses of individual stimuli, Experiment 6.

speed	gap size	*F*	m.s.e.	*p*	*η*_p_^2^	differences
74.3	3.1	5.06	20.55	<0.05	0.09	L > I
	6.2	0.69	19.79	0.46	0.01	
	12.4	12.79	19.77	<0.001	0.21	I > L
	24.8	5.91	22.78	<0.05	0.11	I > L
	46.5	15.1	19.37	<0.001	0.24	I > L
	68.2	64.11	11.88	<0.001	0.57	I > L
	89.9	34.82	15.32	<0.001	0.42	I > L
124	3.1	4.3	19.68	0.04	0.07	
	6.2	0.13	23.01	0.7	0	
	12.4	7.48	21.55	<0.01	0.13	I > L
	24.8	9.17	19.88	<0.01	0.16	I > L
	46.5	15	20.15	<0.001	0.23	I > L
	68.2	24.42	19.46	<0.001	0.33	I > L
	89.9	24.33	17.95	<0.001	0.33	I > L
186	3.1	9.33	24.12	<.001	0.16	L > I
	6.2	0.95	25.3	0.33	0.01	
	12.4	0.9	24.52	0.35	0.01	
	24.8	4.71	23.39	<0.05	0.09	I > L
	46.5	20.11	20.09	<0.001	0.29	I > L
	68.2	19.75	18.66	<0.001	0.29	I > L
	89.9	34.23	15.59	<0.001	0.41	I > L

Note. L = Launching; I = Independent motion. d.f. = 1, 49.

### 13.3. Discussion

The results showed significant tendencies for launching ratings to decline as gap size increased, and to rise as object speed increased, supporting H7 and H8. In this experiment, the presence of a gap had a detrimental effect on the launching effect even at its smallest value. For purposes of comparison, the range of means on the launching effect found in Experiment 3, which presented nine standard launching stimuli manipulating only object size, was from 7.60 to 9.12. The highest mean launching rating found in the present experiment was 6.84, for the highest speed and smallest gap, smaller than any found in Experiment 3. Furthermore, there were only two stimuli for which the mean launching rating was significantly higher than the mean independent rating; those were two of the three stimuli with the smallest gap size (see table 19).

It is not possible to say that an impression of launching did not occur at all at the largest gap size. The lowest launching mean found was 2.18 (in fact, for the second largest gap size). This is well below the lowest launching mean found in Experiment 4, which was 4.70 (for 200.0 ms delay), but also well above the lowest mean found on the continuous measure in Experiment 5, which was 0.24 (for 183.3 ms delay). Yela [70] found that 28% of participants reported the launching effect with a gap of 90 mm. In that experiment, the causal object moved at 300 mm s^-1^, compared with a top speed of 186 mm s^-1^ used here, and the effect object moved at 45 mm s^-1^. Given that the effect on launching ratings of tripling the speed, although statistically significant, was quite small in the present experiment, the present results do not appear inconsistent with those reported by Yela [70]. Perhaps some people perceive launching with large gaps and others do not; perhaps most people have a weak launching impression and use different criteria for deciding whether it is really there or not. It is worth pointing out, though, that using a gap stimulus as a non-causal control stimulus, as has been done in several published experiments, is not justified, given the evidence that the launching impression can occur, if weakly and not in all observers, even with substantial gaps. It would be better to use a stimulus as similar as possible to a launching stimulus but for which no causal impression occurs.

The smallest gap size used here was 3.1 mm, greater than the gap size of 2 mm used by Sanborn *et al*. [53]. The present results, showing fairly high launching ratings with 3.1 mm gap, are therefore not consistent with the low ratings reported by Sanborn *et al*. [53] for the 2 mm gap. This is probably attributable to the instructions. In Sanborn *et al*. [53], participants were told to decide whether the movie ‘came from a real collision of the blocks or a random combination of the variables. A real collision looks like the blocks actually collide’ (p. 421). It is likely, therefore, that participants just judged whether the blocks came into contact or not and judged that a real collision did not occur if they did not perceive contact. It was probably not a study of the launching effect at all.

Schlottmann & Anderson [71] presented stimuli with gaps of 0, 0.7, 1.4 and 2.1 mm, all smaller than the smallest gap used here, 3.1 mm. At the minimum delay of 17 ms (there was no zero delay condition), ratings dropped rapidly as gap size increased, to about the scale mid-point with a gap of 2.1 mm. That is not consistent with the present results. The question asked of participants was, ‘Did it look like *B* moved because *A* hit it? Was *B*’s movement produced by *A*? – Or did *B* take off on its own?’ (p. 788). The word ‘hit’ implies contact, so it is likely that the ratings fell rapidly with increasing gap size because participants did not perceive contact between the objects. This underlines the importance of wording of measures in rating scale studies. The wording used here was ‘The black square made the red square move’, with instructions emphasizing the importance of reporting the visual impression. This form of the words does not imply contact between the objects, and that might account for the difference in results between the present study and that by Schlottmann & Anderson [71].

In summary, much depends on wording of instructions. Even with appropriate wording, launching ratings decline rapidly as gap size increases, but do not fall to zero even with very large gaps.

## 14. Experiment 7: chasing

This is based on experiment 17. In that experiment, the two objects started moving at the same time and in the same direction. The black square moved faster than the red square and caught up with it. When the black square contacted the red square the former stopped and the latter continued to move. The stimulus resembles the typical stimulus for launching except for the motion of the red square prior to contact. Michotte [1] reported that the launching effect occurred with those stimuli but not so much if the black square’s speed was only a little faster than that of the red square. Michotte also claimed that the launching effect occurred if the speed of the red square did not change after contact, and even if the red square slowed down after contact. Speeds and distances moved cannot be exactly the same as those used by Michotte [1], but a range of speed ratios was devised that overlaps with the range used by Michotte. To achieve this, the speed of the red square before contact was held constant at the 37.2 mm s^-1^ and the speed of the black square was manipulated.

Michotte’s [1] experiment 49 was an entraining version of experiment 17. He reported that the entraining effect occurred if the black square was fixated but not if the red square was fixated. Experiment 9 below is based on experiment 49 and manipulates fixation. To make Experiment 7 and Experiment 9 as similar as possible, therefore, fixation was also manipulated in this experiment, and it is predicted that the effect of fixation reported by Michotte will be found in this experiment as well.

H9. Ratings of launching will be above the scale mid-point for all stimuli. This is based on the impressions reported by Michotte and described above.

H10. There will be a main effect of fixation with higher means when the black square is fixated than when the red square is fixated.

### 14.1. Method

In this experiment, the red square moved before contact at 37.2 mm s^-1^ and the speeds of the black square were set to bring about speed ratios of 2:1, 3:1, 4:1 and 6:1. After contact the red square moved at either 74.4 mm^-1^ s, 37.2 mm^-1^ s (the same as the speed before contact), or 18.6 mm s^-1^. In addition, a fixation manipulation was included as a between-subjects variable with 25 participants in each of two conditions. Participants were instructed to fixate the black square in one condition and the red square in the other. This resulted in a 2 between (fixation, black square versus red square) × 4 within (speed ratio, 2:1 versus 3:1 versus 4:1 versus 6:1) × 3 within (red square post-contact speed, 74.4 versus 37.3 versus 18.6 mm s^-1^) ANOVA design.

Speeds were at the slow end of the range used by Michotte but the limited size of the computer screen imposes certain constraints on speed: if both objects are in motion at speeds that are not very different, for one to catch up with the other requires a lot of space, especially if the speeds are fast.

Wording of statements for the rating task is problematic in this experiment. It would not be right to have a statement saying that the black square made the red square move because participants might disagree with this on the grounds that the red square was already moving before contact occurred. Therefore statements referring explicitly to the motion of the red square after contact were constructed. In the black square fixation condition there was a further sentence reading ‘Please keep your gaze on the black square all through the movie’. In the red square fixation the same wording is used except that ‘red’ was substituted for ‘black’. The experimenter verbally reminded participants of this before each movie.

Written instructions were similar to those for the non-fixation condition of Experiment 3, with two exceptions. The instructions for fixation described above were inserted, and two statements were presented for rating, as follows:

The motion of the red square after contact was brought about by the black square bumping into it.

The motion of the red square after contact was independent of that of the black square and not caused by the black square.

### 14.2. Results

#### 14.2.1. Launching measure

There was only one significant effect, the main effect of red square post-contact speed, *F* (2, 96) = 72.34, m.s.e. = 20.72, *p* < 0.001, η_p_^2^ = 0.60. Post hoc paired comparisons with the Tukey test revealed that the mean at 74.4 mm s^-1^ (6.72) was significantly higher than those at 37.2 mm s^-1^ (1.88) and 18.6 mm s^-1^ (2.08), which did not differ significantly. For the main effect of speed ratio, *F* (3, 144) = 3.05, m.s.e. = 3.28, *p* = 0.03, η_p_^2^ = 0.03. For all other effects, *F* < 1. Means are reported in table 20.

**Table 20.** Mean judgements, Experiment 7.

	black fixation			red fixation		
speed ratio	74.4	37.2	18.6	74.4	37.2	18.6
launching measure						
2:01	7.2	1.8	2.28	6.68	2.16	2.8
3:01	7.48	1.56	1.96	6.28	1.64	1.96
4:01	6.4	2.48	1.52	5.92	1.08	2.04
6:01	7.2	2.48	2.04	6.64	1.88	2.04
independent motion measure						
2:01	3.28	8.08	7.6	3.84	7.88	7.08
3:01	2.96	8.12	7.88	4.12	8.44	7.8
4:01	3.56	6.92	8.24	4.12	8.96	8.04
6:01	2.84	7.72	7.6	3.28	8.12	8.24

### 14.3. Independent motion measure

The results here were a mirror image of those on the launching measure. There was a significant main effect of red square post-contact speed, *F* (2, 96) = 57.63, m.s.e. = 22.64, *p* < 0.001, η_p_^2^ = 0.55. Post hoc paired comparisons with the Tukey test revealed that the mean at 74.4 mm s^-1^ (3.50) was significantly lower than those at 37.2 mm s^-1^ (8.03) and 18.6 mm s^-1^ (7.81), which did not differ significantly. The highest *F* ratio on any other effect was 1.51, *p* = 0.21. Means are reported in table 20.

### 14.4. Discussion

There was no significant effect of fixation (*F* < 1 on both measures) so H10 was not supported. When the speed of the red square increased after contact (74.4 mm s^-1^), launching ratings were moderately high, ranging from 6.28 to 7.48. This shows that the launching effect can occur with a chasing stimulus, i.e. one in which the red square is already in motion when contact occurs. However, if the red square continued at the same speed after contact (37.2 mm s^-1^) or slowed down (18.6 mm s^-1^), launching ratings were uniformly low (range from 1.08 to 2.48) and independent motion ratings were much higher. H9, therefore, was not supported.

## 15. Experiment 8: vertical displacement of motion path

**Figure 9.**
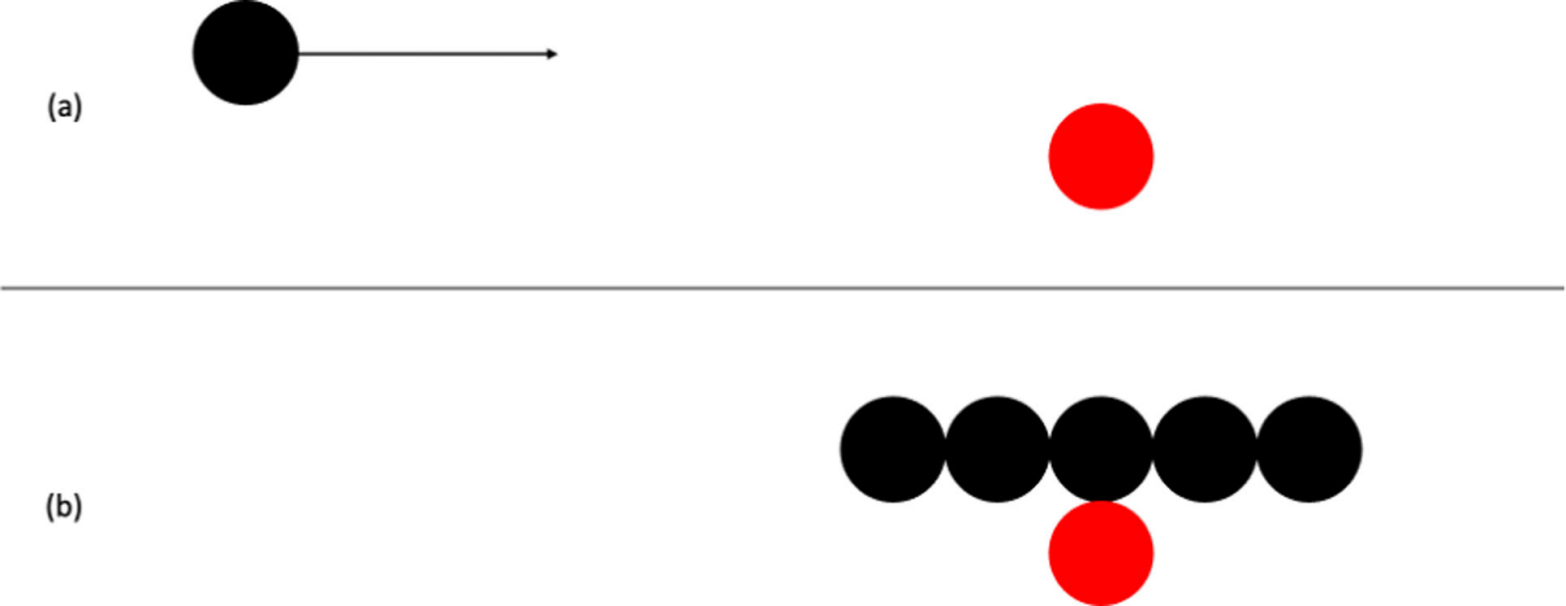
Schematic representation of stimuli used in Experiment 8. (a) Shows the first frame of the stimulus and the motion direction of the black disc. (b) Shows the five different locations at which the black disc stops. In each case, the red disc starts to move horizontally to the right as soon as the black disc stops.

In the typical stimulus for the launching effect, as depicted in figure 1, the black square contacts the red square full face on. In experiment 33, Michotte [1] used the projection method and the objects were projected discs of light. The first moving object’s path was vertically displaced. In Michotte’s words: ‘Object A sets off and takes up position immediately above or below B and in contact with it. At this moment B starts to move in its turn, and follows a route parallel to the prolongation of the route followed by A’ (p. 101). Michotte reported that the launching effect did not occur with this stimulus. This kind of displacement has not been investigated since Michotte’s research. Part of the reason for replicating the study is that it is a different type of gap stimulus. Michotte [1] and Yela [70] found that the launching effect can occur even with substantial gaps in the horizontal plane. This experiment will show whether the same is the case for gaps in a different plane of motion. This is an extended replication, with five different stopping positions for the black disc, as described in the method section and depicted in figure 9.

H11. The launching effect will be weak or absent for all stimuli.

### 15.1. Method

Michotte used discs in experiment 33, so in this experiment black and red discs with 9.3 mm radius were used instead of the black and red squares. In one movie, the black disc stopped at a point where it was vertically aligned and in contact with the red disc. In four other movies the black disc followed the same motion path but stopped two diameters before the red square, one diameter before, one diameter after and two diameters after. This is therefore a one-way ANOVA design with five values. Figure 9a shows the starting locations of the objects and the direction of the black disc’s motion. Figure 9b shows the five locations at which the black disc stopped moving. When the black disc stopped moving, the red disc moved off horizontally as the red square does in figure 1.

Wording of the statements for the participants is problematic here as well. It cannot be said that the black disc makes the red disc move by bumping into it because, in some movies, the black disc does not contact the red disc. Also, Michotte [1] reported that an impression called ‘triggering’ occurred with the displacement stimulus. This refers to an impression that one object ‘touches off’ or initiates the motion of the other object, which is nonetheless perceived as moving independently. Three statements were therefore constructed with these considerations in mind. H10 states that the launching effect will be weak or absent for all stimuli. Therefore, instead of using rating scales, participants were asked to choose the one of three verbal descriptions that best fitted with what they perceived. The prediction was that, for each stimulus, the launching description would be the least chosen. The instructions to participants read as follows:

In this experiment you will see a series of short movies, about one or two seconds in duration, each involving two objects, a black disc and a red disc. Each movie will begin with the black disc moving towards the red disc. We are interested in what you see when the black disc stops moving and the red disc starts moving, the visual impression you have of the movies, not any thoughts you might have about what you are seeing. For each movie you will be asked to choose the one of the statements listed below that best fits with your visual impression of what happened. It may still be possible to have a visual impression that the black disc made the red disc move, even when they do not come into contact. You should make your choice based just on your visual impression, not on what you think is possible.’ The three statements are as follows:

The black disc brought about the motion of the red disc.

The black disc triggered or initiated motion in the red disc, which then moved independently.

The red disc moved off when the black disc stopped moving, but it moved independently and its motion was not caused by the black disc.

### 15.2. Results

Table 21 shows the number of endorsements of each response alternative for each stimulus. Stimuli are numbered in left to right order of stopping positions as shown in figure 9b. Endorsement frequencies were analysed with the chi-square test. For stimulus 1, *χ*^2^ (2) = 12.15, *p* < 0.01. For stimulus 2, *χ*^2^ (2) = 3.63, *p* > 0.05. For stimulus 3, *χ*^2^ (2) = 3.03, *p* > 0.05. For stimulus 4, *χ*^2^ (2) = 12.26, *p* < 0.01. For stimulus 5, *χ*^2^ (2) = 75.71, *p* < 0.01.

**Table 21.** Endorsements of each response alternative, Experiment 8.

	response alternative		
stimulus	launching	triggering	independent
1	6	18	26
2	11	17	22
3	16	22	12
4	5	23	22
5	1	19	30

### 15.3. Discussion

The results were consistent with H11. There was no stimulus for which launching was the preferred endorsement. There was some tendency for launching endorsements to decline with increasing distance between the red square and the black square’s stopping location. This could be a gap effect similar to that found in Experiment 6.

For every stimulus, triggering was endorsed more frequently than launching. Michotte [1] reported triggering impressions for some stimuli. For example, with a typical stimulus for launching, if the speed of the red square was perceptibly greater than that of the black square, Michotte reported that the launching effect tended to be replaced by the triggering impression, and that this tendency increased as the speed ratio increased (experiment 40, pp. 109–110). Natsoulas [75] reported similar results. Michotte [1] stated that, in triggering, ‘there is the impression that one movement, which is otherwise clearly automonous, *depends on* the appearance of a second event which is its antecedent’ (p. 58). Hubbard [4] described it as follows: ‘in the triggering effect the launcher is perceived to release or remove inhibition on target motion, and this allows the target to begin moving of its own accord’ (p. 4). Hubbard’s description implies that it is a perceptual impression, but it is not certain that that is the case. The coincidence in time (and, to some extent, space) between the halting of the black square and the onset of motion of the red square may indicate that there must be some connection between them, but this could be more a matter of post-perceptual cognition than a perceptual impression. The present results do not permit any conclusions to be drawn on this matter and, as Hubbard’s [4] review makes clear, there has been little research on it.

## 16. Experiment 9: entraining with chasing

In experiments 48, 49 and 55, both objects were in motion from the start. The black square moved faster than the red square and caught up with it. When contact was made, the two objects moved together as in the typical stimulus for entraining. In experiment 48, they moved at the red square’s original speed. That is, the speed of the red square did not change at contact. Michotte [1] reported that the entraining effect occurred if the black square was fixated but not if the red square was fixated. In experiment 49, after contact they moved at the black square’s original speed. Michotte reported that, when there was a great difference in speed between the two objects before contact, the entraining effect occurred. When the difference in speed was small, the movements of the objects could be perceived as independent of each other. Nothing was reported about fixation. In experiment 55, after contact the two objects moved more slowly than the red square had been moving before contact. Michotte reported that the results were similar to those of experiment 49, in that the entraining effect occurred but its occurrence depended on which object was fixated. In summary, stimuli of this kind give rise to the entraining effect but not if the red square is fixated. This experiment was designed to be similar to Experiment 7 but with entraining stimuli instead of launching stimuli.

H12. There will be a main effect of fixation with higher means on the entraining measure when the black square is fixated than when the red square is fixated.

### 16.1. Method

The manipulation of motion in experiments 48 and 49 was similar to that in experiment 17, which was the model for Experiment 7, except that the black square continued to move and remained in contact with the red square after contact. For that reason, Experiment 9 was designed as an entraining version of Experiment 7. That is, the stimuli were identical to those in Experiment 7 except that, at contact, the two objects continued to move in contact with each other. The design, therefore, was a 2 between (fixation, black square versus red square) × 4 within (speed ratio, 2:1 versus 3:1 versus 4:1 versus 6:1) × 3 within (speed of both objects after contact, 74.4 versus 37.2 versus 18.6 mm s^-1^).

This is an entraining effect experiment so the wording of the statement describing a causal relation reflects Michotte’s descriptors for the entraining effect, which refer to the black square carrying or pushing the red square or taking the red square along with it [1, p. 21]. Written instructions were similar to those for the respective black square and red square fixation conditions of Experiment 7 except that two statements were presented for rating, as follows:

After contact the black square pushed the red square or carried the red square along with it.

The motion of the red square after contact was not caused by the black square.

### 16.2. Results

#### 16.2.1. Launching measure

As in Experiment 7, there was just one significant effect, a main effect of post-contact speed, *F* (2, 96) = 59.91, m.s.e. = 17.06, *p* < 0.001, η_p_^2^ = 0.56. Post hoc paired comparisons with the Tukey test revealed that the mean at 74.4 mm s^-1^ (8.21) was significantly higher than those at 37.2 mm s^-1^ (4.20) and 18.6 mm s^-1^ (4.39), which did not differ significantly. The main effect of speed ratio was not significant, *F* (3, 144) = 1.06, m.s.e. = 4.01, *p* = 0.37 η_p_^2^ = 0.02. Means are reported in table 22.

**Table 22.** Mean judgements, Experiment 9.

	black fixation			red fixation		
speed ratio	74.4	37.2	18.6	74.4	37.2	18.6
entraining measure						
2:01	7.04	3.8	3.68	8.24	5.24	4.76
3:01	7.84	3.64	4.32	8.6	4.92	5.64
4:01	8.2	3.52	3.2	8.2	4.76	5.04
6:01	8.8	3.2	3.48	8.76	4.52	5.04
independent motion measure						
2:01	3.6	6.68	7.72	1.88	6.16	6.28
3:01	3.12	6.96	6.36	1.68	5.52	5.52
4:01	2.8	7.32	7.8	1.84	5.96	6.2
6:01	1.4	7.6	7.64	1.68	6	5.76

Scrutiny of table 22 reveals that, at the two lower post-contact speeds, mean ratings appeared to be higher with fixation on the red square than with fixation on the black square. However, for the interaction between fixation and post-contact speed, *F* < 1. The main effect of fixation was also non-significant, *F* (1, 48) = 2.28, m.s.e. = 77.32, *p* = 0.14, η_p_^2^ = 0.05.

#### 16.2.2. Independent motion measure

As in Experiment 7, there was just one significant effect, the main effect of post-contact speed, *F* (2, 96) = 76.24, m.s.e. = 16.50, *p* < 0.001, η_p_^2^ = 0.61. Post hoc paired comparisons with the Tukey test revealed that the mean at 74.4 mm s^-1^ (2.25) was significantly lower than those at 37.2 mm s^-1^ (6.52) and 18.6 mm s^-1^ (6.66), which did not differ significantly. Means are reported in table 22.

#### 16.2.3. Comparison between Experiment 7 and Experiment 9

Because of the similar design of Experiments 7 and 9, it is possible to compare them directly. The experiments were presented to different participant groups, so participant group is a between-subjects variable. Data on the launching measure (Experiment 7) and the entraining measure (Experiment 9) were analysed with a 2 between (Experiment, 7 versus 9) × 2 within (fixation, black square versus red square) × 3 within (post-contact speed, 74.4 versus 37.2 versus 18.6 mm s^-1^) × 4 within (speed ratio, 2:1 versus 3:1 versus 4:1 versus 6:1) mixed design ANOVA.

There were two significant results. There was a significant effect of Experiment, *F* (1, 96) = 23.19, m.s.e. = 53.75, *p* < 0.001, η_p_^2^ = 0.19, with a higher mean in Experiment 9 (5.60) than in Experiment 7 (3.56). There was a significant effect of post-contact speed, *F* (2, 192) = 132.25, m.s.e. = 18.91, η_p_^2^ = 0.58. Post hoc paired comparisons with the Tukey test revealed that the mean at 74.4 mm s^-1^ (7.47) was significantly higher than those at 37.2 mm s^-1^ (3.04) and 18.6 mm s^-1^ (3.24), which did not differ significantly.

### 16.3. Discussion

There were no significant effects involving fixation, so H12 was not supported. Entraining ratings were significantly affected by post-contact speed, with high ratings if post-contact speed was higher than pre-contact speed and low ratings if post-contact speed was the same as or lower than pre-contact speed. There were no other significant effects. These results closely resemble those of Experiment 7. Direct statistical comparison of data from the two experiments confirmed that resemblance. Entraining ratings were significantly higher than launching ratings, indicating that the entraining impression that occurs with the stimuli in Experiment 9 appears to be stronger than the launching impression that occurs with the stimuli in Experiment 7. There were no other significant differences between the two experiments. In summary, chasing stimuli can give rise to both launching and entraining impressions if post-contact speed is greater than pre-contact speed, but both impressions are weak or absent if post-contact speed is the same as or less than pre-contact speed.

## 17. Experiment 10: entraining with relative speed manipulation

In experiment 54, relative speed before and after contact was manipulated. Michotte [1] described two variations, one in which the speed was four times faster after contact than before, and another in which the opposite was the case. Michotte reported that the entraining effect occurred with both variations: ‘this character is largely independent of a change in speed at the moment when the objects come into contact’ (p. 159). This is different from what happens with the launching stimulus, where relative speed made a considerable difference to the occurrence of the causal impression [1,75], but there has been no replication of this experiment.

H13. The entraining effect will occur for all stimuli.

### 17.1. Method

The stimuli were variations on the typical stimulus for entraining; i.e. the red square is stationary until the black square contacts it. This is an extended replication of Michotte’s experiment 54 in that three speeds were used both for motion of the black square before contact and for motion of the two conjoined objects after contact. The three speeds chosen were 62, 124 and 186 mm s^-1^. These were manipulated orthogonally for the black square before contact and the two objects after contact, resulting in a 3 × 3 design which replicates the speed ratios used by Michotte. The dependent measure asks for endorsement of one of the response options, so the chi-square test is used to analyse the data.

Written instructions were as follows:

In this experiment you will see a series of short movies, about one or two seconds in duration, each involving two objects, a black square and a red square. Each movie will begin with the black square moving towards the red square. We are interested in what you see when the black square reaches the red square, the visual impression you have of the movies, not any thoughts you might have about what you are seeing. For each movie you will be asked to choose the one of the statements listed below that best fits with your visual impression of what happened. The three statements are as follows:

After contact the black square pushed the red square or carried the red square along with it.

After contact the red square pulled or dragged the black square.

The motion of the red square after contact was not caused by the black square and the red square did not pull or drag the black square.

### 17.2. Results

Numbers of participants endorsing each response option are shown in table 23. Responses for each stimulus were analysed with the chi-square test and the results are shown in table 23. For one stimulus (62 mm s^-1^ before contact, 124 mm s^-1^ after contact) there was no significant preference. For one stimulus (62, 186 mm s^-1^), pulling was the preferred response. For the remainder there was a significant preference for entraining.

**Table 23.** Numbers of participants endorsing each option for each stimulus, Experiment 10.

		response alternative			
speed before	speed after	entraining	pulling	independent	*χ* ^2^
62 mm s^−1^	62 mm s^−1^	36	8	6	17.82**
	124 mm s^−1^	19	29	2	2.08
	186 mm s^−1^	16	31	3	4.78*
124 mm s^−1^	62 mm s^-1^	42	5	3	29.12**
	124 mm s^−1^	39	10	1	17.16**
	186 mm s^−1^	32	18	0	3.92*
186 mm s^−1^	62 mm s^−1^	40	8	2	21.34**
	124 mm s^−1^	40	8	2	21.34**
	186 mm s^−1^	44	5	1	31.04**

Note.* *p* < 0.05; ** *p* < 0.001.

To investigate this further the speed ratio (speed before : speed after) was worked out for each stimulus and this was correlated with the proportion of entraining to pulling endorsements using the Pearson coefficient of linear correlation and a significant correlation was found: *r* = +0.63, *p* < 0.05.

### 17.3. Discussion

H13 was based on Michotte’s [1] claim that the occurrence of entraining is independent of the change in speed that occurs at contact. The results show that entraining predominated for seven of the nine stimuli used in the present experiment. However for one stimulus (62, 186 mm s^-1^), pulling was the preferred endorsement. There was a significant correlation between speed ratio and proportion of entraining to pulling endorsements, showing that pulling was increasingly favoured as speed after became greater than speed before. Thus, as with launching, relative speed makes a difference to the kind of causal impression that occurs. Entraining was the favoured interpretation for most of the stimuli but not for all, so H13 is not supported.

## 18. Experiment 11

Experiments 11 and 12 together constitute an extended replication of experiment 52. Experiment 50 should be described first. In that experiment, a disc 50 mm in diameter was visible in front of a 100 × 150 mm white screen. The screen and the disc started to move horizontally at the same speed and at the same time. Michotte [1] reported that the stimulus was perceived as a single object with the disc ‘constituting “part of” the screen’ (p. 152). In experiment 52, the screen alone moved 10–20 mm and then the disc began to move, again with the same velocity as the screen. With this stimulus Michotte reported an entraining effect, with the screen pushing or carrying the disc. Michotte concluded that temporal priority of motion of the screen determined the occurrence of the entraining effect.

Michotte [1] did not report any variations on those experiments, except for one in which the disc oscillated a little while moving horizontally (experiment 51). Preliminary investigations by the present author suggested that the spatial relations between the two objects when both are in motion might make substantial and qualitative differences to the perceptual impression: the large object might be perceived as launching, pushing (entraining) or pulling the small one depending on their spatial relations. Similarity in speed of the two objects also appeared to be important to the occurrence of these impressions. Thus, the main purpose of this experiment and Experiment 12 was to replicate the stimulus used by Michotte (with adjustments necessitated by the differences in technology) and to extend the range of stimuli used, to test the possibility that qualitatively different impressions would occur depending on the spatial relations between the objects when in motion.

Experiments 11 and 12 are important for two reasons. One is that there has been no subsequent investigation of this kind of stimulus and Michotte’s experiments 50 and 52 have, as far as this author has been able to discover, never been mentioned since their publication. Michotte’s account implies that it is not necessary, for entraining to occur, that the black square should approach and contact the red square: in experiment 52, the disc is visibly superimposed on the screen, the entrainer, all the time. So replicating that result alone would add to our understanding of the entraining effect. The other reason is that the appearance of qualitative differences in perceptual impressions depending just on the spatial relations between the objects may be important to a full understanding of perceptual impressions of causality. The research literature since Michotte [1] has been heavily dominated by the launching effect and qualitatively different causal impressions have been comparatively neglected [4,7]. There is a possibility that all of them should be considered together as a single explanandum. These experiments may, therefore, shed more light on that.

H14. When both objects have the same speed, there will be qualitative differences in reported impressions with launching favoured for some stimuli, entraining for others, and pulling for others, depending on spatial relations between the objects. When the objects have different speeds, differences in reported impressions will be weak or absent.

### 18.1. Method

**Figure 10.**
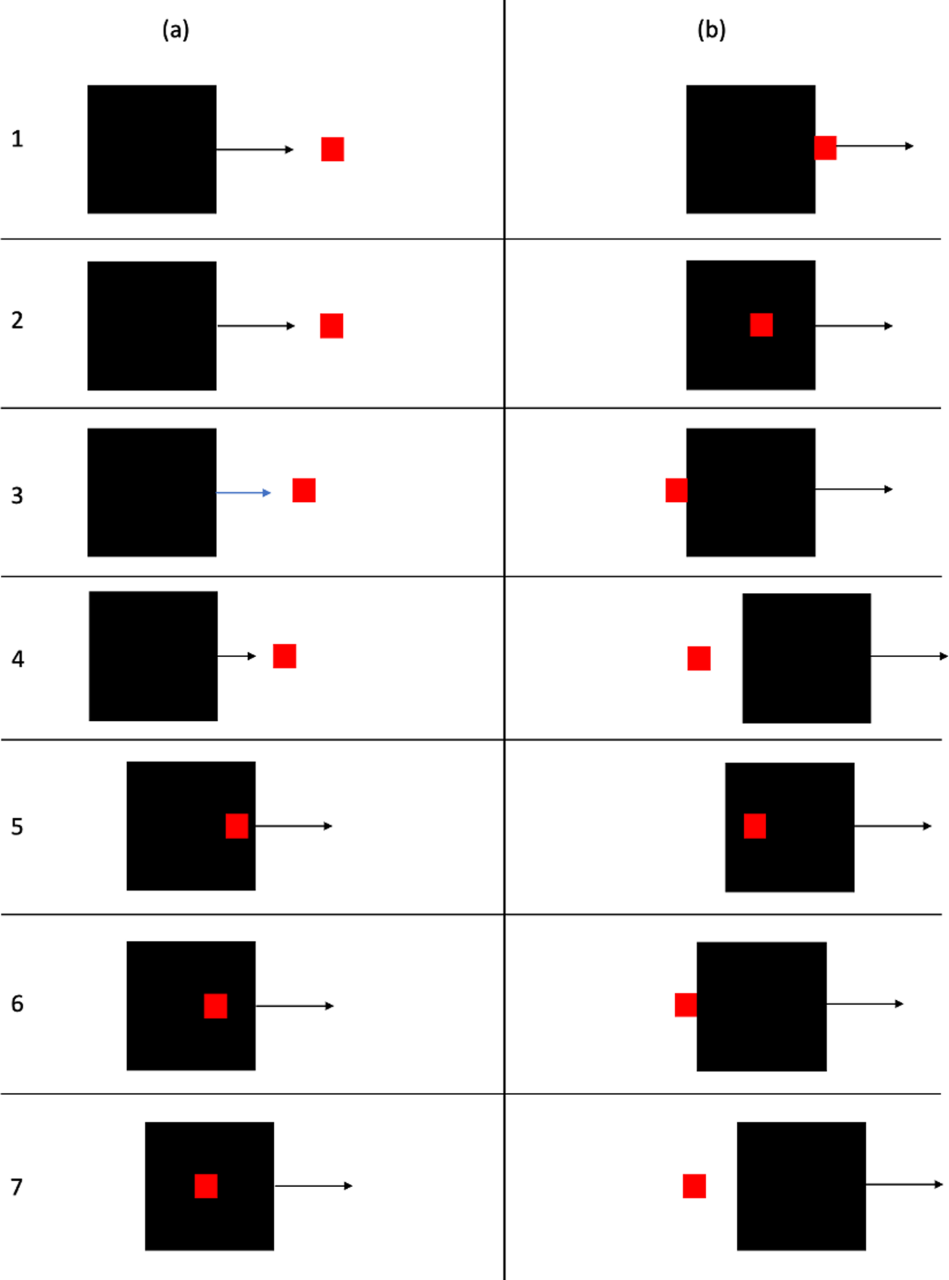
Schematic representation of seven stimuli used in Experiment 11. Stimuli are numbered from 1 to 7 and these correspond to stimulus numbers in table 24. (a) Shows the first frame of each stimulus with the motion direction of the black square indicated. (b) Shows the spatial relation between the two squares when both are in motion. When both squares move with the same velocity, the spatial relations depicted in (b) persist throughout the duration of motion of both objects. Stimulus 5 is similar to that used in Michotte’s experiment 52.

The large object in the stimuli for this research was a 186 mm black square and the small object was a 12.4 mm red square. Assuming horizontal motion of objects from left to right, and assuming that the small object starts moving at some time after the large object has started, several combinations of initial spatial relation of the objects and spatial relation when the small object starts moving are possible and were tested in this experiment. These are listed in table 24 and illustrated in figure 10 below. In addition, the speed of the small object relative to that of the large one was manipulated, being either slower, the same as, or faster. The large object moved at 124 mm s^-1^ and the small one moved at 62, 124 or 186 mm s^-1^. Orthogonal manipulation of this variable with the seven spatial arrangements described in table 24 yielded a 3 × 7 ANOVA design with a total of 21 stimuli.

**Table 24.** Spatial relations between the large object and the small object in stimuli used in Experiment 11.

1.	the small object is initially located to the right of the large object and starts to move when the large object contacts it. (This is the kinematic pattern for the typical launching stimulus.)
2.	the small object is initially located to the right of the large object and starts to move when superimposed on the large object and not in contact with any edge of it.
3.	the small object is initially located to the right of the large object and starts to move when outside but in contact with the rear of the large object.
4.	the small object is initially located to the right of the large object and starts to move when outside and beyond the rear of the large object.
5.	the small object is initially located superimposed on the large object and starts to move after a delay but when still superimposed on the large object. This is similar to Michotte’s experiment 52.
6.	the small object is initially located superimposed on the large object and starts to move when outside but in contact with the rear of the large object.
7.	the small object is initially located superimposed on the large object and starts to move when outside and beyond the rear of the large object.

Figure 10 schematically depicts the seven stimuli where both objects move at the same speed. In that figure, stimuli are numbered in accordance with their numbering in table 24, so they form a visual complement to the verbal descriptions in table 24. In figure 10, the relative sizes of the objects are not proportional to what is in the actual stimuli (because of the small size of the red square), but the spatial relations depicted are accurate. When the red square is within the boundaries of the black square, it is superimposed on the black square so that it remains visible at all times. Figure 10a shows the first frame of each stimulus. Figure 10b shows the first frame in which the red square starts to move. When both objects then move at the same speed, that spatial relation is maintained for the remainder of the stimulus. When they move at different speeds, the spatial relation is not maintained. The arrows in figure 10b represent motion of both objects, not just the large square.

**Figure 11.**
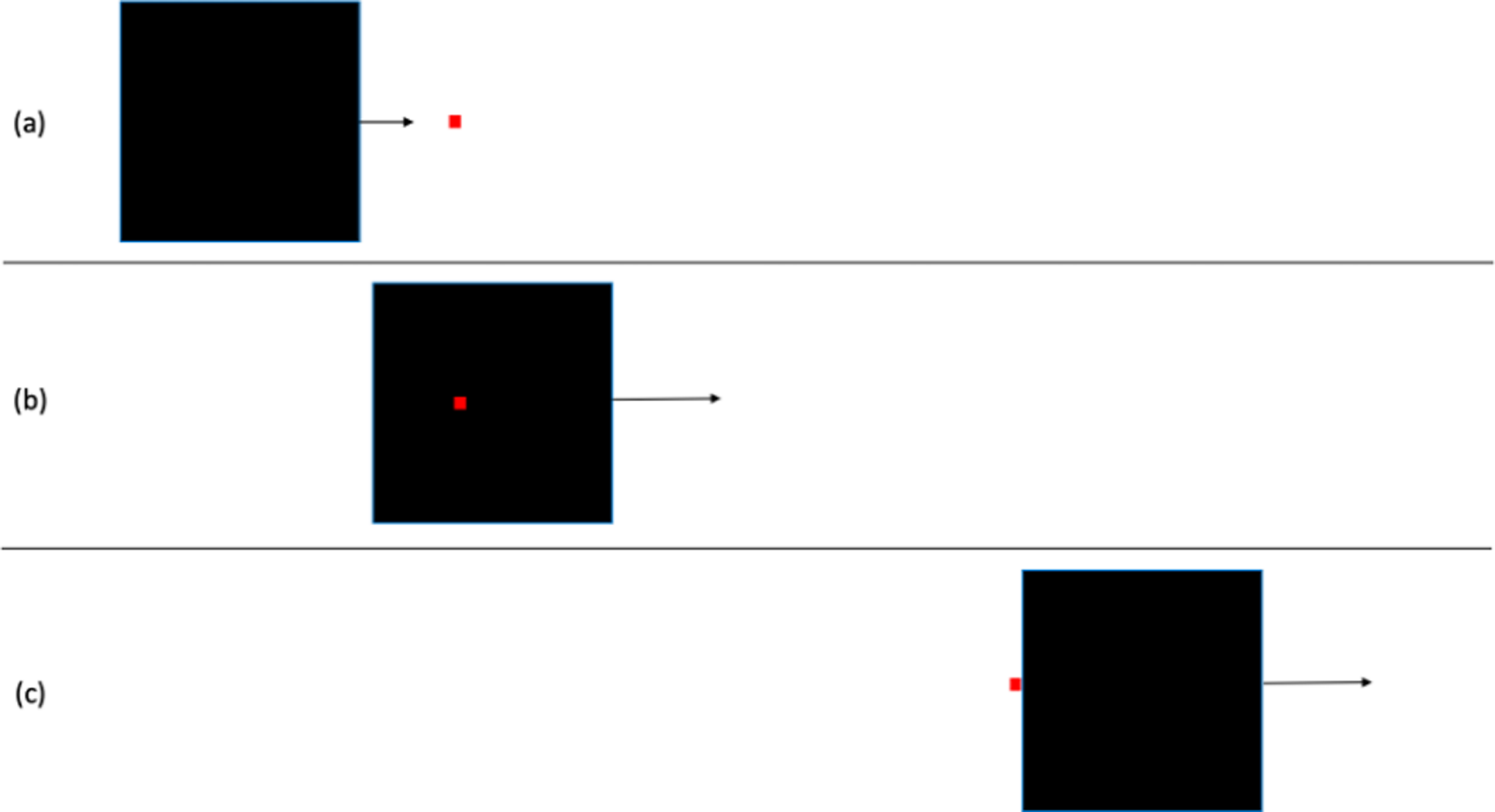
Schematic representation of a stimulus used in Experiment 11. This is number 3 as shown in figure 10 and table 24. In this figure, unlike in figure 10, the objects are shown with the correct proportional difference in size. (a) Shows the first frame with the motion direction of the black square indicated. (b) Shows an intermediate point in the motion of the black square; the red square, still motionless at this point, is superimposed on the black square so that it remains visible throughout. (c) Shows the spatial relation between the objects when both are in motion at the same speed.

An example stimulus is schematically depicted in figure 11. This is for the stimulus in which the small red square is initially located to the right of the large black square and starts to move when outside but in contact with the rear of the large square, with both objects moving at the same speed (no. 3 in table 24 and figure 10).

Stimulus no. 3 in figure 10 has kinematic features that resemble those of experiment 56, one of three experiments on what Michotte called the traction effect. The stimulus begins like a launching stimulus, and with objects of identical sizes, but the black square passes the red square; as soon as it has done so, the red square starts moving and the two objects continue in contact at the same speed as in the stimulus for the entraining effect. Michotte [1] reported that ‘we see object A pass over object B, hook it on behind and tow it’ (p. 160). So it is possible that an impression of pulling or towing may occur with this stimulus. Visual impressions of pulling have been investigated further since Michotte’s studies [76,77], and for that reason Michotte’s experiments on the traction effect were not selected for replication here. However, the stimulus emerges naturally from the manipulation of spatial relations between the objects in Experiments 11 and 12, so it is included here.

Written instructions were similar to those for Experiment 10 except that four statements were presented for rating of agreement or disagreement, as follows:

The black square made the red square move by bumping into it. (This is the descriptor for the launching effect, similar to that used in experiments on launching above.)

The black square pushed the red square or carried the red square along with it. (This is the descriptor for the entraining effect, similar to that used in experiments on entraining above.)

The black square seemed to pull the red square, as if they were connected in some way. (This is a descriptor for the pulling impression, adapted from wording used in a study of the pulling impression by [77, p. 582].

The motion of the red square was independent of that of the black square and was not caused by it in any way. (This is adapted from the independent motion descriptor used in other experiments above.)

### 18.2. Results

Data on each measure were analysed with a 3 (small object speed, 124 versus 62 versus 186 mm s^-1^) × 7 (stimuli, numbered 1 to 7 as shown in figure 10) within-subject ANOVA. As a general guide, the results show effects of all variables on all measures. However it is the results for individual stimuli, particularly those in the same speed condition, that are of most interest, and those will be considered more closely after the initial analyses have been reported.

#### 18.2.1. Launching measure

**Figure 12.**
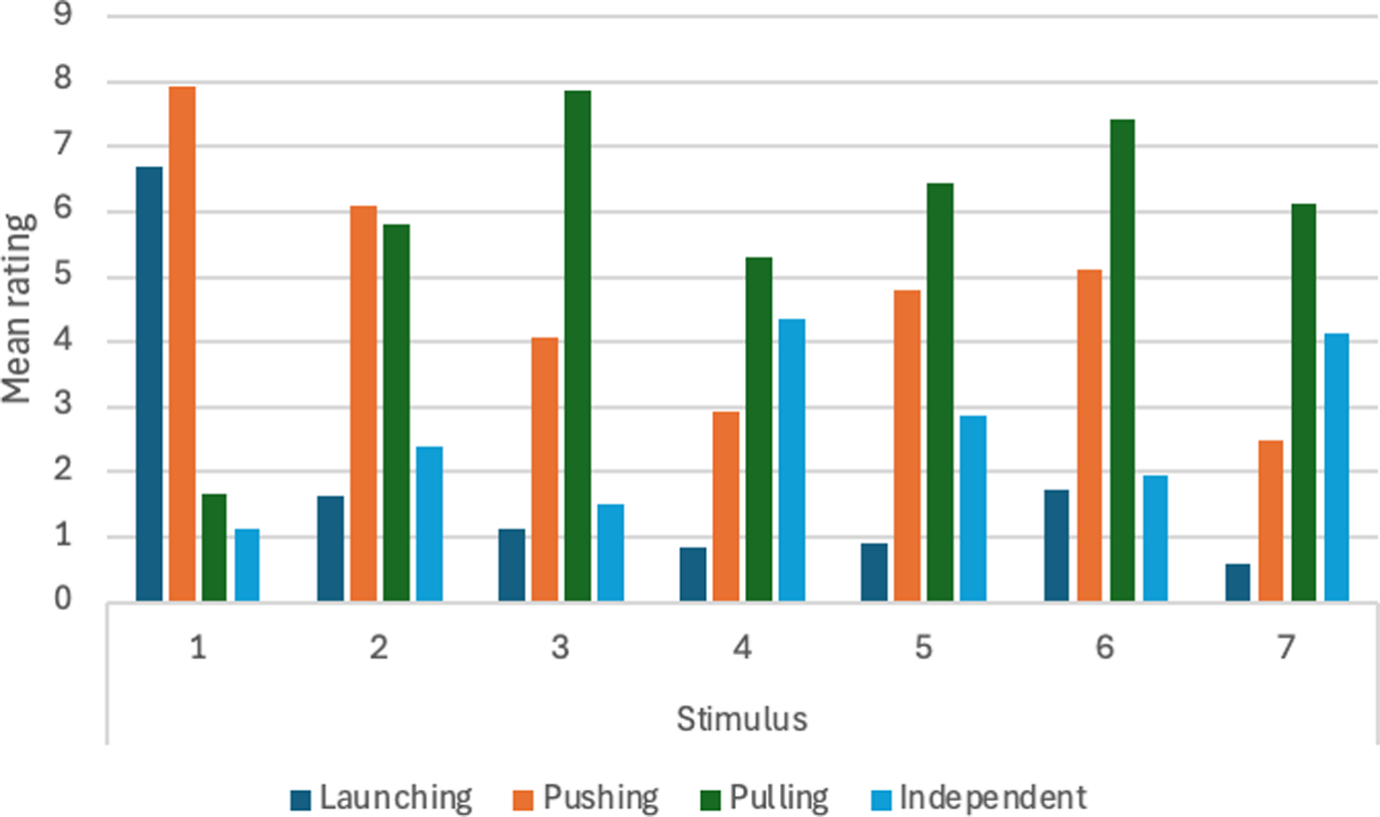
Mean ratings on all measures for the seven stimuli in which both objects move at the same speed, Experiment 11.

Means are reported in table 25, column headed ‘launching’, and depicted in figure 12. To make clear that it is small object speed relative to the speed of the first moving object that matters, speeds are identified as ‘same’ (124 mm s^-1^), ‘slower’ (62 mm s^-1^) and ‘faster’ (186 mm s^-1^).

**Table 25.** Mean judgements, Experiment 11.

		response measure			
speed	stimulus	launching	pushing	pulling	independent
same	1	6.70	7.94	1.68	1.12
	2	1.64	6.08	5.8	2.38
	3	1.12	4.08	7.86	1.5
	4	0.84	2.92	5.3	4.34
	5	0.9	4.8	6.44	2.86
	6	1.74	5.1	7.42	1.94
	7	0.6	2.5	6.12	4.12
slower	1	4.38	4.52	3.86	3.16
	2	1.86	4.36	4.36	3.78
	3	2.24	1.8	4.68	4.76
	4	0.8	1.32	4.54	4.9
	5	1.4	2.68	4.52	5
	6	1.86	2.14	4.86	4.4
	7	0.56	1.56	3.7	5.86
faster	1	8.66	4	1.3	1.16
	2	2.38	2.48	2.18	6.52
	3	2.48	2.84	3.34	4.74
	4	1.26	2.36	4	5.66
	5	2	2.62	2.5	6.02
	6	3.04	4.1	5.6	3.34
	7	1.04	1.68	4.3	5.86

There was a significant effect of small object speed, *F* (2, 98) = 19.99, m.s.e. = 6.79, *p* < 0.001, η_p_^2^ = 0.29. Post hoc paired comparisons with the Tukey test revealed a significantly higher mean at faster speed (2.98) than at same (1.93) and slower (1.87), which did not differ significantly. There was a significant effect of the seven basic stimuli, *F* (6, 294) = 75.09, m.s.e. = 7.84, *p* < 0.001, η_p_^2^ = 0.61. Post hoc paired comparisons revealed a significantly higher mean for stimulus 1 (6.58) than for the other six. In addition, stimuli 2, 3 and 6 had significantly higher means than the other three, though all means were 2.21 or lower. There was a significant interaction between speed and stimuli, *F* (12, 588) = 7.61, m.s.e. = 3.84, *p* < 0.001, η_p_^2^ = 0.13. Results of simple effects analyses are shown in table 26.

**Table 26.** Simple effects analyses, Experiment 11, launching measure.

effect	*F*	d.f.	m.s.e.	*p*	*η*_p_^2^
same speed	48.38	6, 294	4.74	<.001	0.5
slower	15.37	6, 294	5.16	<.001	0.24
faster	60.29	6, 294	5.61	<.001	0.55
stimulus 1	27.92	2, 98	8.22	<.001	0.36
stimulus 2	1.83	2, 98	3.95	0.17	0.04
stimulus 3	4.09	2, 98	6.45	<.05	0.08
stimulus 4	1.58	2, 98	2.05	0.21	0.03
stimulus 5	4.16	2, 98	3.64	<.05	0.08
stimulus 6	7.2	2, 98	3.58	<.001	0.13
stimulus 7	1.85	2, 98	1.91	0.16	0.04

#### 18.2.2. Pushing measure

Means are reported in table 25, column headed ‘pushing’, and depicted in figure 12. There was a significant effect of small object speed, *F* (2, 98) = 33.53, m.s.e. = 14.45, *p* < 0.001, η_p_^2^ = 0.41. Post hoc paired comparisons with the Tukey test revealed a significantly higher mean at same speed (4.77) than at slower (2.63) and faster (2.87), which did not differ significantly. There was a significant effect of the seven basic stimuli, *F* (6, 294) = 27.78, m.s.e. = 8.29, *p* < 0.001, η_p_^2^ = 0.36. Post hoc paired comparisons revealed a significantly higher mean for stimulus 1 (5.49) than for the other six. The mean for stimulus 2 was significantly higher than those for stimuli 3, 4 and 7. The means for stimuli 5 and 6 were significantly higher than those for stimuli 4 and 7. There was a significant interaction between speed and stimuli, *F* (12, 588) = 5.81, m.s.e. = 5.97, *p* < 0.001, η_p_^2^ = 0.11. Results of simple effects analyses are shown in table 27.

**Table 27.** Simple effects analyses, Experiment 11, pushing measure.

effect	*F*	d.f.	m.s.e.	*p*	*η*_p_^2^
same speed	20.14	6, 294	8.66	<0.001	0.29
slower	15.4	6, 294	5.6	<0.001	0.24
faster	6.53	6, 294	5.97	<0.001	0.12
stimulus 1	21.83	2, 98	10.49	<0.001	0.31
stimulus 2	24.37	2, 98	6.65	<0.001	0.33
stimulus 3	7.92	2, 98	8.22	<0.001	0.14
stimulus 4	6.04	2, 98	5.46	<0.01	0.11
stimulus 5	11.37	2, 98	6.78	<0.001	0.19
stimulus 6	12.54	2, 98	9.04	<0.001	0.2
stimulus 7	3.6	2, 98	3.64	<0.05	0.04

#### 18.2.3. Pulling measure

Means are reported in table 25, column headed ‘pulling’, and depicted in figure 12. There was a significant effect of small object speed, *F* (2, 98) = 50.46, m.s.e. = 12.12, *p* < 0.001, η_p_^2^ = 0.51. Post hoc comparisons revealed the order same (5.80)>slower (4.36)>faster (3.16). There was a significant effect of the seven basic stimuli, *F* (6, 294) = 14.54, m.s.e. = 11.95, *p* < 0.001, η_p_^2^ = 0.23. Post hoc paired comparisons revealed that the five stimuli with the highest means (3, 4, 5, 6 and 7, means ranging from 4.49 to 5.60) were not significantly different from each other. The mean for stimulus 2 (4.11) was significantly lower than that for stimulus 6. The mean for stimulus 1 (2.28) was significantly lower than all others except that for stimulus 2. There was a significant interaction between speed and stimuli, *F* (12, 588) = 8.89, m.s.e. = 6.37, *p* < 0.001, η_p_^2^ = 0.15. Results of simple effects analyses are shown in table 28.

**Table 28.** Simple effects analyses, Experiment 11, pulling measure.

effect	*F*	d.f.	m.s.e.	*p*	*η*_p_^2^
same speed	23.93	6, 294	4.75	<0.001	0.33
slower	0.98	6, 294	9.27	0.43	0.02
faster	10.64	6, 294	6.85	<0.001	0.18
stimulus 1	20.07	2, 98	4.75	<0.001	0.29
stimulus 2	28.2	2, 98	5.89	<0.001	0.37
stimulus 3	29.25	2, 98	9.21	<0.001	0.37
stimulus 4	2.19	2, 98	9.73	0.12	0.04
stimulus 5	33.11	2, 98	5.86	<0.001	0.4
stimulus 6	15.86	2, 98	7.92	<0.001	0.24
stimulus 7	11.42	2, 98	6.95	<0.001	0.19

#### 18.2.4. Independent motion measure

Means are reported in table 25, column headed ‘independent’, and depicted in figure 12. There was a significant effect of small object speed, *F* (2, 98) = 129.66, m.s.e. = 16.58, *p* < 0.001, η_p_^2^ = 0.38. Post hoc paired comparisons with the Tukey test revealed a significantly lower mean at same speed (2.61) than at slower (4.55) and faster (4.76), which did not differ significantly. There was a significant effect of the seven basic stimuli, *F* (6, 294) = 22.12, m.s.e. = 9.61, *p* < 0.001, η_p_^2^ = 0.31. Post hoc paired comparisons revealed a significantly lower mean for stimulus 1 (1.81) than for all others. The mean for stimulus 6 (3.23) was significantly lower than all the remainder except for stimulus 3 (3.67). The means for stimuli 2 (4.23), 5 (4.63) and 7 (5.28) were significantly higher than all others except for stimulus 4 (4.97). There was a significant interaction between the two variables, *F* (12, 588) = 5.98, m.s.e. = 42.07, *p* < 0.001, η_p_^2^ = 0.12. Results of simple effects analyses are shown in table 29.

**Table 29.** Simple effects analyses, Experiment 11, independent motion measure.

effect	*F*	d.f.	m.s.e.	*p*	*η*_p_^2^
same speed	11.1	6, 294	6.97	<0.001	0.18
slower	4.65	6, 294	8.3	<0.001	0.09
faster	21.49	6, 294	8.41	<0.001	0.3
stimulus 1	12.53	2, 98	5.43	<0.001	0.2
stimulus 2	23.6	2, 98	9.39	<0.001	0.33
stimulus 3	18.29	2, 98	9.62	<0.001	0.27
stimulus 4	2.23	2, 98	9.83	0.11	0.04
stimulus 5	18.17	2, 98	7.16	<0.001	0.27
stimulus 6	8.2	2, 98	9.28	<0.001	0.14
stimulus 7	6.23	2, 98	8.1	<0.001	0.11

#### 18.2.5. Analyses of individual stimuli

These are the analyses of most interest in this experiment because they reveal which kind of perceptual interpretation, if any, is favoured for each stimulus. Results of the analyses are shown in table 30 and the means for each analysis are in the corresponding rows of table 25. Table 30 is internally divided to distinguish stimuli with the same speed (nos. 1–7) from those with slower speed (nos. 8–14) and faster speed (nos. 15–21).

**Table 30.** Results of analyses of individual stimuli, Experiment 11.

stimulus	*F*	m.s.e.	*p*	*η*_p_^2^	differences
1	60.79	9.83	<.001	0.55	L & Push > Pull > I
2	23.06	11.39	<.001	0.32	Push & Pull > L & I
3	64.95	7.42	<.001	0.57	Pull > Push > L & I
4	16.52	11.36	<.001	0.25	Pull & I > Push; Pull > L
5	28.13	10.22	<.001	0.37	Pull & Push > I > L
6	35.89	10.33	<.001	0.42	Pull > Push > I & L
7	27.99	9.85	<.001	0.36	Pull > Push & I > L
8	1.38	13.72	0.25	0.03	
9	5.54	12.67	<.01	0.1	Push & Pull & I > L
10	12.32	9.99	<.001	0.2	Pull & I > Push & L
11	24.43	9.27	<.001	0.33	Pull & I > Push & L
12	13.88	10.58	<.001	0.22	Pull & I > Push & L
13	10.27	11.46	<.001	0.17	Pull & I > Push & L
14	29.28	9.49	<0.001	0.37	I > Pull > Push & L
15	85.11	7.22	<0.001	0.63	L > Push > Pull & I
16	22.03	9.89	<0.001	0.31	I > L & Push & Pull
17	3.94	12.48	<0.01	0.07	I > L & Push
18	15.91	11.63	<0.001	0.25	I > L & Push; Pull L
19	15.29	11.11	<0.001	0.24	I > L & Push & Pull
20	2.12	11.39	0.11	0.04	
21	26.11	9.74	<0.001	0.35	Pull & I > Push & L

Note. L = Launching; I = Independent motion. d.f. = 3, 147 in all analyses.

### 18.3. Discussion

Despite the large number of analyses, the results can be summarized simply. For stimuli in which both objects moved at the same speed, causal impressions of various kinds dominated. With reference to the numbering of stimuli in figure 10, the highest ratings for stimulus 1 were launching and pushing. The highest ratings for stimuli 2 and 5 were pushing and pulling. The highest ratings for stimuli 3, 6 and 7 were pulling. The highest for stimulus 4 were pulling and independent motion. For stimuli in which the objects moved at different speeds, there was only one stimulus for which one of the causal impression ratings was significantly higher than the independent motion rating. That was the version of stimulus 1 in which the red square moved faster than the black square, where the highest ratings were on the launching measure. That was the only stimulus where ratings were significantly higher on launching than on all other measures. Overall, H14 was supported. Michotte’s experiment 52 has been shown to be an exemplar of a whole class of stimuli, that has not previously been investigated, and that give rise to strong and qualitatively different causal impressions.

## 19. Experiment 12

**Figure 13.**
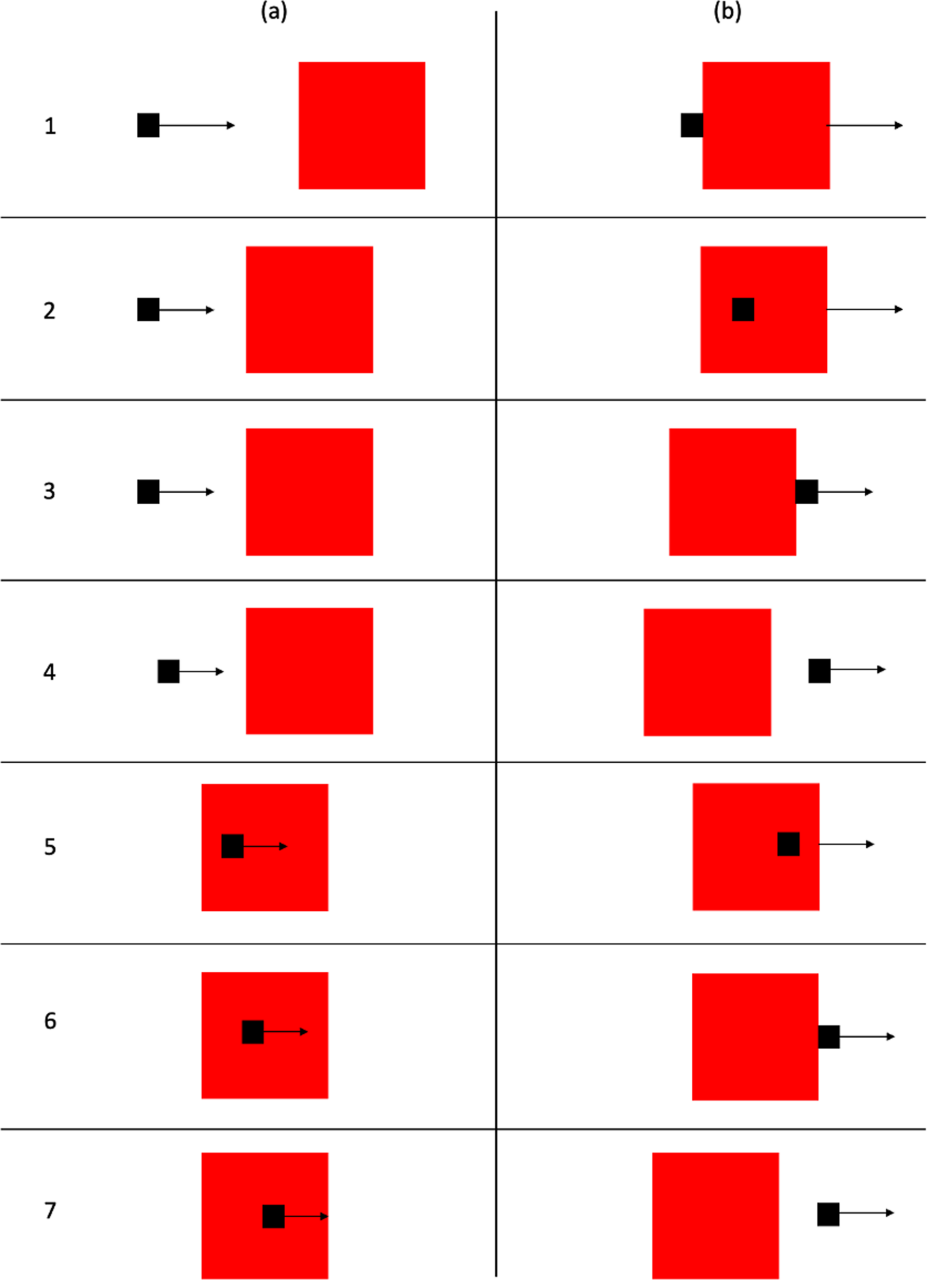
Schematic representation of seven stimuli used in Experiment 12. Stimuli are numbered from 1 to 7 and these correspond to stimulus numbers in table 31. (a) Shows the first frame of each stimulus with the motion direction of the black square indicated. (b) Shows the spatial relation between the two squares when both are in motion. When both squares move with the same velocity, the spatial relations depicted in (b) persist throughout the duration of motion of both objects.

This experiment was designed to be as similar as possible to Experiment 11 but with inversion of object size. That is, the object that moved first was now the small object. Because of the disparity in sizes, the stimuli are not quite the inverse of those used in Experiment 11. The manipulations of spatial relations are described in table 31. Schematic depictions of the stimuli are presented in figure 13 below.

**Table 31.** Spatial relations between the large object and the small object in stimuli used in Experiment 12.

1.	the large object is initially located to the right of the small object and starts to move when the small object contacts it. (This is the kinematic pattern for the typical launching stimulus.)
2.	the large object is initially located to the right of the small object and starts to move when the small object is superimposed on it and not in contact with any edge of it.
3.	the large object is initially located to the right of the small object and starts to move when the small object is outside but in contact with the front of the large object.
4.	the large object is initially located to the right of the small object and starts to move when the small object is outside and beyond the front of it.
5.	the large object is initially located with the small object superimposed on it and starts to move when the small object is still superimposed on it.
6.	the large object is initially located with the small object superimposed on it and starts to move when the small object is outside but in contact with the front of the large object.
7.	the large object is initially located with the small object superimposed on it and starts to move when the small object is outside and beyond the front of it.

H15. When both objects have the same speed, there will be qualitative differences in reported impressions with launching favoured for some stimuli, entraining for others, and pulling for others. When the objects have different speeds, differences will be weak or absent.

### 19.1. Method

Speed of the large object relative to that of the small one was manipulated, being either faster, the same as, or slower, with the same speeds as in Experiment 11. This again resulted in a 3 × 7 ANOVA design with a total of 21 stimuli. As in Experiment 11, when the small object is within the boundaries of the large one it is superimposed on the large one so as to be visible throughout. Written instructions, including the statements to be rated, were the same as in Experiment 11.

### 19.2. Results

As in Experiment 11, data on each measure were analysed with a 3 (small object speed, 124 versus 6 versus 186 mm s^-1^) × 7 (stimuli, numbered 1 to 7 as shown in figure 13) within-subject ANOVA. Results for individual stimuli are reported after these initial analyses.

#### 19.2.1. Launching measure

**Figure 14.**
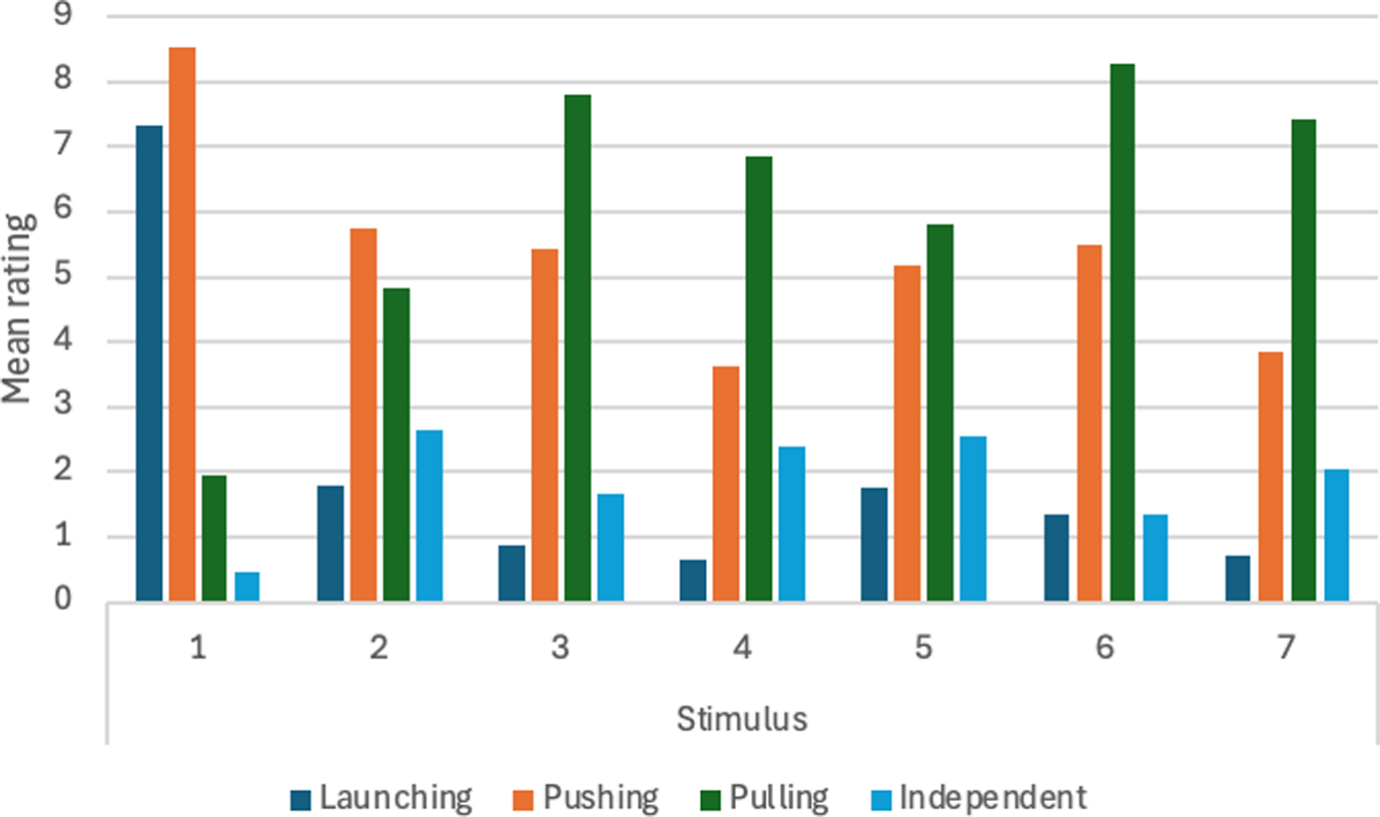
Mean ratings on all measures for the seven stimuli in which both objects move at the same speed, Experiment 12.

Means are reported in table 32, column headed ‘launching’, and depicted in figure 14. There was a significant effect of small object speed, *F* (2, 98) = 24.74, m.s.e. = 6.93, *p* < 0.001, η_p_^2^ = 0.34. Post hoc comparisons revealed the order faster (2.94) > same (2.07) > slower (1.55). There was a significant effect of the seven basic stimuli, *F* (6, 294) = 90.26, m.s.e. = 5.39, *p* < 0.001, η_p_^2^ = 0.65. Post hoc paired comparisons revealed a significantly higher mean for stimulus 1 (6.09) than for the other six. In addition, stimuli 3 (2.04) and 6 (2.23) were rated significantly higher than stimuli 4 (0.80), 5 (1.57) and 7 (0.89). Stimulus 2 (1.69) did not differ significantly from any of those. There was a significant interaction between speed and stimuli, *F* (12, 588) = 9.21, m.s.e. = 4.08, η_p_^2^ = 0.16. Results of simple effects analyses are shown in table 33.

**Table 32.** Mean judgements, Experiment 12.

		response measure			
speed	stimulus	launching	pushing	pulling	independent
same	1	7.34	8.52	1.94	0.46
	2	1.8	5.76	4.84	2.64
	3	0.88	5.42	7.8	1.68
	4	0.66	3.62	6.86	2.4
	5	1.76	5.18	5.8	2.54
	6	1.34	5.48	8.26	1.36
	7	0.72	3.84	7.42	2.04
slower	1	4.38	4.18	3.82	3.06
	2	1.58	3.72	3.96	4.94
	3	1.1	3.9	5.56	3.88
	4	0.56	2.68	5.7	4.3
	5	0.92	3	4.82	5.02
	6	1.6	3.16	5.2	4.22
	7	0.72	3.12	6	3.8
faster	1	6.54	3.62	1.12	5.06
	2	1.7	2.3	2.24	6.82
	3	4.14	4.44	3.94	2.94
	4	1.18	2.56	4.44	5.54
	5	2.04	2.74	3.28	5.94
	6	2.23	4.08	3.34	3.62
		1.22	2.38	3.82	6.58

**Table 33.** Simple effects analyses, Experiment 12, launching measure.

effect	*F*	d.f.	m.s.e.	*p*	*η*_p_^2^
same speed	70.6	6, 294	3.98	<0.001	0.59
slower	23.85	6, 294	3.59	<0.001	0.33
faster	32.61	6, 294	5.99	<0.001	0.4
stimulus 1	11.6	2, 98	10.1	<0.001	0.19
stimulus 2	0.19	2, 98	3.12	0.82	0
stimulus 3	34.65	2, 98	4.79	<0.001	0.41
stimulus 4	2.85	2, 98	1.94	0.06	0.05
stimulus 5	4.89	2, 98	3.48	<0.01	0.09
stimulus 6	14.39	2, 98	6.03	<0.001	0.23
stimulus 7	2.1	2, 98	1.98	0.13	0.04

#### 19.2.2. Pushing measure

Means are reported in table 32, column headed ‘pushing’, and depicted in figure 14. There was a significant effect of small object speed, *F* (2, 98) = 41.28, m.s.e. = 12.89, *p* <0 .001, η_p_^2^ = 0.46. Post hoc paired comparisons revealed a significantly higher mean at same speed (5.40) than at slower (3.39) and faster (3.16), which did not differ significantly. There was a significant effect of the seven basic stimuli, *F* (6, 294) = 12.44, m.s.e. = 9.03, *p* < 0.001, η_p_^2^ = 0.20. Post hoc paired comparisons revealed that the mean for stimulus 1 (5.44) was higher than those for stimuli 2 (3.93), 4 (2,95), 5 (3.64) and 7 (3.11). The means for stimuli 3 (4.59) and 6 (4.24) were significantly higher than those for stimuli 4 and 7. There was a significant interaction between speed and stimuli, *F* (12, 588) = 6.13, m.s.e. = 5.71, *p* < 0.001, η_p_^2^ = 0.11. Results of simple effects analyses are shown in table 34.

**Table 34.** Simple effects analyses, Experiment 12, pushing measure.

effect	*F*	d.f.	m.s.e.	*p*	*η*_p_^2^
same speed	15.08	6, 294	8.57	<0.001	0.24
slower	2.78	6, 294	5.32	<0.05	0.05
faster	5.82	6, 294	6.56	<0.001	0.11
stimulus 1	42.88	2, 98	8.39	<0.001	0.47
stimulus 2	23.87	2, 98	6.33	<0.001	0.33
stimulus 3	4.15	2, 98	7.15	<0.05	0.08
stimulus 4	3.22	2, 98	5.23	<0.05	0.06
stimulus 5	14.23	2, 98	6.31	<0.001	0.23
stimulus 6	8.46	2, 98	8.06	<0.001	0.15
stimulus 7	4.67	2, 98	5.7	<0.05	0.09

#### 19.2.3. Pulling measure

Means are reported in table 32, column headed ‘pulling’, and depicted in figure 14. There was a significant effect of small object speed, *F* (2, 98) = 58.34, m.s.e. = 13.42, *p* < 0.001, η_p_^2^ = 0.54. Post hoc paired comparisons revealed the order same speed (6.13) > slower (5.01) > faster (3.17). There was a significant effect of the seven basic stimuli, *F* (6, 294) = 33.66, m.s.e. = 8.01, *p* < 0.001, η_p_^2^ = 0.41. Post hoc paired comparisons revealed that stimulus 1 had a lower mean (2.29) than all others; stimulus 2 had a lower mean (3.68) than all the remainder except stimulus 5 (4.63); and there were no other significant differences (stimulus 3 = 5.77, stimulus 4 = 5.67, stimulus 6 = 5.60, stimulus 7 = 5.75). There was a significant interaction between speed and stimuli, *F* (12, 588) = 5.84, m.s.e. = 6.13, *p* < 0.001, η_p_^2^ = 0.11. Results of simple effects analyses are shown in table 35.

**Table 35.** Simple effects analyses, Experiment 12, pulling measure.

effect	*F*	d.f.	m.s.e.	*p*	*η*_p_^2^
same speed	30.22	6, 294	7.95	<0.001	0.38
slower	5.45	6, 294	6.65	<0.001	0.1
faster	11.38	6, 294	5.67	<0.001	0.19
stimulus 1	17.31	2, 98	5.53	<0.001	0.26
stimulus 2	14.75	2, 98	5.93	<0.001	0.23
stimulus 3	20	2, 98	9.39	<0.001	0.29
stimulus 4	9.74	2, 98	7.52	<0.001	0.17
stimulus 5	12.07	2, 98	6.69	<0.001	0.2
stimulus 6	43.07	2, 98	7.16	<0.001	0.31
stimulus 7	20.54	2, 98	8	<0.001	0.3

#### 19.2.4. Independent measure

Means are reported in table 32, column headed ‘independent’, and depicted in figure 14. There was a significant effect of small object speed, *F* (2, 98) = 57.95, m.s.e. = 15.29, *p* < 0.001, η_p_^2^ = 0.54. Post hoc paired comparisons revealed the order faster (4.93) > slower (4.17) > same speed (1.87). There was a significant effect of the seven basic stimuli, *F* (6, 294) = 16.02, m.s.e. = 8.73, *p* < 0.001, η_p_^2^ = 0.25. Stimulus 1 (2.19) and stimulus 3 (2.83) were rated significantly lower than all others except stimulus 6 (3.07). Stimulus 6 was rated significantly lower than stimulus 2 (4.80), stimulus 5 (4.50) and stimulus 7 (4.14). There was a significant interaction between speed and stimuli, *F* (12, 588) = 4.15, m.s.e. = 6.51, *p* < 0.001, η_p_^2^ = 0.08. Results of simple effects analyses are shown in table 36.

**Table 36.** Simple effects analyses, Experiment 12, independent motion measure.

effect	*F*	d.f.	m.s.e.	*p*	*η*_p_^2^
same speed	6.32	6, 294	4.79	<0.001	0.11
slower	2.93	6, 294	7.92	<0.01	0.06
faster	15.54	6, 294	9.04	<0.001	0.24
stimulus 1	14.29	2, 98	7.88	<0.001	0.23
stimulus 2	31.26	2, 98	7.01	<0.001	0.39
stimulus 3	7.38	2, 98	8.26	<0.01	0.13
stimulus 4	18.63	2, 98	6.71	<0.001	0.28
stimulus 5	20.89	2, 98	7.4	<0.001	0.3
stimulus 6	12.67	2, 98	8.98	<0.001	0.21
stimulus 7	32.39	2, 98	8.09	<0.001	0.4

#### 19.2.5. Analyses of individual stimuli

Results of these analyses are shown in table 37 and the means for each analysis are in the corresponding rows of table 32. Table 37 is internally divided to distinguish stimuli with the same speed (nos. 1–7) from those with slower speed (nos. 8–14) and faster speed (nos. 15–21).

**Table 37.** Results of analyses of individual stimuli, Experiment 12.

stimulus	*F*	m.s.e.	*p*	*η*_p_^2^	differences
1	113.99	6.91	<0.001	0.7	L & Push > Pull > I
2	14.95	11.44	<0.001	0.23	Push & Pull > L & I
3	64.4	8.17	<0.001	0.57	Pull > Push > L & I
4	39.14	8.74	<0.001	0.44	Pull > Push & I > L
5	18.54	10.47	<0.001	0.27	Pull & Push > I & L
6	73.93	7.74	<0.001	0.6	Pull > Push > I & L
7	51.6	8.18	<0.001	0.51	Pull > Push > I & L
8	1.3	13.01	0.28	0.03	
9	7.89	12.69	<0.001	0.14	Pull & Push & I > L
10	15.09	11.33	<0.001	0.24	Pull & Push & I > L
11	32.13	8.71	<0.001	0.4	Pull > I > Push > L
12	18.07	10.1	<0.001	0.27	Pull & I > Push > L
13	11.08	10.72	<0.001	0.19	Pull > Push & L; I > L
14	23.24	10.17	<0.001	0.32	Pull > Push & I > L
15	18.34	13.71	<0.001	0.27	L > Push & I > Pull
16	28.16	10.1	<0.001	0.36	I > L & Push & Pull
17	1.71	12.34	0.17	0.03	
18	18.06	11.15	<0.001	0.27	Pull & I > Push & L
19	13.02	11.15	<0.001	0.21	I > L & Push & Pull
20	0.35	13.28	0.79	0.01	
21	25.99	10.28	<0.001	0.35	I > Pull & Push & L; Pull > L

Note. L = Launching; I = Independent motion. d.f. = 3, 147 in all analyses.

### 19.3. Discussion

H15 was supported. As in Experiment 11, stimuli in which both objects moved at the same speed gave rise to strong causal impressions. Only in one stimulus was there a difference between the experiments in terms of the highest ratings given. In Experiment 11, for stimulus 4, pulling ratings were not significantly higher than independent motion ratings, but in this experiment they were. As in Experiment 11, the version of stimulus 1 in which the red square moved faster than the black square received higher ratings on launching than on any other measure. In addition, among the stimuli in which the objects moved at different speeds, there were three stimuli for which pulling ratings were significantly higher than all others; these were all stimuli where the red square moved more slowly than the black square.

## 20. Comparison between Experiment 11 and Experiment 12

The difference in size between the objects entails that the spatial relations between them are not exactly identical across the two experiments. Nevertheless, the designs are sufficiently similar that direct statistical comparisons between them can be carried out, and these will yield a clearer impression of the similarities and differences between the two sets of findings.

Analyses were carried out at the level of individual stimuli. Each analysis was a 2 between (Experiment 11 versus Experiment 12) × 4 within (measures, launching versus pushing versus pulling versus independent) ANOVA. Main effects of measures basically recapitulate the results already reported. There was no significant main effect of experiment in any analysis. The main interest is in the interactions. Results of these analyses are presented in table 38. They show just six stimuli with significant interactions.

**Table 38.** ANOVA results for interactions between experiment and measure, Experiments 11 and 12.

stimulus	*F*	m.s.e.	*p*	*η*_p_^2^
1	1.1	8.39	0.35	0.01
2	0.68	11.42	0.56	0.01
3	1.62	7.8	0.18	0.02
4	5.64	10.19	<0.001	0.05
5	1.11	10.35	0.35	0.01
6	1.25	9.04	0.29	0.01
7	7.12	9.02	<0.001	0.07
8	0.05	13.3	0.99	0
9	1.31	12.68	0.27	0.01
10	5.49	10.66	<0.01	0.05
11	2.21	9.24	0.09	0.02
12	0.26	10.34	0.85	0
13	0.76	11.28	0.52	0.01
14	9.35	9.83	<0.001	0.09
15	6.53	10.46	<0.001	0.06
16	0.44	9.98	0.73	0
17	5.27	12.41	<0.01	0.05
18	0.15	11.14	0.93	0
19	0.33	11.13	0.8	0
20	1.32	12.3	0.27	0.01
21	0.8	10.01	0.5	0.01

Note. df = 3, 294.

Overall, the significant interactions show a small number of minor differences that do not undermine the general conclusions to be drawn from the results of both experiments. When both objects move at the same speeds, strong causal impressions occur that differ qualitatively depending on the spatial relations between the objects when they are both in motion: launching or pushing for stimulus 1, pushing and/or pulling for stimulus 2, and pulling for all the others. The causal impressions were weaker or absent when the objects moved at different speeds. This sensitivity to differences in speed is novel and specific to the stimuli used in this experiment. In studies of launching, differences in speed before and after contact do not necessarily weaken the causal impression, and indeed Michotte [1] claimed that the launching effect was strongest when the red square moved at one quarter the speed of the black square. That contrasts with the results here where, for stimulus 1, launching ratings were higher when the contacted object moved faster than the causal object, than when it moved at the same speed or more slowly. Stimuli of the sort used in Experiments 11 and 12 therefore merit much more research and are likely to have major implications for theoretical accounts of perceptual impressions of causality. In particular, explanatory accounts that focus just on launching are inadequate, given the strong impressions of pulling and pushing that have been found in the present experiments.

## 21. Experiment 13: delay with entraining stimuli

Effects of delay and gap manipulations have featured prominently in the history of research on the launching effect but not in studies of the entraining effect [4]. Bélanger & Desrochers [78] presented entraining stimuli with either a gap of 40 mm between the objects or a delay of 1000 ms between the first object contacting the second one and the two objects starting to move together. They reported that a sample of adults perceived the typical entraining stimulus as more causal than the gap and delay stimuli but did not give any statistical information. A sample of infants aged approximately six months did not show significant discrimination between the entraining stimulus and the delay and gap stimuli. That seems to have been the only study to use a delay manipulation with entraining stimuli. Experiment 13 was therefore designed to fill this gap in the literature by replicating the delay manipulation used in Experiment 4 but with entraining instead of launching stimuli. It is predicted that the effect of delay found with launching stimuli will generalize to entraining stimuli.

H16. The entraining effect will decline as delay increases; at long delays independent motion will be perceived.

### 21.1. Method

The method was as for Experiment 4 except that entraining stimuli were used instead of launching stimuli, and the following statements were used for the rating task:

The black square pushed the red square or carried the red square along with it.

The red square seemed to pull the black square, as if they were connected in some way.

The motion of the red square was independent of that of the black square and was not caused by it in any way.

Since the two objects remain in contact in entraining stimuli, the statement referring to the red square briefly sticking to the black square before moving off was not appropriate for this experiment. The pulling impression rating was added with the exploratory aim of shedding more light on how the stimuli are perceived; there were no grounds for proposing any hypothesis about it.

### 21.2. Results

**Figure 15.**
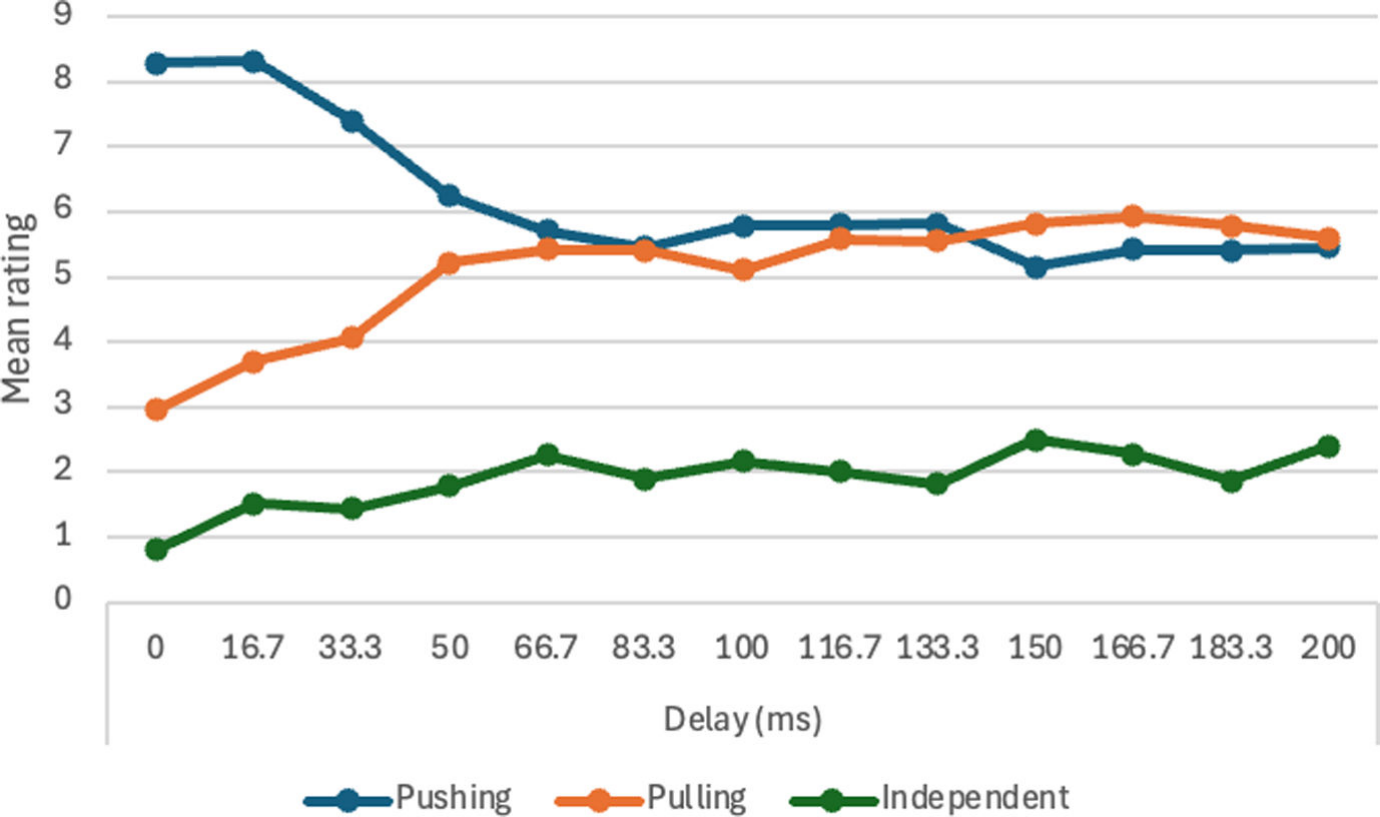
Means on pushing, pulling and independent ratings with increasing delay, Experiment 13.

Data were analysed separately for each measure with one-way ANOVA. There were significant effects of delay on all measures. On the pushing measure, *F* (12, 588) = 11.97, m.s.e. = 5.01, *p* < 0.001, η_p_^2^ = 0.20. On the pulling measure, *F* (12, 588) = 9.38, m.s.e. = 4.51, *p* < 0.001, η_p_^2^ = 0.16. On the independent motion measure, *F* (12, 588) = 3.31, m.s.e. = 3.21, *p* < 0.001, η_p_^2^ = 0.06. Means and results of post hoc paired comparisons with the Tukey test are presented in table 39, and depicted in figure 15. Results of analyses comparing the measures for each stimulus are presented in table 40.

**Table 39.** Means on all measures, Experiment 13.

delay (ms)	pushing	pulling	independent
0	8.28^a^	2.96^a^	0.80^a^
16.7	8.32^a^	3.70^ab^	1.52^ab^
33.3	7.40^ab^	4.06^abc^	1.44^ab^
50	6.24^bc^	5.22^bc^	1.78^ab^
66.7	5.70^c^	5.42^bc^	2.26^b^
83.3	5.46^c^	5.40^bc^	1.90^ab^
100	5.78^c^	5.10^c^	2.16^b^
116.7	5.80^c^	5.58^c^	2.00^b^
133.3	5.82^c^	5.54^c^	1.82^ab^
150	5.16^c^	5.82^c^	2.50^b^
166.7	5.42^c^	5.92^c^	2.28^b^
183.3	5.40^c^	5.78^c^	1.86^ab^
200	5.46^c^	5.60^c^	2.40^b^

Note. Means within columns not sharing the same superscript differ by *p* < 0.05 (Tukey).

**Table 40.** Comparisons between measures at each delay, Experiment 13.

delay (ms)	*F*	m.s.e.	*p*	*η*_p_^2^	differences
0	109.58	6.76	<0.001	0.69	Push > Pull > I
16.7	67.77	8.89	<0.001	0.58	Push > Pull > I
33.3	45.07	9.84	<0.001	0.48	Push > Pull > I
50	20.41	13.38	<0.001	0.29	Push & Pull > I
66.7	12.54	14.55	<0.001	0.2	Push & Pull > I
83.3	17.13	13.41	<0.001	0.26	Push & Pull > I
100	12.58	14.71	<0.001	0.2	Push & Pull > I
116.7	18.32	12.42	<0.001	0.27	Push & Pull > I
133.3	18.9	13.19	<0.001	0.28	Push & Pull > I
150	10.81	14.28	<0.001	0.18	Push & Pull > I
166.7	15.85	12.28	<0.001	0.24	Push & Pull > I
183.3	18.71	12.49	<0.001	0.28	Push & Pull > I
200	11.28	14.5	<0.001	0.19	Push & Pull > I

Note. I = Independent motion measure. d.f. = 2, 98.

### 21.3. Discussion

At short delays, up to 33.3 ms, ratings on the pushing measure were high and ratings on both other measures were low, lower on the independent motion measure than on the pulling measure. With delays from 50.0 ms on to 200.0 ms there was no significant difference between means on the pushing and pulling measures, but means on the independent motion measure remained low. Evidently participants perceived some kind of interaction taking place. Either they felt it involved both pushing and pulling, or some perceived pushing and others perceived pulling. The first clause in H16 is supported in that the entraining effect did decline as delay increased but only up to a delay of about 50 ms. Contrary to H16, independent motion of the objects was not perceived at any delay. The difference between these stimuli and the ones used in Experiment 4 is just that the objects both continue to move after contact, and remain in contact, whereas in Experiment 4 contact is momentary and then the black square stops moving. This simple difference has had a profound effect on how the stimuli are perceived.

## 22. Experiment 14: gap with entraining stimuli

Apart from the study by Bélanger & Desrochers [78] mentioned in connection with the previous experiment, there has been no published study of effects of gap on the entraining effect, so this study was designed to fill the gap in the literature by replicating the gap manipulation in Experiment 6 but with entraining instead of launching stimuli. It is predicted that the effects found with launching stimuli will generalize to entraining stimuli.

H17. Based on the effect of gap size on the launching effect, the entraining effect will decline as gap size increases.

H18. The entraining effect will increase in strength as speed increases.

### 22.1. Method

The method is as for Experiment 6 in all particulars except that entraining stimuli were used instead of launching stimuli.

### 22.2. Results

#### 22.2.1. Entraining measure

There was a significant effect of gap size, *F* (6, 294) = 35.77, m.s.e. = 3.90, *p* < 0.001, η_p_^2^ = 0.42. The main effect of speed was not significant, *F* (2, 98) = 4.71, m.s.e. = 5.43, *p* = 0.01, η_p_^2^ = 0.09. However there was a significant interaction between the two variables, *F* (12, 588) = 2.57, m.s.e. = 2.47, *p* < 0.01, η_p_^2^ = 0.05. Means are presented in table 41. Results of simple effects analyses are presented in table 42.

**Table 41.** Mean ratings, entraining measure, Experiment 14.

	speed (mm s^-1^)			
gap size (mm)	74.3	124	186	All
3.1	6.72	6.52	7.12	6.79^a^
6.2	5.92	5.46	6.34	5.91^b^
12.4	4.62	5	5.9	5.17^c^
24.8	4.22	5.12	4.34	4.56^cd^
46.5	4.3	4.7	4.52	4.51^cd^
68.2	4.02	4.28	4.82	4.37^d^
89.9	4.06	3.86	4.5	4.14^d^
All	4.84	4.99	5.36	

Means not sharing the same superscript differ by *p* < 0.05 (Tukey).

**Table 42.** Simple effects analyses, Experiment 14, entraining measure.

effect	*F*	d.f.	m.s.e.	*p*	*η*_p_^2^
74.3 mm s^-1^	20.77	6, 294	2.69	<0.001	0.3
124.0 mm s^-1^	12.6	6, 294	2.94	<0.001	0.2
186.0 mm s^-1^	18.47	6, 294	3.21	<0.001	0.27
Gap 3.1 mm	1.55	2, 98	3.01	0.22	0.03
Gap 6.2 mm	3.69	2, 98	2.62	0.03	0.07
Gap 12.4 mm	8.42	2, 98	2.57	<0.001	0.15
Gap 24.8 mm	3.96	2, 98	3.01	0.02	0.07
Gap 46.5 mm	0.6	2, 98	3.33	0.55	0.01
Gap 68.2 mm	2.83	2, 98	2.94	0.06	0.05
Gap 89.9 mm	1.94	2, 98	2.76	0.15	0.04

The analyses show that ratings of entraining decline as gap increases, but reach a plateau a little below the mid-point of the scale at a gap of 12.4 mm. The one significant simple effect of gap size shows mean ratings increasing as speed increased, but this was not found at other gap sizes so its generalizability might be questionable.

#### 22.2.2. Independent motion measure

There was a significant effect of gap size, *F* (6, 294) = 26.48, m.s.e. = 4.27, *p* < 0.001, η_p_^2^ = 0.35. Significant differences revealed by post hoc paired comparisons are shown in table 43. This shows a trend opposite to that found on the entraining measure, with means increasing as gap size increased, but only up to 12.4 mm. The effect of speed was not significant, *F* (2, 98) = 4.67, m.s.e. = 5.08, *p* = 0.01 η_p_^2^ = 0.09. The interaction was not significant, *F* (12, 588) = 1.62, m.s.e. = 2.82, *p* = 0.08, η_p_^2^ = 0.05.

**Table 43.** Mean ratings, independent motion measure, Experiment 14.

	speed (mm s^-1^)			
gap size (mm)	74.3	124	186	All
3.1	3.84	4.02	3.48	3.78^a^
6.2	4.48	4.92	4.06	4.49^a^
12.4	5.62	5.6	4.5	5.24^b^
24.8	6.2	5.44	5.78	5.81^bc^
46.5	5.66	5.56	5.68	5.63^bc^
68.2	6.4	5.92	5.8	6.04^c^
89.9	6.4	6.12	5.76	6.09^c^

Note. Means not sharing the same superscript differ by *p* < 0.05 (Tukey).

#### 22.2.3. Analyses of individual stimuli

Ratings of each stimulus were analysed with one-way repeated measures ANOVA and results are shown in table 44. The results show that entraining was rated higher than independent motion at the smallest gap size, but there was only one significant difference out of 18 analyses at the other gap sizes. This contrasts with the strong tendency found in Experiment 6 for independent motion to be rated higher than launching at gap sizes greater than 3.1 mm.

**Table 44.** Analyses of individual stimuli, Experiment 14.

speed	gap size	*F*	m.s.e.	*p*	*η*_p_^2^	differences
74.3	3.1	11.93	19.11	<0.01	0.2	E > I
	6.2	2.63	17.09	0.19	0.05	
	12.4	1	20.11	0.32	0.02	
	24.8	5.36	18.3	0.02	0.1	
	46.5	2.31	20.04	0.14	0.05	
	68.2	9.72	17.12	<0.01	0.17	I > E
	89.9	6.31	21.71	0.02	0.11	
124	3.1	8.45	18.49	<0.01	0.15	E > I
	6.2	0.37	19.47	0.53	0.01	
	12.4	0.79	20.16	0.38	0.02	
	24.8	0.15	17.29	0.7	0	
	46.5	0.86	21.47	0.36	0.02	
	68.2	3.77	18.28	0.06	0.07	
	89.9	5.72	21.93	0.02	0.1	
186	3.1	19.68	16.83	<0.001	0.29	E > I
	6.2	6.63	19.61	0.01	0.12	
	12.4	2.79	16.55	0.1	0.05	
	24.8	2.47	19.86	0.12	0.05	
	46.5	1.66	20.27	0.2	0.03	
	68.2	1.42	17.65	0.24	0.03	
	89.9	1.83	21.01	0.18	0.04	

Note. E = Entraining; I = Independent motion. d.f. = 1, 49.

### 22.3. Discussion

The results showed a significant tendency for entraining ratings to decline as gap size increased, but only up to a gap size of 12.4 mm. Speed had no significant effect. Only at the smallest gap size was entraining rated higher than independent motion, but at larger gap sizes neither entraining nor independent motion prevailed. The results therefore show partial support for H17 but no support for H18. Evidently the effects of manipulating gap size differ between launching and entraining.

## 23. General discussion

Table 45 presents a summary of the tests of hypotheses. The table shows mixed support: six hypotheses were supported by the results, six partly supported and six not supported. There were some significant divergences from results reported by Michotte, notably the effect of delay on the launching effect (Experiment 4), lack of effect of fixation in any experiment in which it was manipulated; lack of effect of relative speed manipulations on the entraining effect (Experiment 10); and again lack of effect of speed on entraining in Experiment 14. In addition, the results do not support the supposedly ‘paradoxical’ effects reported by Michotte with chasing stimuli: neither launching nor entraining occurred when the chased object continued at the same speed or slowed down after contact (Experiments 7 and 9). The remainder of the general discussion takes a broader look at what the results show.

**Table 45.** Summary of tests of hypotheses.

H1 (Experiment 1). Supported; passing perceived at narrowest object width with transition to launching as width increased.
H2 (Experiment 2). Partly supported. Camouflage effects found for stimuli 1, 2 and 3 but not for stimulus 4. No significant effect of fixation manipulation.
H3 (Experiment 3). Partly supported: one significant effect of object size manipulation but means were all at the low end of the scale.
H4 (Experiment 4). Partly supported. Up to delay of 98 ms, results were similar to to those reported by Michotte. At longer delays, results diverged from those reported by Michotte.
H5 (Experiment 5). Partly supported. Impression of continuous motion declined as pause
H6 (Comparison between Experiments 4 and 5). Not supported. Changes in perceptual duration increased. In other respects, results differed from those reported by Michotte.
H7 (Experiment 6). Supported: launching ratings declined as gap size increased.
H8 (Experiment 6). Supported: launching ratings increased as speed increased.
H9 (Experiment 7). Not supported: ratings of launching were low unless the red square moved faster after contact than before.
H10 (Experiment 7). Not supported: no significant effect of fixation with chasing stimuli.
H11 (Experiment 8). Supported: launching effect weak or absent for stimuli with vertical displacement of objects.
H12 (Experiment 9). Not supported: no significant effect of fixation with chasing stimuli. Also, no evidence that the entraining effect occurs if the chased object continues at the same or slower speed after contact.
H13 (Experiment 10). Not supported: relative speed before and after contact does affect the kind of causal impression that occurs.
H14 (Experiment 11). Supported. Qualitatively different causal impressions occurred with different stimuli; impressions were stronger when both objects moved at the same speed than when they moved at different speeds.
H15 (Experiment 12). Supported. Qualitatively different causal impressions occurred with different stimuli; impressions were stronger when both objects moved at the same speed than when they moved at different speeds.
H16 (Experiment 13). Partly supported. Entraining effect declined as delay increased up to 50 ms but not beyond; independent motion not perceived at any delay.
H17 (Experiment 14). Partly supported. Entraining ratings declined as gap size increased to 12.4 mm but not beyond that.
H18 (Experiment 14). Not supported. No significant effect of speed on entraining.

### 23.1. Replication

This research demonstrates the importance of replication studies. Michotte’s research was pioneering, innovative and important, but the evidential basis for perceptual impressions of causality and the factors that affect them should be established through replication and extension of the original research. There are several possible explanations for the discrepancies between what Michotte [1] reported and the present results. Methodological differences might be relevant, such as the use of computer technology instead of the rotating disc and projection methods, but there are no obvious grounds for conjecture as to how differences in technology might have affected the results. Michotte used a small sample of knowledgeable observers in many experiments, often just himself. In the present research a large sample of naive observers was used. While this might give confidence in the statistical reliability of the results, it does also raise questions about how the participants engaged with the tasks set for them. They had to read and understand instructions for the individual experiments; they had to relate what they perceived to the rating scales they were asked to fill out. Every care was taken to ensure that they reported what they perceived and not what they thought might or must be going on, but influence from post-perceptual processing cannot be ruled out. The possible effects of that on the results can only be ascertained by further research with controlled manipulations of possibly relevant features of the methods. One obvious possibility concerns the low causal ratings given to the supposedly paradoxical stimuli in which a chased object continued at the same speed or slowed after contact (Experiments 7 and 9): participants might have judged that causality was impossible under those conditions and based their ratings on that judgement, neglecting any perceptual impression they might have had. Manipulation of instructions and wording of rating scales or other measures of what is perceived could shed more light on this.

### 23.2. Launching and entraining

The type stimuli for launching and entraining are similar except that the black square stops at the point of contact in the former and continues moving at the same speed as the red square in the latter. The results of the present experiments show both similarities and differences between how equivalent launching and entraining stimuli are perceived.

To begin with the delay manipulation (Experiments 4 and 13), comparison between tables 10 and 39, and between figures 5 and 15, shows similar declines in both launching and pushing ratings as delay increased, in both cases reaching a plateau around 66.7 ms delay. The tables also show that independent motion ratings remained low at all delays for both kinds of stimuli, with a small tendency to rise as delay increased. The sticking measure in Experiment 4 and the pulling measure in Experiment 13 are not semantically equivalent so the comparison between them is not meaningful. However it seems unlikely that pulling would be perceived with launching stimuli at any delay because the black square does not move after contact.

The gap size manipulation (Experiments 6 and 14) revealed that launching and entraining ratings declined as gap size increased. The amount of decline appeared to be greater for launching than for entraining. At the largest gap size (89.9 mm), for example, the launching mean was 2.68 and the entraining mean was 4.14, so possibly the entraining impression is more resistant to the effects of gaps than the launching impression is.

The chasing stimuli used in Experiments 7 and 9 revealed generally higher ratings for entraining than for launching (table 20 for launching and table 22 for entraining). Of 24 pairs of means, mean ratings were higher for entraining than for launching on 23 of those. The difference was particularly marked for the slower post-contact speeds, where the red square moved at the same speed or slower after contact than before.

Overall, continued contact, and/or similar speeds of motion (as shown in Experiments 11 and 12) appear to foster the impression of continued interaction between the objects. Where comparison between launching and equivalent entraining stimuli is possible, there is no stimulus in the present research where launching ratings were higher than the equivalent entraining ratings but there were many where entraining ratings were higher than equivalent launching ratings. These results suggest that entraining might be a more pervasive and stronger causal impression than launching under most circumstances.

### 23.3. The pulling impression

The present experiments were designed to focus on launching and entraining because they had been the focus of most of Michotte’s research. However, the present results indicate that the pulling impression may be just as important. In particular, Experiments 11 and 12 have shown that qualitatively different causal impressions can result from small changes in spatial relations between objects when in motion at the same speed. Considering only the seven stimuli in each experiment where the two objects moved at the same speed, making 14 stimuli in all, in seven of those stimuli one kind of impression was rated significantly higher than all the others, and in all seven cases it was the pulling impression (see tables 25 and 32, first seven stimuli in each table). Stimulus 1 differs from the other six in that launching and pushing were rated significantly higher than pulling, but in no other case was pulling rated significantly lower than any of the other impressions. Michotte’s [1] report that the stimulus in his experiment 52 gave rise to an entraining effect is not supported by the results for that stimulus in Experiment 11 (stimulus 5). In Experiment 10, where entraining was predicted for all stimuli, there were two stimuli where pulling was reported significantly more often than entraining, both with stimuli where speed after contact was greater than speed before contact. There has been some previous investigation of pulling impressions [77,79] but the present results indicate that the pulling impression is more pervasive and important than has hitherto been realized. There has been no attempt to formulate an explanation for the occurrence of pulling impressions. That can be considered a major omission. In general, the results indicate that there are many possible variations in stimuli that could have profound effects on the occurrence of different kinds of causal impression, but that have yet to be explored in research.

### 23.4. Possible explanations for perceptual impressions of causality

There have been several attempts to explain perceptual impressions of causality and the present results have implications for them that will now be elucidated.

Michotte [1] argued that, in any case where a visual causal impression occurs, the motion of the target (the red square) is perceived as a continuation of the movement of the first moving object, which is perceptually independent of the spatial displacement of the target. Simplifying somewhat, the key to this is kinematic integration, which occurs when the stimulus has Gestalt properties. With the launching effect, kinematic integration depends on the Gestalt principle of good continuation [1,80]. This refers to the perpetuation of the motion properties of the first moving object in the target, which means that motion continues without a break in space or time, and without change in its properties. Thus, with a typical stimulus for launching, the launching effect is predicted to occur when the black square contacts the red square and, without delay, the red square starts moving with the same speed and direction as the black square.

Michotte’s hypothesis predicts that the launching effect should be weakened or absent if there is substantial delay at contact, gap between the objects, and vertical displacement of motion path. The results of Experiments 4, 6 and 8 gave some support to those predictions, in that the causal impression weakened significantly as both delay and gap increased. However, launching ratings were still moderate even at the longest delay in this study, and it is not clear how long a delay could be and not count as a violation of good continuation. Other results did not fit with Michotte’s hypothesis. The results for stimuli 1, 2 and 3 in Experiment 2 are contrary to what Michotte’s hypothesis would predict. In all three stimuli there was a standard launching stimulus and good continuation was present but causal ratings were low, indicating that the launching effect did not occur. This is evidently attributable to the surrounding context of motion of the red square (stimulus 2) or of other objects (stimulus 1, shown in figure 3, and stimulus 3, shown in figure 4). The occurrence of a passing impression for the narrowest objects in Experiment 1 also counts against Michotte’s hypothesis, although this result might not be disconfirmatory if it is due to limited dynamic visual acuity (see discussion section of Experiment 1).

For entraining, kinematic integration is explained by the Gestalt principle of common fate. Common fate occurs if the objects share the same motion properties after coming into contact. Thus, entraining occurs when common fate occurs after contact. This hypothesis is supported by the results of Experiments 11 and 12, where high ratings on the pushing measure were only found when both objects moved at the same speed when the second object started to move (see tables 25 and 32). The hypothesis is not supported by the results for two of the stimuli presented in Experiment 10, where speed after contact was greater than speed before contact, and pulling was reported more often than entraining. The stimuli fit the definition of common fate so those results are disconfirmatory for the common fate interpretation of entraining.

Other authors have argued that there is an innate perceptual module for the launching effect [9,20]. The module is brought into operation by definable stimulus conditions and the causal impression occurs when it operates. For the launching effect, those conditions are the typical features of the stimulus for launching, as depicted in figure 1, specifically involving minimal delay and minimal gap between the objects. The module hypothesis predicts that the launching effect should occur whenever those features are present. The hypothesis is supported by the results of Experiments 4 and 6, showing the causal impression declining as both delay and gap increase, though with the same caveat that it is not clear how long a delay or how wide a gap would be needed for the module not to be activated. However the hypothesis is disconfirmed by the results for stimuli 1, 2 and 3 in Experiment 2, where the typical features of the launching stimulus were present but the launching effect did not occur. The presence of other objects or other motions of one of the objects should not prevent the module from being activated; components of the stimulus that meet the defining conditions should be sufficient for that. No innate module for the entraining effect has been proposed.

If there is an innate module or mechanism that generates perceptual impressions of causality, it would have to be acquired on an evolutionary time scale. It would originate, therefore, in a world with minimal technology. This is a concern because these impressions occur in perception of stimuli that look as though they involve technologically sophisticated objects; billiard balls rolling on a flat surface, for example. Such things are not encountered in nature. Consider the stimuli used in Experiments 11 and 12, where qualitatively specific perceptual impressions of causality occurred with stimuli in which one object was superimposed on another. It is hard to imagine any non-technological context in which an inanimate event resembling any of the stimuli in those experiments would occur. This is a major challenge for any hypothesis in which these perceptual impressions are generated by innate mechanisms.

In two more hypotheses, perceptual impressions of causality are derived from experiences of interactions between the body and other objects. In one version, actions on objects yield information about forces and causality, mainly through proprioception [81]. Integrated proprioceptive and visual experiences of acting on objects are stored in long-term memory, where they function as a kind of template for interpreting visual information about interactions between objects [25,82]. Visual kinematic features of moving object stimuli activate stored experiences of actions on objects that have similar kinematic features. The proprioceptive component of those experiences is activated as well and functions as the perceptual interpretation of the stimuli as a causal event. The perceptual impression of causality is, in effect, the proprioceptive component. In another version, forces applied to the surface of the body are detected through proprioception; that is, instead of actions on objects, objects acting on the actor are the source of visual impressions of causality [83]. Both hypotheses depend for their testability on empirical propositions about the kinds of experience that support acquisition of causal impressions. They do not define precisely what those experiences are, and so it is not easy to generate and test predictions from either account. Brief evaluation can be offered, however.

It has been argued that the entraining effect is the kind of perceptual impression that could only result from experiences of actions on objects because the kinematics of a typical stimulus for entraining are not possible for inanimate objects [5,26]. With no change in speed at contact, entraining could only occur if the red square had zero mass and the two objects somehow became physically connected at contact, otherwise the red square would move away from the black square. The entraining effect, therefore, favours the actions on objects hypothesis.

In addition, the bodily interaction hypothesis can accommodate findings of multiple different kinds of causal impression. In the present research there was strong evidence, not only for launching and entraining, but also for pulling, especially in Experiments 11 and 12. Pulling was also reported more often than entraining for two of the stimuli in Experiment 10, where speed after was greater than speed before. Under the actions on objects hypothesis, the kinematics of a typical stimulus for pulling activate stored representations of experiences of pulling events. The pulling impression is the proprioceptive component of those stored representations applied in perceptual interpretation of the stimuli. No other hypothesis has been proposed to explain the occurrence of a pulling impression. Pulling is a peculiarly biological operation: inanimate objects do not pull each other unless one of them is driven by an internal motor and the objects are physically connected. So explaining the pulling impression without reference to experience of pulling actions would not be easy.

The camouflage effects found in Experiment 2 can be accommodated by the bodily interaction hypothesis. Stimuli 1, 2, and 3 do not match any stored representation of bodily interaction, either the body acting on something or something acting on the body. It can be argued that the stimuli either do not have any match to anything in memory, or match to events that are not related to the body. The oscillating motion of the red square in stimulus 2 might be an example of the latter, activating stored representations of oscillatory motion such as pendulum motion. Thus, non-occurrence of the launching effect with these stimuli can be explained by lack of resemblance to any stored representation of bodily interaction, or by match to some non-biological motion pattern.

## 24. Conclusion

The comprehensive review of theoretical and other issues by Hubbard [7] shows that there are many relevant matters that there is insufficient space to discuss here. The principal contribution of the present research is that it has clarified which among the results reported by Michotte [1] may be regarded as firmly established and which may not. It has also generated a set of novel results due to the extensions to Michotte’s experiments. It is to be hoped that the present set of results will inspire and give more definite direction to further testing of hypotheses to explain perceptual impressions of causality, and further investigation of the conditions under which such impressions occur. Finally, launching has dominated the research literature up to now [4], but the present research makes a case that the entraining and pulling impressions are equally important to a full understanding of perceptual impressions of causality, and it is to be hoped that both those and other qualitatively different causal impressions will be investigated more fully in the future.

## Data Availability

Data can be accessed at the OSF project for this research: [84]. This link can be found in the manuscript in the section headed 'Pre-registration and Open Science'.

## Footnotes

^1^
Another possible interpretation is that reports result from application of a decision criterion for detection, and the decision criterion might differ between stimuli of different kinds. Moors *et al*. [13] did not discuss this possibility, so further research would be necessary to test this.
